# Early environmental risk factors for neurodevelopmental disorders – a systematic review of twin and sibling studies

**DOI:** 10.1017/S0954579420000620

**Published:** 2021-10

**Authors:** Torkel Carlsson, Felix Molander, Mark J. Taylor, Ulf Jonsson, Sven Bölte

**Affiliations:** 1 Center of Neurodevelopmental Disorders (KIND), Centre for Psychiatry Research; Department of Women's and Children's Health, Karolinska Institutet & Stockholm Health Care Services, Region Stockholm, Stockholm, Sweden; 2 Child and Adolescent Psychiatry, Stockholm Health Care Services, Region Stockholm, Stockholm, Sweden; 3 PRIMA Child and Adult Psychiatry, Stockholm, Sweden; 4 Department of Medical Epidemiology & Biostatistics, Karolinska Institutet, Stockholm, Sweden; 5 Department of Neuroscience, Child and Adolescent Psychiatry, Uppsala University, Uppsala, Sweden; 6 Curtin Autism Research Group, School of Occupational Therapy, Social Work and Speech Pathology, Curtin University, Perth, Western Australia

**Keywords:** confounding factors, environmental exposure, neurodevelopmental disorders, systematic review, systematic review, twin and sibling studies

## Abstract

While neurodevelopmental disorders (NDDs) are highly heritable, several environmental risk factors have also been suggested. However, the role of familial confounding is unclear. To shed more light on this, we reviewed the evidence from twin and sibling studies. A systematic review was performed on case control and cohort studies including a twin or sibling within-pair comparison of neurodevelopmental outcomes, with environmental exposures until the sixth birthday. From 7,315 screened abstracts, 140 eligible articles were identified. After adjustment for familial confounding advanced paternal age, low birth weight, birth defects, and perinatal hypoxia and respiratory stress were associated with autism spectrum disorder (ASD), and low birth weight, gestational age and family income were associated with attention-deficit/hyperactivity disorder (ADHD), categorically and dimensionally. Several previously suspected factors, including pregnancy-related factors, were deemed due to familial confounding. Most studies were conducted in North America and Scandinavia, pointing to a global research bias. Moreover, most studies focused on ASD and ADHD. This genetically informed review showed evidence for a range of environmental factors of potential casual significance in NDDs, but also points to a critical need of more genetically informed studies of good quality in the quest of the environmental causes of NDDs.

## Introduction

Neurodevelopmental disorders (NDD) are characterized by alterations in the functioning, architecture, and maturation of the brain causing impairments in cognitive and adaptive functioning. NDDs comprise intellectual disability (ID), autism spectrum disorder (ASD), attention-deficit/hyperactivity disorder (ADHD), communication disorders (CD), specific learning disorder (SLD), and motor disorders, including developmental coordination disorder (DCD), and tic disorders (TD) (APA, 2013). The prevalence of NDDs is 10–15% of all births in the United States (Aschner & Costa, 2015), and they are increasingly being diagnosed worldwide (Elsabbagh et al., 2012). ASD and ADHD are currently the most commonly diagnosed NDDs, with prevalence estimates ranging from 0.70% up to 2.64% for ASD (CDC, 2019; Elsabbagh et al., 2012) and 5–10% for ADHD (Hansen & Rogers, 2013; Polanczyk, Willcutt, Salum, Kieling, & Rohde, 2014; Xu et al., 2018). Males exhibit NDDs more often than females, although NDDs in females might be underdiagnosed (Bargiela, Steward, & Mandy, 2016; Lai et al., 2017). NDD phenotypes are heterogeneous, and their complexity is compounded by high comorbidity rates with several conditions (i.e. other psychiatric disorders, neurological and immunological disorders, gastrointestinal disturbances, and congenital anomalies) (Muskens, Velders, & Staal, 2017; Simonoff et al., 2008). In face of the substantial individual burden and the societal costs these conditions incur on public health care, educational, and long-term support systems, it is of paramount importance to identify specific factors involved in the etiology of NDDs that might facilitate earlier detection and open up for earlier interventions (Bellinger, 2012; Grandjean, Pichery, Bellanger, & Budtz-Jørgensen, 2012; Trasande & Liu, 2011).

The causes of NDDs are multiple, both genetic and environmental (Martin, Taylor, & Lichtenstein, 2018; Taylor et al., 2019), but, the exact causes driving atypical neurodevelopment remain poorly understood. Based on findings from twin and family studies, NDDs are considered highly heritable (Polderman et al., 2015; Posthuma & Polderman, 2013; Ronald & Hoekstra, 2011), with both common and rare genetic variants contributing to the phenotypes (Hansen & Rogers, 2013). While the research focus has until recently been mostly on genetic causes (Bauxbaum & Hof, 2011; Demontis et al., 2019; Landrigan, Lambertini, & Birnbaum, 2012; Szatmari et al., 2007), heritability estimates leave space for the potential significance of environmental factors (Herbert, 2010; Pessah, Cherednichenko, & Lein, 2010; Shelton, Hertz-Picciotto, & Pessah, 2012; Zuk et al., 2012). In addition, for several NDDs, such as ASD and ADHD, clinical phenotypes, broader phenotypes, and traits of the conditions are continuously distributed in the general population, with overlapping etiologies and sources of variation (Martin et al., 2018). Therefore, it is also important to look at outcomes of NDD as both categorical (diagnoses) and dimensional (traits and symptoms) for two reasons. First, as dimensional definitions in contrast to categorical ones may be more sensitive to subtle subclinical toxic effects, they may enable the development of more detailed exposure–response profiles and facilitate the testing of complex functional relationships between continuous behavior measures and biological outcomes like brain structure and behavior (Rauh & Margolis, 2016). Second, because the etiology of clinical phenotypes overlaps with the etiology of subclinical phenotypes and condition traits, studying those traits might generate heuristic hypotheses to be tested in clinical samples.

Animal, human cell, and epidemiological studies suggest a wide range of environmental risks impact on neurodevelopment. Recently, prenatal maternal anemia has been associated with several NDDs, including ID, ASD, and ADHD (Wiegersma, Dalman, Lee, Karlsson, & Gardner, 2019). In ASD, associations with advanced parental age, maternal valproate intake during pregnancy, toxic chemical exposure, maternal diabetes, enhanced steroidogenic activity, immune activation, possibly altered zinc-copper cycles, and treatment with selective serotonin reuptake inhibitors (SSRI) during pregnancy have been reported (Bölte, Girdler, & Marschik, 2019). Environmental factors commonly linked to ADHD are food additives/diet, lead contamination, cigarette and alcohol exposure during pregnancy, and low birth weight (Banerjee, Middleton, & Faraone, 2007). For reading disabilities, Mascheretti, Andreola, Scaini, and Sulpizio (2018) found evidence for gestational age and birth weight being the most important pre- and perinatal risk factors, while reporting inconclusive findings for maternal cigarette smoking, family history of psychiatric and medical diseases, and risk of miscarriage. Prenatal alcohol consumption, diabetes, treatment with antidepressants, being deficient in iodine or iron, and dietary fish, as well as postnatal depression, low birth weight, and neonatal problems have all been linked to motor difficulties in childhood (Golding, Emmett, Iles-Caven, Steer, & Lingam, 2014). Pregnancy-related noxious exposures and lower birth weight may be more frequent in pregnancies of children who later develop Tourette's syndrome, particularly maternal smoking and prenatal life stressors, and psychosocial stress influences tic severity (Hoekstra, Dietrich, Edwards, Elamin, & Martino, 2013). With regards to developmental mechanisms, research from different disciplines found alterations of key biological systems in NDDs, such as catecholaminergic imbalances, glutamatergic synapse function, chromatin modelling, and ion channel pathways (Cristino et al., 2014; Geschwind & Levitt, 2007; Pinto et al., 2014). It is suggested that changes to immunological, endocrinological, and gut–brain axis processes are involved in causal pathways (Edmiston, Ashwood, & Van de Water, 2017; Kelly, Minuto, Cryan, Clarke, & Dinan, 2017).

Familial confounding is a major limitation to much of the current literature on environmental risk factors. Familial confounders are shared factors within a family, including both unmeasured shared environmental and genetic factors, that increase similarity in siblings. Although many of the above environmental factors have been shown to be associated with NDDs, many of the exposures are in themselves, to a degree, heritable. Thus, it cannot be ruled out that they are driven by genetic links between exposure and outcome, and not by the environment itself. As discussed by van Dongen, Slagboom, Draisma, Martin, and Boomsma (2012) and D'Onofrio, Lahey, Turkheimer, and Lichtenstein (2013b), twin, sibling, and family studies, as compared to conventional case control studies, have the potential to disentangle the effects of environment from genetic and unknown environmental factors. By comparing the risk of a given outcome in twins or siblings who are differentially exposed to a given factor—or conversely, comparing exposure across pairs who are discordant for the outcome—it is possible to adjust for many factors that are shared *within* the pairs of twins or siblings. This has often been neglected in previous research on environmental factors in NDD. Indeed, making causal inference with confidence requires far more prerequisites than just control for familial confounding (Hill, 1965; Sjölander & Zetterqvist, 2017). Still, this type of adjustment has proven highly useful in refuting proposed causal associations. For example, a meta-analysis by Mezzacappa et al. (2017) estimated the odds ratio [*OR*] for ASD to be 1.52 (95% CI, 1.09–2.12) for SSRI exposure during pregnancy. However, a later epidemiological study found that this association was to a large degree confounded by familial factors since it was attenuated in a sibling comparison analyses (Rai et al., 2017). Likewise, regarding the above listed potential environmental risk factors for ADHD, a more recent review by Sciberras, Mulraney, Silva, and Coghill (2017) revealed a pattern indicating that the stronger the study design—especially regarding genetic and familial confounders—the less likely it was to support an association of SSRI use in pregnancy and the presence of ADHD in offspring. Similarly, the strong association between ADHD and smoking during pregnancy seem to be better accounted for by genetic and familial factors rather than a causal association between smoking during pregnancy and ADHD. This is key to understanding the rationale behind twin and sibling studies. Other analytical approaches assume that there are no concurrent explanations of the associations among initial risks, the mediating variables, and the outcome of interest (in this case smoking during pregnancy and ADHD), although it is clearly the case in reality. First, other environmental risks such as parental intellectual abilities, socioeconomic status (SES), and psychiatric problems also predict offspring ADHD; second, smoking during pregnancy is influenced by genetic factors (D'Onofrio et al., 2013b). The same also holds true for many other environmental factors and makes studies controlling for familial confounding a crucial aspect when trying to establish causal inference.

When comparing sibling and twin studies, the within-pair comparisons among twins, in particular those in monozygotic (MZ) twins, hold the best premises for adjustment for familial confounding when studying environmental risks. Despite this, there are several valid reasons why sibling studies are occasionally preferred over twin studies. First, it is rarely possible to measure prenatal differences in twins sharing the same prenatal environment. In order to be able to perform an analysis of the within-pair association of a prenatal factor with a particular outcome, one would require individual and separate prenatal exposure information for each twin, an often-impossible demand. In some instances, as in the case of gestational age, there is no within-pair difference to measure. Therefore, we are left with studying siblings from different pregnancies, regarding prenatal exposures, when trying to adjust for familial confounding. Second, siblings are more common than twins, and therefore, sibling studies are easier to perform, and larger cohorts possible to collect. Third, replication of results in both twins and siblings ensures that the results obtained from twin studies generalize beyond twins.

This systematic review spans from pregnancy-related factors to the early childhood, inviting the investigation of the timing related to the effect of potential environmental factors. Interestingly, studies have shown that the heritability of fetal growth rate changes across trimesters (Workalemahu et al., 2018), and that the heritability of autistic traits changes from childhood to early adulthood (Taylor, Gillberg, Lichtenstein, & Lundström, 2017). These are examples pointing to the possibility that the controlling for familial confounding might be differentially important during different stages of development.

The aim of this systematic review was to summarize the evidence from twin and family studies about the role of environmental risk factors for NDDs, defined both dimensionally and categorically, controlling for familial confounding, in order to inform researchers and funding agencies in both preclinical and applied areas of NDDs, and guide clinical management. The potential costs of environmental factors being incorrectly connected to NDDs, owing to a lack of control of familial confounding in research, include waste of public resources, unnecessary worry, misleading advice, and eroded public trust. The broad and systematic approach of this review, incorporating all NDDs according to the fifth edition of *Diagnostic and Statistical Manual of Mental Disorders* (DSM-5) nomenclature, allowed us to map a wide range of environmental factors postulated to be involved in their etiology and identify factors that have not yet been sufficiently studied in relation to NDDs.

## Method

This systematic review was conducted and reported in accordance with the Preferred Reporting Items for Systematic Reviews and Meta-Analyses (PRISMA) Statement (Moher, Liberati, Tetzlaff, & Altman, 2009). The protocol was registered in advance with PROSPERO (CRD42018079513).

### Search strategy

A systematic literature search was performed by two librarians at Karolinska Institutet in October 2017 in the following databases: Medline (Ovid), PsycInfo (Ovid), Embase, Web of Science Core Collection and Cochrane Library. The search was updated in March 2019 for recently published articles. The complete search strategy for each database is available in Supplementary Appendix 1.

### Eligibility

Study design: Case control and cohort studies including a twin or sibling comparison. Case control studies should include twins or siblings discordant for one or more NDDs, with the unaffected or less affected twin or sibling as the comparator. Cohort studies should include twins or siblings discordant for exposure and with one or more NDD as the outcome.

Exposure: Any specified environmental factor, with exposure time up to the age of 5 years. Only studies with a specified environmental factor were included.

Outcome: One or more of the NDDs included in DSM-5 (ASD, ADHD, ID, CD, SLD, DCD, and TD). The conditions could either be reported as categorical (diagnoses) or dimensional (symptom or traits severity). Categorical outcomes were defined according to DSM-III, DSM-IV, DSM-5, International Classification of Diseases (ICD-9), ICD-10, or earlier diagnostic practices, and based on clinical assessment, medical registries, or cut-offs for diagnosis on diagnostic instruments (APA, 1987, 2000, 2013; NCHS, 1990; WHO, 1992). Dimensional outcomes were defined using disorder specific scales, or scales measuring constructs closely related to the respective conditions. Eligible studies should report the within-pair association of the exposure with one or more NDD, or with symptom or traits severity. Studies only reporting on the heritability in general terms were excluded.

Publication type: Peer-reviewed articles published in English.

### Study selection and data extraction

After removal of duplicates the titles and abstracts of the studies retrieved from the search were screened using EndNote X8 and X9. The titles and abstracts of all references were screened independently by two reviewers. At this stage, a publication was excluded if the reviewers unanimously found that it was clear that it did not meet the given eligibility criteria. Publications found to be of potential relevance by at least one of the reviewers were obtained in full text and assessed for eligibility independently by two reviewers. Disagreement at this stage was solved by consensus. If necessary, a third reviewer was consulted.

The main study characteristics and results were extracted independently by two reviewers. A data extraction sheet was created, pilot tested, and modified based on the Cochrane EPOC Data Collection Checklist (Higgins et al., 2011). Discrepancies were solved by consensus. Items extracted included: author; publication year; country; study design; study cohort; sample size; sex; age; sibling or twin control; disorder /-s studied; environmental factor /-s studied; study methodology; recruitment method; completion rates; missing data; outcomes and type of measures; and the main results.

### Risk of bias assessment

The overall risk of bias of each study was rated according to the Newcastle–Ottawa Scale (NOS) for longitudinal case control and cohort studies (Wells et al., 2019). Three quality domains (selection, comparability, and exposure) and additional subdomains according to the NOS were assessed. Subdomains for case control studies were: adequacy of the case, representativeness of the cases, selection of controls, definition of controls, comparability of cases and controls on the basis of the design or analysis, ascertainment of exposure, same method of exposure ascertainment in cases and controls, and nonresponse rate. Subdomains for cohort studies were: representativeness of the exposed cohort, selection of the nonexposed cohort, ascertainment of exposure, demonstration that outcome of interest was not present at start of study, comparability of cohorts on the basis of the design or analysis, assessment of outcome, follow-up long enough for outcomes to occur, and adequacy of follow-up of cohorts. The NOS scores range from 0 to 9, with one point given for each subdomain reaching a predefined quality threshold (except for “comparability” where a maximum of 2 points could be allotted). It was modified to fit the bias assessment for twin and sibling studies, so that only twin studies could reach the maximum of 2 points for the comparability criterion. Consequently, sibling studies could not exceed 8 points. Studies with a score of 2 points or below were excluded. The quality of each study was assessed independently by two reviewers. If discrepancy could not be solved by consensus, a third reviewer was consulted.

### Synthesis

Identified environmental factors were sorted according to chronology (prenatal; perinatal/neonatal; and infancy/childhood) and grouped by category for readability. For studies with categorical NDD outcomes, the relevant estimated association(s) were extracted. The estimates presented in the studies were sorted based on the type of measure of association used (hazard ratio; odds ratios; relative risks; and other). When no estimated association was reported, available data were used to calculate such estimates if possible. Since studies with dimensional measures routinely reported several estimated associations, an evaluation of these studies was conducted to determine if the overall findings provided a signal of an association or not (yes; possibly; or no).

A narrative synthesis of the eligible studies for each NDD was performed, with separate presentations of studies with categorical and dimensional outcomes. For environmental factors represented in more than one study for a specific condition, a judgement was made on the importance of the exposure across studies. The judgement was based on the estimated associations and the risk of bias in the respective studies. When appropriate, meta-analyses of the results on specific environmental factors and conditions were conducted, unless prevented by heterogeneity of the included studies’ exposures, study characteristics, or data presentation (Higgins & Green, 2011).

## Results

### Study selection

**Figure 1.**
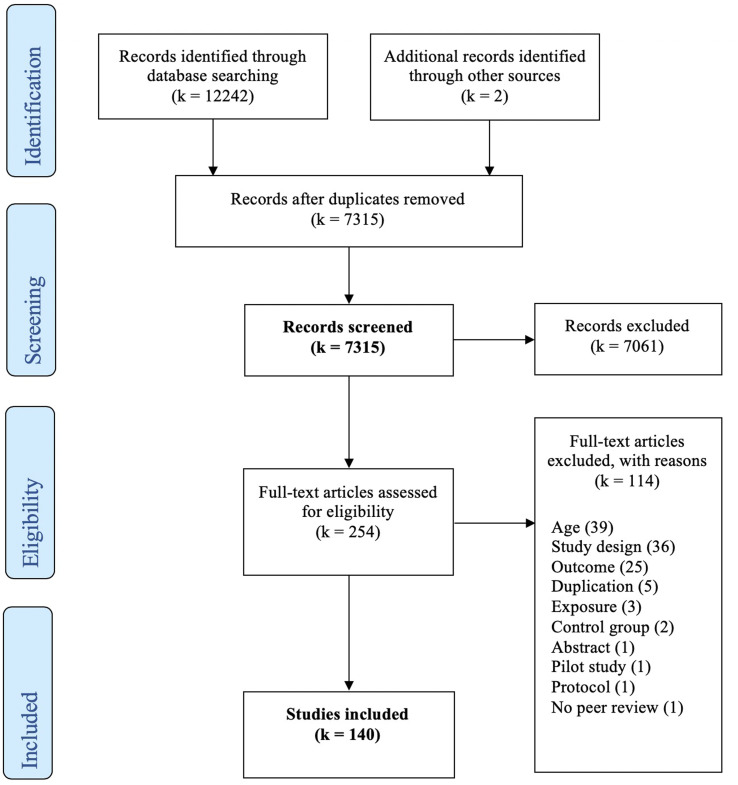
The 2009 PRISMA Flow Diagram

A total of 140 studies were identified for inclusion (Figure 1). The search provided 7,315 unique citations. Two additional studies were identified from reference lists in published articles. After reviewing the abstracts, 7,061 citations were discarded in the preliminary screening, mainly due to not being consistent with the defined study design. The remaining 254 citations were examined in full text; of these 114 did not meet the eligibility criteria and were excluded (see Supplementary Appendix 2 available online). All included studies reported on *within-pair* associations, referred to as “association” below.

### ASD

#### Study characteristics

In total, 58 studies (22 cohort studies and 36 case control studies) on ASD were included (see Table 1 for full list of references). All studies used a categorical definition of ASD, except for one study that used a dimensional outcome (Ronald et al., 2010) and one that used both (Willfors et al., 2017). The studies were published between 1971 and 2019, with a steady increase from the year 2000. The studies were predominantly conducted in Scandinavia (*k* = 25) and North America (*k* = 18). The majority were sibling studies (*k* = 51), and seven were twin studies. The number of cases in the case control studies ranged from 5 to 1,133, with a median of 72, while the number of analyzed siblings or twins in the cohort studies ranged from 68 to 2 665,666, with a median of 921. Prospectively collected data were used in all but two of the cohort studies, and in approximately half of the case control studies. Regarding age at diagnosis, all but three of the cohort studies lacked information for the sibling subsamples, as well as a majority of the case control studies. When considering the general methodology of the complete samples, the risk of misclassification bias due to age of diagnosis was deemed low. The sex distribution among differently exposed sibling or twins was not reported in the majority of the cohort studies, while this distribution was skewed towards male cases in the case control studies. The NOS score for study quality ranged from three to nine, with more consistently high scores for cohort studies. Typical reasons for downgrading the study quality were ascertainment of exposure and definition of controls. See Table 1.

**Table 1.** Study characteristics—autism spectrum disorder (ASD)

COHORT STUDIES		Exposed population of twins/siblings				Unexposed population of twins/siblings					
Author (year)	Study design	Outcome/s	Data source	*N* (% ♀)	Age *M* (*SD*); range	Comparator	*N* (% ♀)	Age *M* (*SD*); range	Country	Recruitment	NOS-score
Aagaard, Bach, Henriksen, Larsen, and Matthiesen (2018)	Pop. based, pro.	ASD, ADHD	Registry data	–^a^		Sibling			Denmark	Database linking	8
Bilder et al. (2013)	Pop. based, pro.	ASD	Clinical.	392 (–)		Sibling	529 (–)		USA	Database linking	8
Brown et al. (2017)	Retro.	ASD	Registry data	620 (–)		Sibling	620 (–)		Canada	Database linking	8
Class, Rickert, Larsson, Lichtenstein, and D'Onofrio (2014)	Pop. based, pro.	ADHD, ASD	Registry data	–^b^		Sibling	–		Sweden	Database linking	8
Class et al. (2018)	Pop. based, pro., quasi-exp.	ASD, ADHD	Registry data	346,739 (–)^c^		Sibling	1,050,271 (–)		Sweden	Database linking	8
Curran et al. (2015)	Pop. based, pro.	ASD	Registry data	2,555 (–)^d^		Sibling	2,555 (–)		Sweden	Database linking	8
DeVilbiss et al. (2017)	Pop. based, pro.	ASD	Registry data	–^e^		Sibling	–		Sweden	Database linking	8
D'Onofrio et al., (2013a)	Pop. based, pro., quasi-exp.	ASD, ADHD	Registry data	–^f^		Sibling	–		Sweden	Database linking	8
D'Onofrio et al., (2014b)	Pop. based, pro.	ASD, ADHD	Registry data	–^g^		Sibling	–		Sweden	Database linking	6
Gardner et al. (2015)	Pop. based, pro.	ASD	Registry data	605 (–)^h^		Sibling	605 (–)		Sweden	Database linking	8
Hamad, Alessi-Severini, Mahmud, Brownell, and Kuo (2018)	Pop. based, pro.	ASD	Registry data	57,063 (–)^i^		Sibling	57,063 (–)		Canada	Database linking	7
Hultman, Sandin, Levine, Lichtenstein, and Reichenberg (2011)	Pop. based, pro.	ASD	Clinical.	660 (–)^j^		Sibling	–		Sweden	Database linking	8
Losh, Esserman, Anckarsäter, Sullivan, and Lichtenstein (2012)	Pop. based, pro.	ASD	Parental interview	34 (26)	9 and 12 y	Twin	34 (26)	9 and 12 y	Sweden	Database linking	9
Oberg et al. (2016)	Pop. based, pro.	ASD	Registry data	–^k^		Sibling	–		Sweden	Database linking	8
Parner et al. (2012)	Pop. based, pro.	ASD	Registry data	2,732 (20)^l^		Sibling	3,972 (36)		Denmark	Database linking	8
Pettersson, Larsson, D'Onofrio, Almqvist, and Lichtenstein (2019)	Pop. based, pro.	ASD, ADHD	Registry data	546,894 (49)^m^	27.2; 15.1– 40.9	Sibling	546,894 (49)	27.2; 15.1– 40.9	Sweden	Database linking	9
Rai et al. (2017)	Pop. based, pro.	ASD	Registry data	175 (–)^n^		Sibling	175 (–)		Sweden	Database linking	7
Ronald, Happé, Dworzynski, Bolton, and Plomin (2010)	Pop. based, pro.	*ASD-traits*	Parental and teacher quest.	5,796 (52)	7 and 8 y	Twin	5,796 (52)	7 and 8 y	UK	All twin families in UK and wales via letter	7
Sørensen et al. (2013)	Pop. based, pro.	ASD	Registry data	96 (–)^o^		Sibling	6,046 (–)		Denmark	Database linking	7
Sujan et al. (2017)	Pop. based, retro.	ASD, ADHD	Registry data	10,975 (–)^p^		Sibling	13,994 (–)		Sweden	Database linking	7
Tillman et al. (2018)	Pop. based, pro.	ASD, ADHD, ID, CD	Registry data	6,844 (42)^q^		Sibling	9,355 (48)		Sweden	Database linking	8
Yang et al. (2017)	Pop. based, pro.	ASD	Registry data	2,687 (–)^r^		Sibling	2,792 (–)		Denmark	Database linking	7

CASE CONTROL STUDIES		Study population of twins/siblings				Control population of twins/siblings					
Author (year)	Study design	Outcome/s	Data source	*N* (% ♀)	Age *M* (SD); range	Comparator	*N* (% ♀)	Age *M* (SD); range	Country	Recruitment	NOS-score
Abd Elhameed, Abd Elbaky, and Kamel (2011)	Retro.	ASD	Clinical.	14 (14)	10.9	Sibling	28 (–)	–	Egypt	From clinical project	5
Arora et al. (2017)	Pro., deeply phenotyped	ASD, *ASD-traits*	Clinical.	32 (38)^s^	14.7; 10.8–18.6	Twin	32 (38)	14.7; 10.8–18.6	Sweden	From twin register and via advertisement	8
Bolton, Pickles, Harrington, Macdonald, and Rutter (1992)	Pro.	ASD	Medical record review	196 (–)		Sibling	121 (–)		UK	From a clinical practice	6
Brown, Austin, and Busija (2014)	Retro., pilot	ASD	Clinical database	19 (21)		Sibling	23 (42)		Australia	Database linking	3
Bryson, Smith, and Eastwood (1988)	Pop. based, pro.	ASD	Medical record review	17 (29)	9.8	Sibling	10 (60)		Canada	General Pop. screening	7
Burd et al. (1988)	Retro.	PDD-NOS	Clinical.	50 (34)	9 (4.6)	Sibling	–	8.8 (–)	USA	From all North Dakotas development evaluation centers	4
Chien et al. (2018)	Retro.	ASD	Clinical.	323 (9)	10.7 (–)	Sibling	257 (57)	11.7 (–)	Taiwan	From two clinical sites	6
Creagh et al. (2015)	Retro.	ASD	Registry data	262 (24)		Sibling	253 (50)		Puerto Rico	Database linking	3
Dawson, Glasson, Dixon, and Bower (2009)	Pop. based, pro., nested	ASD	Registry data	465 (16)		Sibling	481 (48)		Australia	Database linking	8
Deb, Prasad, Seth, and Eagles (1997)	Pop. based, retro.	ASD	Clinical.	30 (30)		Sibling	30 (50)		UK	All children from the Aberdeen city area	6
Deykin and MacMahon (1980)	Retro.	ASD	Parental interview	145 (19)		Sibling	326 (50)		USA	From referrals	6
Deykin and MacMahon (1979)	Retro.	ASD	Parental interview	163 (16)		Sibling	355 (50)		USA	From referrals	5
Fernell et al. (2015)	Pro.	ASD	Clinical.	58 (10)		Sibling	58 (60)		Sweden	General Pop. screening	5
Finegan and Quarrington (1979)	Pro.	ASD	Clinical.	23 (17)		Sibling	15 (–)		Canada	From collaborative perinatal study	4
Froehlich-Santino et al. (2014)	Pro- and retro.	ASD	Clinical.	137 (ND)		Twin	137 (ND)		USA	From CATS^t^	8
Glasson et al. (2004)	Pop. based, pro.	ASD	Registry data	465 (16)		Sibling	481 (48)		Australia	Database linking	6
Gong et al. (2019)	Pop. based, pro., nested	ASD	Registry data	1,133 (25)^u^	13.4 (3.3)	Sibling	1,293 (49)	13.9 (3.4)	Sweden	Database linking	7
Grossi, Migliore, and Muratori (2018)	Retro., AFN	ASD	Clinical.	35 (18)	7.9 (–)	Sibling	45 (26)	8.9 (–)	Italy	From two clinical practices	6
Hadjkacem et al. (2016)	Pro- and retro.	ASD	Parental interview	50 (26)	–; 3–7	Sibling	51 (–)	–; 3–12	Tunisia	From a clinical practice	6
Hagberg, Robijn, and Jick (2018)	Pop. based, pro., nested	ASD	Database linking	531 (14)		Sibling	601 (14)		UK	Database linking	7
Isaksson, Pettersson, Kostrzewa, Diaz Heijtz, and Bölte (2017)	Retro.	ASD	Parental web survey	206 (22)	Median: 7 y	Sibling	209 (47)	Median: 6 y	Sweden	Through advertisement	3
Lord, Mulloy, Wendelboe, and Schopler (1991)	Pro- and retro.	ASD^v^	Medical record review	46 (50)	18 (–)	Sibling	54 (52)		USA	Through a systematic contacting of clinics	7
Manning, Baron-Cohen, Wheelwright, and Sanders (2001)	Pro.	ASD	Parental quest.	72 (14)	–; 2–15	Sibling	34 (–)		UK	From the National Autistic Society in UK	7
Manohar, Pravallika, Kandasamy, Chandrasekaran, and Rajkumar (2018)	Retro.	ASD	Clinical. and quest.	30 (13)	4.13 (1.8)	Sibling	30 (10)	4.32 (1.7)	India	From a clinical practice	5
Mason-Brothers et al. (1990)	Pop. based, pro.	ASD	Clinical.	233 (21)		Sibling	66 (45)		USA	Database linking	7
Mason-Brothers et al. (1993)	Pop. based, pro.	ASD	Clinical.	233 (21)		Sibling	66 (45)		USA	Database linking	6
Myers, Van't Westeinde, Kuja-Halkola, Tammimies, and Bölte (2018)	Pro., deeply phenotyped	ASD, ADHD	Clinical.	46 (–)^w^		Twin	192 ()		Sweden	From twin registry and via advertisement	8
Oerlemans et al. (2016)	Retro.	ASD, ADHD	Parental quest.	152 (22)	–; 11.3–12	Sibling	136 (38)	–; 10.9- 12.1	Netherlands	Through clinics and the Dutch Autism Association	7
Otake et al. (2006)	Pro.	ASD	Clinical.	5 (20)	18.4 (–)	Sibling	7 (57)	21.3 (–)	Japan	ND	5
Piven et al. (1993)	Pro- and retro.	ASD	Clinical.	39 (18)	14.5; 5–25	Sibling	39 (44)	14.5; –	USA	From the Maryland Society for Autistic Adults and Children	6
Rutt and Offord (1971)	Pro.	ASD	Unclear	33 (18)	–; 5.3–13.8	Sibling	83 (40)	–	USA	From a clinical practice	6
Steffenburg et al., (1989)	Pop. based, retro.	ASD	Clinical.	22 (45)	12; 2–23	Twin	22 (45)	12; 2–23	Sweden, Norway, Denmark, Finland and Iceland	Through pediatric and child psychiatric clinics in Scandinavia	8
Stevens, Fein, and Waterhouse (2000)	Pro.	ASD	Clinical.	175 (17)	–; 3–6.9	Sibling	100 (48)		USA	From referrals	6
Willfors et al. (2017)	Pro- and retro.	ASD, *ASD-traits*	Clinical.	MZ: 54 (44) DZ: 46 (–)	MZ: 14.9; 8–28 DZ: 14.3; 8–25	Twin	MZ: 54 (44) DZ: 46 (–)	MZ: 14.9; 8–28 DZ: 14.3; 8–25	Sweden	From twin registry and via advertisement	8
Williams, Hersh, Allard, and Sears (2008)	Retro.	ASD	Clinical.	15 (0)	–; 2–6	Sibling	16 (25)	–; 2–6	USA	From a clinical practice	5
Zwaigenbaum et al. (2002)	Pop. based, retro.	ASD, PDD-NOS	Clinical.	78 (38)	8.7 (–)	Sibling	88 (50)	8.9 (–)	Canada	From clinical practice and from the Autism Society of Ontario	7

Dimensional outcomes in *italics*. “–” and “ ”  = not reported.

Abbreviations: *N* = number of subjects, *M* = mean, *SD* = standard, deviation, pop. based = population based, pro.= pro. exposure data, retro. = retrospective exposure data, quasi-exp. = quasi-experimental, AFN = artificial neural networks, ASD = autism spectrum disorder, ADHD = attention-deficit/hyperactivity disorder, ID = intellectual disability, CD = communication disorders, PDD-NOS = pervasive developmental disorder, not otherwise specified, Clinical. = clinical assessment, Quest. = questionnaire, MZ = monozygotic, DZ = dizygotic

a All births in Denmark between 1997 and 2013, in total differently exposed 8,156 siblings in analysis.

b All births in Sweden between 1973 and 2008.

c All births in Sweden between 1987 and 2007, with in total 368,549 third-born siblings.

d All births in Sweden between 1982 and 2010.

e Stockholm Youth Cohort born 1996 and 2007, in total 174,428 in the ASD sibling cohort.

f All births in Sweden between 1973 and 2008, in total 2,665,666 siblings in analyses.

g All births in Sweden between 1973 and 2001, including offspring from 1,408,669 distinct fathers and 1,404,484 distinct mothers.

h Stockholm Youth Cohort born 1984 and 2007, in total 4,775 siblings in the analysis with 605 differently exposed.

i All births identified in the Manitoba Health Insurance Registry between 1 April 1998 and 31 March 2016.

j All births in Sweden between 1983 and 1992 and followed up until 2002.

k All births in Sweden between 1992 and 2005, in total 694,612 in sibling analysis.

l All births in Denmark between 1980 and 2003, 16,588 children from 7,005 families in sibling cohort.

m All births in Sweden between 1973 and 1998.

n Stockholm Youth Cohort born all individuals aged 0–17 living in Stockholm County in 2001–11 (*n* = 735 096).

o All births in Denmark 1996 and 2006 (*n* = 668,468). For SSRI: 81 exposed and 6,036 unexposed.

p All births in Sweden between 1996 and 2012. For SSRI: 9,063 exposed and 15,906 unexposed.

q All births in Sweden between 1973 and 2012.

r All births in Denmark between 1998 and 2008.

s 17 MZ and 15 DZ twin pairs.

t From the California Autism Twin Study (CATS).

u All births in Sweden between 1992 and 2007.

v High-functioning autism.

w Ages at examination ranged from 8 to 29 years (*M* = 16.2, *SD* = 5.2). In total 70 MZ pairs and 49 DZ pairs.

#### Prenatal exposure

The included studies examined a total of 42 prenatal exposures, 18 of which were investigated in more than one study (Table 2). Three factors were identified with predominantly positive findings. Advanced paternal age was found to be associated with ASD in three large population-based sibling cohort studies with HR (95% CI) between 1.39 (1.01–1.90) and 3.45 (1.62–7.33) and *F* (3, 631) = 2.40, *P* = .049 (D'Onofrio et al., 2014b; Hultman et al., 2011; Parner et al., 2012), while a small sibling case control study with a higher risk of bias failed to replicate this finding (Hadjkacem et al., 2016). Similarly, two population-based twin cohort studies (Losh et al., 2012; Willfors et al., 2017) and two population-based sibling cohort studies (Class et al., 2014; Pettersson et al., 2019) found associations for low birth weight with HR 2.44 (95% CI, 1.99–2.97), *OR* (95% CI) between 3.25 (1.47–7.18) and 1.38 (1.31–1.44) and Z = − 2.20, *p* = .028, while three sibling case control studies with higher risk of bias did not (Chien et al., 2018; Mason-Brothers et al., 1990; Oerlemans et al., 2016). A similar pattern was seen for birth defects were two large population-based studies, one cohort and one case control, found an association with HR 1.3 (95% CI, 1.0–1.7) and *OR* 1.5 (95% CI, 1.0–2.3) (Dawson et al., 2009; Tillman et al., 2018), while one sibling case control study with higher risk of bias did not (Mason-Brothers et al., 1990). Mixed findings were reported for antidepressive medication during pregnancy (*k* = 5 studies) (with only one reporting a positive association (Rai et al., 2017)), advanced maternal age (*k* = 4), rubella infection during pregnancy (*k* = 2), birth order (*k* = 2), gestational weight gain (*k* = 2), stress during pregnancy (*k* = 2), as well as for a composite scores of prenatal complications (*k* = 9). No statistically significant within-pair associations were reported regarding maternal uterine bleeding (*k* = 4), maternal infection during pregnancy (*k* = 3), season of birth (*k* = 3), preeclampsia (*k* = 3), prenatal testosterone level (*k* = 2), urinary tract infection (*k* = 2), gestational diabetes (*k* = 2), and pre-pregnancy body mass index (*k* = 2). All these studies reported low effect sizes, except for the smaller of the two case control studies on urinary tract infection that reported a medium effect size (Hadjkacem et al., 2016). An additional 24 factors were investigated in single studies. These studies found associations of ASD with measles and mumps infections during pregnancy, an interpregnancy interval of more than year, metal uptake in uterus (lead and manganese), low serum level of vitamin D at birth, and a parity greater than two.

**Table 2.** Environmental factors, prenatal—autism spectrum disorder (ASD)

Environmental factor		Author (year)	*N*	HR (95% CI)	*OR* (95% CI)	RR (95%CI)	Other	NOS
***Prenatal***								
Maternal medication	Any	Mason-Brothers et al. (1990)	233				*χ*^2^: ns	7
	Antidepressant	Brown et al. (2017)	620	1.60 (0.69 to 3.74)				8
		Sørensen et al. (2013)	96	1.1 (0.5 to 2.3)				7
		Sujan et al. (2017)	10,975	0.83 (0.62 to 1.13)^a^				7
		Rai et al. (2017)	175		**1.69 (1.06 to 2.72)**			7
		Hagberg et al. (2018)	531			1.53 (0.89 to 2.62)		7
	Antibiotics	Isaksson et al. (2017)	206		5.93 (0.27 to 128.82)^b^			3
	Systemic β2-agonists only	Gong et al. (2019)	1,133		0.80 (0.45 to 1.43)^c^			7
	Inhaled β2-agonists	Gong et al. (2019)	1,133		0.94 (0.61 to 1.47)^c^			7
	Other asthma medications	Gong et al. (2019)	1,133		0.74 (0.42 to 1.31)^c^			7
Paternal medication	SSRI before conception	Yang et al. (2017)	2,687	0.74 (0.34 to 1.59)				7
Antenatal nutritional supplementation^d^		DeVilbiss et al. (2017)	–^e^	0.77 (0.52 to 1.15)			8	
Prenatal viral exposure	Infection	Oerlemans et al. (2016)	152		2.72		*p* = .114	7
		Isaksson et al. (2017)	206		0.98 (0.17 to 5.45)^b^			3
		Mason-Brothers et al. (1990)	233				*χ*^2^: ns	7
	Measles	Deykin and MacMahon (1979)	163			**5.5**	***p* = .0412**	5
	Mumps	Deykin and MacMahon (1979)	163			**5.5**	***p* = .0474**	5
	Rubella	Chien et al. (2018)	323		0.75 (0.07 to 8.32)			6
		Deykin and MacMahon (1979)	163			**3.3**	***p* = .0044**	5
	Chickenpox	Deykin and MacMahon (1979)	163			1.7	*p* = .1677	5
Toxic exposure	Mercury	Williams et al. (2008)	15				*p* = .62	5
	PCB	Otake et al. (2006)	5				*p* > 0.05	5
	Solvents/paints	Grossi et al. (2018)	35		2.56 (0.76 to 8.72)			6
	PVC	Grossi et al. (2018)	35		1.47 (0.50 to 4.3)			6
	Tap water (cupper)	Grossi et al. (2018)	35		2.19 (0.6 to 7.4)			6
Metal uptake in uterus	Manganese	Arora et al. (2017)	32				***r* = −.25 (−.40 to −.10)**	8
	Lead	Arora et al. (2017)	32				***r* = .40 (.20 to .60)**	8
Smoking during pregnancy		Oerlemans et al. (2016)	152		1.11		*p* > 0.05	7
Advanced parental age	Both parents	Oerlemans et al. (2016)	152		1.07		*p* > 0.05	7
	Paternal	Hultman et al. (2011)	660				***F* (3, 631) = 2.40, *p* = .049**	8
	age>45	D'Onofrio et al., (2014b)	**–**^f^	**3.45 (1.62 to 7.33)**				8
	age>40^g^	Parner et al. (2012)	2,732	**1.39 (1.01 to 1.90)**				8
	age>35	Hadjkacem et al. (2016)	50				*p* = .12	6
	Maternal age	Mason-Brothers et al. (1990)	233				*χ*^2^: ns	7
		Abd Elhameed et al. (2011)	14				***χ*^2^, *p* = .05**	5
		Piven et al. (1993)	39				*χ*^2^: ns	6
	age>35	Hadjkacem et al. (2016)	50				*p* = .59	6
Birth order		Zwaigenbaum et al. (2002)	78				ns^h^	7
	Firstborn^i^	Oerlemans et al. (2016)	152		**6.48 (1.88 to 22.33)**			7
Interpregnancy Interval	0–5 months	Class et al. (2018)	346,739	0.76 (0.54 to 1.07)				8
	6–11 months	Class et al. (2018)	346,739	0.79 (0.62 to 1.01)				8
	12–23 months	Class et al. (2018)	346,739	**0.76 (0.61 to 0.95)**				8
Low birth weight		Class et al. (2014)	**–**^j^	**2.44 (1.99 to 2.97)**				8
		Chien et al. (2018)	323		3.94 (0.82 to 18.92)			6
		Losh et al. (2012)	34		**3.25 (1.47 to 7.18)**			9
		Pettersson et al. (2019)	546,894		**1.38 (1.31 to 1.44)**			8
		Willfors et al. (2017)	100				**Z = −2.20, *p* = .028**	8
		Mason-Brothers et al. (1990)	233				*χ*^2^: ns	7
		Oerlemans et al. (2016)	152			ns		7
Head circumference at birth		Aagaard et al. (2018)	–^k^	0.97 (0.91 to 1.02)				8
Prenatal testosterone levels	Higher 2D:4D ratio	Manning et al. (2001)	72				β = 0.29, *F* = 1.91, *p* = .18	7
		Myers et al. (2018)	46				β = −.009 (−.024 to .005)	8
Malformations		Dawson et al. (2009)	465		**1.5 (1.0 to 2.3)**			8
		Mason-Brothers et al. (1990)	233				*χ*^2^: ns	7
	Orofacial clefts	Tillman et al. (2018)	6,844	**1.3 (1.0 to 1.7)**^b^				8
Season of birth		Stevens et al. (2000)	175				*χ*^2^: ns	6
		Bolton et al. (1992)	196				*χ*^2^:18.44 (11df), *p* = .07	6
	Winter or summer birth	Abd Elhameed et al. (2011)	14				*χ*^2^, *p* = .2	5
Low vitamin D at birth		Fernell et al. (2015)	58				**t57 = 2.57, *p* = .013, *d* = 0.33**^l^	5
Maternal medical conditions	Uterine bleeding	Chien et al. (2018)	323		1.50 (0.17 to 13.60)			6
		Glasson et al. (2004)	465		1.10 (0.56 to 2.16)			6
		Mason-Brothers et al. (1990)	233				*χ*^2^: ns	7
		Piven et al. (1993)	39				*χ*^2^: ns	6
	Preeclampsia	Chien et al. (2018)	323		1.51 (0.74 to 3.10)			6
		Glasson et al. (2004)	465		0.92 (0.56 to 1.49)			6
		Mason-Brothers et al. (1990)	233				*χ*^2^: ns	7
	Hypertension	Piven et al. (1993)	39				*χ*^2^: ns	6
	Premature membrane rupture	Glasson et al. (2004)	465		1.33 (0.60 to 2.95)			6
	Urinary tract infection	Hadjkacem et al. (2016)	50		5.7 (0.8 to 38.2)			6
		Glasson et al. (2004)	465		0.98 (0.50–1.92)			6
	Gestational diabetes	Chien et al. (2018)	323		3.43 (0.57 to 20.82)			6
		Piven et al. (1993)	39				*χ*^2^: ns	6
	Untreated depression	Hagberg et al. (2018)	531			1.18 (0.64 to 2.20)		7
Maternal weight	Gestational weight gain	Bilder et al. (2013)	392		**1.10 (1.03 to 1.17)**			8
	Insufficient GWG	Gardner et al. (2015)	605		1.12 (0.68 to 1.84)			8
	Excessive GWG	Gardner et al. (2015)	605		1.48 (0.93 to 2.38)			8
	Prepregnancy BMI	Gardner et al. (2015)	605		0.99 (0.95 to 1.03)			8
		Bilder et al. (2013)	392		0.93 (0.84 to 1.0)			8
Parity^m^		Piven et al. (1993)	39				***χ*^2^:4.7, *p* = .003**	6
Stress during pregnancy		Oerlemans et al. (2016)	152		**2.19 (1.16 to 4.13)**			7
		Grossi et al. (2018)	35		1.13 (0.85 to 1.23)			6
Composite scores of prenatal complications		Grossi et al. (2018)	35		1.81 (0.70 to 4.5)			6
		Deykin and MacMahon (1980)	145			**1.5 (1.1–2.0)**		6
		Abd Elhameed et al. (2011)	14				***χ*^2^, *p* = .0001**	5
		Bryson et al. (1988)	17				***F* (3,74) = 3.57, *p* < .02**	7
		Finegan and Quarrington (1979)	23				***χ*^2^ = 4.17 (1df), *p* < .05**	4
		Hadjkacem et al. (2016)	50				*p* = .13	6
		Lord et al. (1991)	46				*F* (1, 92) = 5.27, *p* < .02	7
		Piven et al. (1993)	39				*F* (1,38) = 0.45, *p* = ns	6
		Rutt and Offord (1971)	33				*χ*^2^ :ns	6

Abbreviations: *N* = number of exposed twins/sibling or number of twin/sibling cases, depending on cohort or case/control study, SSRI = selective serotonin reuptake inhibitor, PCB = polychlorinated biphenyl, PVC = polyvinyl chloride, GWG = gestational weight gain, ns = nonsignificant at *p* = .05 level.

a HR 0.81 (0.57 to 1.14) for SSRI.

b Transformed by us from logistic regression betas.

c Asthma without medications as reference.

d Multivitamin, iron, and folic acid.

e All births in Sweden between 1987 and 2007, with in total 368,549 third-born siblings.

f All births in Sweden between 1973 and 2001, including offspring from 1,408,669 distinct fathers and 1,404,484 distinct mothers.

g Together with maternal age<35.

h Affected versus unaffected status remained significant in the regression model containing birth order as a predictor (*F*1,164 = 12.2; *p* < .001).

i Piven et al., 1993 reports without statistics that autistic subjects were more commonly first or fourth born.

j All births in Sweden between 1973 and 2008.

k All births in Denmark between 1997 and 2013, in total differently exposed 8,156 siblings in analysis.

l Subjects had lower vitamin-D level (mean = 24.0 nM, *SD* = 19.6, *n* = 58) than in their siblings (mean = 31.9 nM, *SD* = 27.7, *n* = 5).

m Optimal one or two as reference.

#### Perinatal and neonatal exposure

Out of 19 perinatal and neonatal exposures, 17 were investigated in more than one study (Table 3). Twelve case control studies investigated composite scores of complications occurring during the neonatal period or earlier, of which nine reported an association. Predominantly positive findings were found for hypoxia and respiratory stress. Hypoxia was measured in two case control studies, of twins (*N* = 274) and siblings (*N* = 941), respectively, both of which showed a significant association with ASD with an *OR* (95% CI) of 1.71 (1.08–2.71) and 1.81 (1.21–2.69) (Froehlich-Santino et al., 2014; Glasson et al., 2004). Similarly, three out of four case control studies on respiratory distress found an association, both using twins (*N* = 274) (Froehlich-Santino et al., 2014) and using siblings (*N* = 1,125) (Glasson et al., 2004; Hadjkacem et al., 2016; Piven et al., 1993). Small effect sizes with *OR* (95% CI) between 1.64 (1.15–2.34) and 2.11 (1.27–3.51) were reported in all studies but one, where the confidence interval was wide, (Hadjkacem et al., 2016). Mixed findings were reported for preterm birth (*k* = 5 studies) with one large population-based sibling cohort study reporting an HR of 3.2 (95%CI, 2.6–4.0), labor induction (*k* = 4), jaundice (*k* = 4), and low Apgar scores (*k* = 3). No statistically significant within-pair associations were reported regarding elective (*k* = 6) and emergency (*k* = 3) cesarean section, general anesthesia during labor (*k* = 3), breech presentation (*k* = 3), gestation more than 42 weeks (*k* = 2), difficult labor (*k* = 2), umbilical cord around neck (*k* = 2), and resuscitation (*k* = 2). All these studies reported small effect sizes, except for one small sibling case control study on difficult labor reporting a medium effect size (Hadjkacem et al., 2016). Single studies found associations of ASD with incubation and neonatal respiratory infection.

**Table 3.** Environmental factors, perinatal, neonatal, infancy, and childhood – autism spectrum disorder (ASD)

Environmental factor		Author (year)	*N*	HR (95% CI)	*OR* (95% CI)	RR (95%CI)	Other	NOS
***Perinatal and neonatal***								
Gestational age	Preterm birth	D'Onofrio et al., (2013a)	**–**^a^	**3.2 (2.6 to 4.0)**				8
		Grossi et al. (2018)	35		1.96 (0.5 to 7.6)			6
		Oerlemans et al. (2016)	152			ns		7
		Mason-Brothers et al. (1990)	233				*χ*^2^: ns	7
		Piven et al. (1993)	39				*χ*^2^: ns	6
	>42 weeks gestation	Lord et al. (1991)	46				*F* (1, 92) = 3.03, *p* < .08	7
		Mason-Brothers et al. (1990)	233				*χ*^2^: ns	7
Mode of delivery	Elective cesarean	Grossi et al. (2018)	35		2.75 (0.89 to 8.43)			6
		Glasson et al. (2004)	465		1.13 (0.79 to 1.63)			6
		Curran et al. (2015)	2,555		0.89 (0.76 to 1.04)			8
		Chien et al. (2018)	323		0.12 (0.01 to 1.20)			6
		Creagh et al. (2015)	262				*χ*^2^: *p* = .1113	3
		Mason-Brothers et al. (1990)	233				*χ*^2^: ns	7
	Emergency cesarean	Chien et al. (2018)	323		1.35 (0.79 to 2.32)			6
		Glasson et al. (2004)	465		1.19 (0.79 to 1.79)			6
		Curran et al. (2015)	2,555		0.97 (0.85 to 1.11)			8
Labor induction		Oberg et al. (2016)	–^b^	0.99 (0.88–1.10)				8
		Glasson et al. (2004)	465		**1.40 (1.03 to 1.90)**			6
		Chien et al. (2018)	323		1.08 (0.68 to 1.70)			6
		Mason-Brothers et al. (1990)	233				*χ*^2^: ns	7
Difficult labor		Hadjkacem et al. (2016)	50		3.6 (0.8 to 16)			6
		Chien et al. (2018)	323		2.43 (0.74 to 7.89)			6
General anesthesia during labor		Glasson et al. (2004)	465		1.09 (0.54 to 2.20)			6
		Creagh et al. (2015)	262				*χ*^2^: *p* = .7659	3
		Mason-Brothers et al. (1990)	233				*χ*^2^: ns	7
Breech presentation		Glasson et al. (2004)	465		1.40 (0.82 to 2.39)			6
		Mason-Brothers et al. (1990)	233				*χ*^2^: ns	7
		Piven et al. (1993)	39				*χ*^2^: ns	6
Umbilical cord around neck		Glasson et al. (2004)	465		1.19 (0.71 to 1.97)			6
		Mason-Brothers et al. (1990)	233				*χ*^2^: ns	7
Low Apgar		Glasson et al. (2004)	465		**1.64 (1.02 to 2.65)**			6
		Mason-Brothers et al. (1990)	233				*χ*^2^: ns	7
		Piven et al. (1993)	39				*χ*^2^: ns	6
Respiratory distress		Hadjkacem et al. (2016)	50		**5.2 (1.2 to 21.6)**			6
		Froehlich-Santino et al. (2014)	137		**2.11 (1.27 to 3.51)**^c^			8
		Glasson et al. (2004)	465		**1.64 (1.15 to 2.34)**			6
		Piven et al. (1993)	39				*χ*^2^: ns	6
Hypoxia		Glasson et al. (2004)	465		**1.81 (1.21 to 2.69)**			6
		Froehlich-Santino et al. (2014)	137		**1.71 (1.08 to 2.71)**^c^			8
Resuscitation		Chien et al. (2018)	323		1.65 (0.75 to 3.61)			6
		Glasson et al. (2004)	465		1.22 (0.93 to 1.59)			6
Composite score of perinatal complications		Oerlemans et al. (2016)	152		**1.70 (1.04 to 2.79)**			7
		Grossi et al. (2018)	35		1.94 (0.7 to 5.5)			6
		Abd Elhameed et al. (2011)	14				***χ*^2^: *p* = .0001**	5
		Hadjkacem et al. (2016)	50				***p* = .003**	6
		Rutt and Offord (1971)	33				***p* < .01**	6
		Bryson et al. (1988)	17				*F* (3,74) = 0.71, *p* > .05	7
		Finegan and Quarrington (1979)	23				*χ*^2^: ns	4
		Piven et al. (1993)	39				*F* (1,38) = 0.45, *p* = ns	6
Incubation		Chien et al. (2018)	323		**2.21 (1.23 to 3.95)**			6
Jaundice		Froehlich-Santino et al. (2014)	137		**1.69 (1.09 to 2.62)**^c^			8
		Chien et al. (2018)	323		1.42 (0.79 to 2.56)			6
		Mason-Brothers et al. (1990)	233				*χ*^2^: ns	7
		Piven et al. (1993)	39				*χ*^2^: ns	6
Neonatal respiratory infection		Hadjkacem et al. (2016)	50		**22.2 (2.5 to 191.03)**			6
Composite score of neonatal complications		Deykin and MacMahon (1980)	145			**2.0 (1.3 to 3.0)**		6
		Bryson et al. (1988)	17				***F* (3,74) = 4.02, *p* < .01**	7
		Finegan and Quarrington (1979)	23				***χ*^2^(1df) = 14.73 (1df) *p* < .001**	4
		Hadjkacem et al. (2016)	50				***p* = .042**	6
		Abd Elhameed et al. (2011)	14				*χ*^2^: *p* = .1	5
		Piven et al. (1993)	39				*F* (1,38) = 2.8, *p* = ns	6
		Ronald et al. (2010)	5,796				*r* = .00 *SE*: –.07	*7*
Composite score of pre-, peri- and neonatal complications		Bryson et al. (1988)	17				***F* (3,74) = 2.98, *p* < .04**	7
		Chien et al. (2018)	323				***F* = 7.41, *p* = .007**	6
		Finegan and Quarrington (1979)	23				***χ*^2^(1df) = 17.02, *p* < .001**	4
		Lord et al. (1991)	46				***F* (7, 91) = 2.43, *p* < .05**	7
		Rutt et al., (1971)	33				***p* < .01**	6
		Steffenburg et al., (1989)	22				***χ*^2^: *p* = .02**	8
		Zwaigenbaum et al. (2002)	78				***F* (1,104) = 8.1, *p* = .005**	7
		Deb et al. (1997)	30				ns	6
		Piven et al. (1993)	39				*F* (1,38) = 0.8, *p* = ns	6
***Infancy and childhood***								
Nursing	Not breastfed	Burd et al. (1988)	50				*χ*^2^(1df) = 0.22, *p* > .05	4
	Early introduction of top feeds	Manohar et al. (2018)	30		**6 (1.33 to 55.19)**			5
	Fatty acid deficiency symptoms	Brown et al. (2014)	19		**2.77 (1.28 to 5.99)**			3
	Not breastfed 1st hour	Brown et al. (2014)	19		**3.84 (1.12 to 14.28)**			3
	Place of birth	Brown et al. (2014)	19				*χ*^2^(1df) = 0.40, *p* = .620	3
	Maternal fish consumption	Brown et al. (2014)	19				*χ*^2^(2df) = 2.13, *p* = .343	3
	Exclusively breastfed	Brown et al. (2014)	19				*χ*^2^(2df) = 0.85, *P* = .653	3
	Virus infection during nursing	Isaksson et al. (2017)	206		6.75 (0.96 to 47.63)^d^			3
	Maternal antibiotics d. nursing	Isaksson et al. (2017)	206		0.52 (0.09 to 3.08)^d^			3
Maternal infection or antibiotics during pregnancy and nursing		Isaksson et al. (2017)	206		7.61 (0.96 to 60.46)^d^			3
Medication during childhood	Early antibiotic exposure	Hamad et al. (2018)	57,063	1.03 (0.86 to 1.23)				7
		Grossi et al. (2018)	35		2.03 (0.40 to 9.1)			6
Dysregulation 1st year		Willfors et al. (2017)	100				**β = 31.75, *p* = .03**	8
Medical events first 5 years		Willfors et al. (2017)	100				**β = 78.18, *p* = .002**	8
Childhood recurrent infections^e^		Mason-Brothers et al. (1993)	233		**2.03 (1.10 to 3.73)**			6

Significant associations in **bold**.

Abbreviation: *N* = number of exposed twins/sibling or number of twin/sibling cases, depending on cohort or case/control study, ns = nonsignificant at *p* = 0.05 level.

a All births in Sweden between 1973 and 2008, in total 2,665,666 siblings in analyses.

b All births in Sweden between 1992 and 2005, in total 694,612 in sibling analysis.

c Violating the assumption of independent data in analysis.

d Transformed by us from logistic regression betas.

e Mainly upper respiratory tract infections and otitis media.

#### Infancy and childhood exposure

Nine types of exposures in infancy and childhood were investigated (Table 3). Breastfeeding (*k* = 3 studies) and early exposure to antibiotics (*k* = 2) were the only factors included in more than one study. The studies on breastfeeding were of small sizes, assessed as having high risk of bias and showed mixed results with wide confidence intervals (Brown et al., 2014; Burd et al., 1988; Manohar et al., 2018). No statistically significant within-pair association was observed for early exposure to antibiotics with both sibling studies reporting small effect sizes (Grossi et al., 2018; Hamad et al., 2018). Single studies reported significant associations with recurrent infections in childhood, dysregulation during first year of life, and medical events the first 5 years of childhood.

### ADHD

#### Study characteristics

A total of 69 studies (53 cohort studies and 16 case control studies) on ADHD were included (see Tables 4 and 5 for full list of references). A categorical definition of ADHD was used in 30 studies, while 36 studies used dimensional outcomes and three studies applied both (Altink et al., 2008; Chatterji et al., 2014; Eilertsen et al., 2017). The studies were published between 1987 and 2019. Similar to ASD, the amount of publications increased considerably during the last decade. The studies originated mainly from Scandinavia (*k* = 36) and North America (*k* = 19). A twin design was used in 13 of the studies, while the remaining 57 studies used siblings as controls. The number of cases in the case control designs ranged from 16 to 3,447, with a median of 233.5. In the cohort studies, the number of analyzed siblings or twins ranged from 28 to 2 665,666, with a median of 12,674. Prospectively collected data were used in 46 cohort studies, and in four of the case control studies. Regarding age at diagnosis, all but two of the cohort studies on ADHD diagnosis lacked information for the sibling subsamples. As for the ASD studies, when considering the general methodology of the whole samples, the risk of misclassification bias due to age of diagnosis was deemed low. Of the 39 studies using a dimensional outcome, 11 were performed on participants aged five years or younger. Several case control studies included a larger proportion of males among the cases than among the controls, and the sex distribution was often insufficiently reported. The NOS quality scores of the studies ranged between 3 to 9, with low scores more frequently seen in the case control studies. Common reasons for reduced scores were ascertainment of exposure and definition of controls. See Tables 4 and 5.

**Table 4.** Study characteristics – attention-deficit/hyperactivity disorder (ADHD), categorical (diagnosis)

COHORT STUDIES		Exposed population of twins/siblings				Unexposed population of twins/siblings					
Author (year)	Study design	Outcome/-s	Data source	*N* (% ♀)	Age *M* (SD); range	Comparator	*N* (% ♀)	Age *M* (SD); range	Country	Recruitment	NOS-score
Aagaard et al. (2018)	Pop. based, pro.	ASD, ADHD	Registry data	–^a^		Sibling	–		Denmark	Database linking	8
Axelsson et al. (2019)	Pop. based, pro.	ADHD	Registry data	–^b^		Sibling	–		Denmark	Database linking	7
Chang et al. (2014)	Pop. based, pro.	ADHD	Registry data	–^c^		Sibling	–		Sweden	Database linking	8
Chen et al. (2014)	Pop. based, pro.	ADHD	Registry data	–^d^		Sibling	–		Sweden	Database linking	8
Class et al. (2014)	Pop. based, pro.	ADHD, AST	Registry data	–^e^		Sibling	–		Sweden	Database linking^e^	8
Class et al. (2018)	Pop. based, pro., quasi-exp.	ASD, ADHD	Registry data	346,739 (–)		Sibling	1,050,271 (–)		Sweden	Database linking^f^	8
Curran et al. (2016)	Pop. based, pro.	ADHD	Registry data	6,976 (–)		Sibling	10,406 (–)		Sweden	Database linking^g^	8
D'Onofrio et al., (2013a)	Pop. based, pro., quasi-exp.	ADHD, AST	Registry data	–^h^		Sibling	–		Sweden	Database linking	8
D'Onofrio et al., (2014b)	Pop. based, pro.	ADHD, AST	Registry data	–^i^		Sibling	–		Sweden	Database linking	8
Eilertsen et al. (2017)	Pop. based, pro.	ADHD, *ADHD-sympt.*	Parental quest., registry data	–^j^		Sibling	–		Norway	Population based survey	6
Ginsberg et al. (2019)	Pop. based, pro., quasi-exp.	ADHD	Registry data	8,557 (–)		Sibling	8,557 (–)		Sweden	Database linking^k^	8
Hvolgaard Mikkelsen, Olsen, Bech, and Obel (2017)	Pop. based, pro.	ADHD	Registry data	6,436 (–)		Sibling	6,436 (–)		Denmark	Database linking^l^	8
Larsson, Sariaslan, Långström, D'Onofrio, and Lichtenstein (2014)	Pop. based, pro, quasi-exp.	ADHD	Registry data	–^m^		Sibling	–		Sweden	Database linking	8
Laugesen, Byrjalsen, Froslev, Olsen, and Sørensen (2017)	Pop. based, pro.	ADHD	Registry data	–^n^		Sibling	–		Denmark	Database linking	7
Laugesen, Olsen, Telen Andersen, Froslev, and Sørensen (2013)	Pop. based, pro.	ADHD	Registry data	348 (–)		Sibling	519 (–)		Denmark	Database linking^o^	7
Man et al. (2017)	Pop. based, pro.	ADHD	Registry data	–^p^		Sibling	–		Hong Kong	Database linking	7
Musser et al. (2017)	Pop. based, pro., quasi-exp.	ADHD	Registry data	–^q^	–; 5–12	Sibling	–	–; 5–12	USA	Database linking	8
Obel et al. (2011)	Pop. based, retro.	ADHD	Registry data	–^r^		Sibling	–		Finland	Database linking^r^	7
Obel et al. (2016)	Pop. based, retro.	ADHD	Registry data	–^s^		Sibling	–		Denmark	Database linking	7
Pettersson et al. (2019)	Pop. based, pro.	ASD, ADHD	Registry data	546,894 (49)	27.2 (15.1–40.9)	Sibling	546,894 (49)	27.2 (15.1–40.9)	Sweden	Database linking^t^	8
Skoglund, Chen, D'Onofrio, Lichtenstein, and Larsson (2014)	Pop. based, pro.	ADHD	Registry data	317,836 (–)		Sibling	47,603 (–)		Sweden	Database linking^u^	7
Sujan et al. (2017)	Pop. based, retro.	ASD, ADHD	Registry data	10,975 (–)^v^		Sibling	13,994 (–)^v^		Sweden	Database linking	7
Tillman et al. (2018)	Pop. based, pro.	ASD, ADHD, ID, CD	Registry data	6,844 (42)		Sibling	9,355 (48)		Sweden	Database linking^w^	8
Wiggs et al. (2017)	Pop. based, pro.	ADHD	Registry data	64,762 (–)		Sibling	64,762 (–)		Sweden	Database linking^x^	8

CASE CONTROL STUDIES		Study population of twins/siblings				Control population of twins/siblings					
Author (year)	Study design	Outcome/-s	Data source	*N* (% ♀)	Age *M* (SD); range	Comparator	*N* (% ♀)	Age *M* (SD); range	Country	Recruitment	
Altink et al. (2008)	Retro., G×E	ADHD, *ADHD-sympt.*	Clinical.	539 (20)	–; 5–17	Sibling	407 (60)	–; 5–17	8 countries^y^	From clinical practices	5
Ben Amor et al. (2005)	Retro.	ADHD	Clinical.	50 (10)	8.8 (1.7)	Sibling	50 (66)	10.1 (3.7)	Canada	From a clinical practice	5
Chatterji, Lahiri, and Kim (2014)	Pop. based, pro.	ADHD, *ADHD-sympt.,* ID	Parent report	732 (–)		Sibling	732 (–)		USA	Database linking and interview	5
Grizenko et al. (2012)	Retro., G×E	ADHD	Clinical.	71 (11)	9.0 (1.8)	Sibling	71 (49)	9.9 (2.5)	Canada	From clinical practices	7
Lehn et al. (2007)	Pop. based, pro.	ADHD	Quest., diagnostic interview	19 (53)	13.36 (1.54)	Twin	19 (53)	13.36 (1.54)	The Netherlands	Population based register	7
Mimouni-Bloch et al. (2013)	Retro.	ADHD	Clinical., quest.	56 (27)	10.36 (2.41)	Sibling	52 (52)	11.7 (3.50)	Israel	From a clinical practice	3
Myers et al. (2018)	Pro., deeply phenotyped	ASD, ADHD	Clinical.	64 (–)^z^		Twin	274 (–)		Sweden	From twin register and via advertisement	8
Oerlemans et al. (2016)	Retro.	ASD, ADHD	Clinical.	301 (25)	4 and 20 y	Sibling	175 (51)	4 and 20 y	The Netherlands	Through clinics and the Dutch Autism Association	7
Pearsall-Jones et al. (2008)	Retro.	ADHD, DCD	Parental quest.	16 (25)	13 (–)	MZ-twin	16 (25)	13 (–)	Australia	Voluntary twin register	4

Dimensional outcomes in *italic*. “–” and “ ” = not reported.

Abbreviations: *N* = number of subjects, *M* = mean, *SD* = standard, deviation, pop. based = population based, pro. = pro. exposure data, retro. = retrospective exposure data, quasi-exp. = quasi-experimental, G×E = Gene×Environment interaction, ASD = autism spectrum disorder, ADHD = attention-deficit/hyperactivity disorder, ID = intellectual disability, CD = communication disorders, DCD = developmental coordination disorder, ADHD-sympt. = ADHD-symptoms, MZ = monozygotic, DZ = dizygotic, Clinical. = clinical assessment, Quest. = questionnaire.

a All births in Demark between 1997 and 2013, in total differently exposed 12,467 siblings in analysis.

b All births in Denmark between 1997 and 2010, in total 117,529 exposed to cesarean delivery and 483,546 exposed to antibiotic treatment, with 6,821 informative families in analyses.

c All births in Sweden between 1988 and 2003, in total 988,625 (48.7% ♀) in analyses.

d All births in Sweden between 1992 and 2000, in total 272,790 siblings with 91.0% families contributing with two siblings.

e All births between in Sweden 1973 to 2008.

f All births in Sweden between 1987 and 2007, with 368,549 third-born siblings.

g All births in Sweden between 1990 and 2008.

h All births between 1973 to 2008, in total 2,665,666 siblings in analyses.

i All births between 1973 and 2001, including offspring from 1,408,669 distinct fathers and 1,404,484 distinct mothers.

j From the Norwegian Mother and Child Birth Cohort Study between 1999 and 2008 with 34,283 siblings from 94,907 mothers, covering 41% of all pregnancies in Norway.

k All births in Sweden between 1992 and 2002.

l All births in Denmark between 1991 and 2005.

m All births in Sweden between 1992 and 2000, with 430,344 siblings within 202,408 families.

n All birth in Denmark between 1996 and 2009, 44,660 siblings were discordant for exposure, with 2,246 children contributing informative to the estimates.

o All birth in Denmark between 1996 and 2009.

p All births in Hong Kong public hospitals between 2001 and 2009, with 53,616 siblings in analysis.

q From a large regional health care system in the upper Midwest of the United States of America, in total 1,958 siblings in analysis.

r All births in Finland between 1987 and 2001.

s All births in Denmark between 1991 and 2006, in total 684,042 siblings in analysis.

t All births in Sweden between 1973 and 1998.

u All births in Sweden between 1992 and 2000.

v All births in Sweden between 1996 and 2012. For SSRI: 9,063 exposed and 15,906 unexposed.

w All births in Sweden between 1973 and 2012.

x All births in Sweden between 1992 and 2005.

y Belgium, Germany, Ireland, Spain, Switzerland, the Netherlands, the United Kingdom, and Israel.

z Ages at examination ranged from 8 to 29 years (*M* = 16.2, *SD* = 5.2) for the whole sample. In total 70 MZ pairs and 49 DZ pairs.

**Table 5.** Study characteristics—attention-deficit/hyperactivity disorder (ADHD), dimensional (traits or symptoms)

COHORT STUDIES		Exposed population of twins/siblings				Unexposed population of twins/siblings					
Author (year)	Study design	Outcome/-s	Data source	*N* (% ♀)	Age *M* (SD); range	Comparator	*N* (% ♀)	Age *M* (SD); range	Country	Recruitment	NOS
Antshel and Waisbren (2003)	Pro.	*ADHD-sympt., IQ*	Clinical.	46 (56)	9.83 (–)	Sibling	18 (47)	10.67 (–)	USA	From a clinical practice	8
Asbury, Dunn, Pike, and Plomin (2003)	Pop. based, retro.	*Hyper.*	Parental quest.	2,353 (46)	4; 4-4	MZ-twin	2,353 (46)	4; 4-4	UK	Pop. based survey^a^	7
Asbury, Dunn, and Plomin (2006)	Pop. based, pro.	*Hyper.*	Parental quest.	2,581 (–)	4 and 7y	MZ-twin	2,581 (–)	4 and 7y	UK	Pop. based survey^a^	7
Ask et al. (2018)	Pop. based, pro.	*ADHD-sympt.*	Parental quest.	11,081 (–)^b^		Sibling	11,081 (–)^b^		Norway	Pop. based survey^c^	7
Berg, Trollfors, Hugosson, Fernell, and Svensson (2002)	Pop. based, pro.	*ADHD-sympt.*	Parental quest.	304 (42)	9.6; 6.5–14.3	Sibling	304 (49)	11.0; 6.1–15.3	Sweden	From clinical practices	6
Bergman et al. (1987)	Pro.	*Int., motor and lang. skills, ADHD-sympt.*	Clinical.	31 (35)	8.25; 2.75–17.25	Sibling	31 (52)	10.25; –	USA	From medical records	7
Brandlistuen, Ystrom, Nulman, Koren, and Nordeng (2013)	Pop. based, pro.	*ADHD-sympt*^d^, *motor skills*	Parental quest.	1,561 (–)^e^	3 (–)	Sibling	2,029 (–)^e^	3 (–)	Norway	Pop. based survey^c^	6
Brandlistuen et al. (2015)	Pop. based, pro.	*ADHD-sympt.*^d^	Parental quest.	112 (53)	1.5 and 3y	Sibling	14323 (54)	1.5 and 3y	Norway	Pop. based survey^c^	6
Brandlistuen et al. (2017)	Pop. based, pro.	*ADHD-sympt.*^d^	Parental quest.	–^f^		Sibling	–		Norway	Pop. based survey^c^	5
Eilertsen et al. (2017)	Pop. based, pro.	ADHD, *ADHD-sympt.*	Parental quest. and registry data	–^g^	5 (–)	Sibling	–	5 (–)	Norway	Pop. based survey^c^	6
Ellerbeck et al. (1998)	Pop. based, pro.	*IQ, ADHD-sympt.*^d^	Parental quest.	35 (–)	–	Sibling	35 (–)	10.2; 7–13.3	USA	Pop. based for all requiring surgery	7
Gjerde et al. (2017)	Pop. based, pro.	*ADHD-sympt.*^d^	Parental quest.	–^h^	1.5, 3 and 5y	Sibling	–	1.5, 3 and 5y	Norway	Pop. based survey^i^	5
Groen-Blokhuis, Middeldorp, van Beijsterveldt, and Boomsma (2011)	Pop. based, pro.	*ADHD-sympt.*	Parental quest.	MZ: 1,258 (–) DZ: 1,587 (–)^j^	3,7,10 and 12y	Twin	MZ: 1,258 (–) DZ: 1,587 (–)^j^	3,7,10 and 12y	The Netherlands	Pop. based register	6
Gustavson et al. (2017)	Pop. based, pro.	*ADHD-sympt.*	Parental quest. and registry data	530 (–)	5 (–)	Sibling	530 (–)	5 (–)	Norway	Pop. based survey^c^	5
Hultman et al. (2007)	Pop. based, pro.	*ADHD-sympt.*	Parental quest.	MZ: 471 (51) DZ: 371 (54) UZ: 130 (–)	9 and 14 y	Twin	MZ: 471 (51) DZ: 371 (54) UZ: 130 (–)	9 and 14 y	Sweden	Database linking and mailed questionnaires	8
Ichikawa, Fujiwara, and Kawachi (2018)	Pop. based, retro.	*ADHD-sympt.*	Parental quest.	550 (–)		Sibling	1050 (–)		Japan	Clustered random sampling in the Tokyo area	5
Jackson and Beaver (2015)	Pro., G×E	*ADHD-sympt.*	Patient quest.	–^k^		Sibling	–		USA	A multi-stage stratified sampling of schools	7
Knopik et al. (2016)	Pop. based, retro.	*ADHD-sympt.*	Teacher and parent quest.	173 (–)	–; 7–16	Sibling	173 (–)	–; 7–16	USA	Database linking and screening interviews^l^	6
Kung et al. (2018)	Pro.	*ADHD-sympt.*	Parental quest.	81 (53)	7.14 (–)	Sibling	55 (58)	7.46 (–)	UK	From clinical practices	7
Lim et al. (2018)	Pop. based, pro.	*ADHD-sympt.*	Patient, parent and teacher quest.	MZ: 3,499 (–) DZ: 6,698 (–)		Twin	MZ: 3,499 (–) DZ: 6,698 (–)		UK	Pop. based survey^m^	7
Marceau et al. (2017)	Pop. based, retro.	*ADHD-sympt.*	Diagnostic interview	173 (–)	–; 7–16	Sibling	173 (–)	–; 7–16	USA	From birth records and screening interviews^l^	6
Nulman et al. (2015)	Pro.	*ADHD-sympt., IQ*	Parental quest.	45 (51)	3.6 (0.84)	Sibling	45 (38)	5.7 (0.85)	Canada	From pregnancy counseling database	5
Oerbeck, Sundet, Kase, and Heyerdahl (2005)	Pop. based, pro.	*ADHD-sympt., IQ, lang., motor skills*	Clinical.	49 (59)	20.2 (0.9); 18.3–21.7	Sibling	41 (39)	21.4 (4.0); 12.3–30.0)	Norway	National screening program	7
Pettersson et al. (2015)	Pop. based, pro.	*ADHD-sympt.*	Parental interview	–^n^	9 and 12y	Twin	–	9 and 12y	Sweden	National twin register	9
Rosenqvist, Sjölander, Ystrom, Larsson, and Reichborn-Kjennerud (2018)	Pop. based, pro.	*ADHD-sympt.*	Parental quest.	3,270 (–)	5y	Sibling	2,477 (–)	5y	Norway	Pop. based survey^i^	5
Schultz et al. (2017)	Pro.	*Int., motor and language skills, ADHD-sympt.*	Parental quest.	14 (29)	4.8; 4.7–4.9	Twin	14 (–)	4.8; 4.7–4.9	USA	From a clinical practice	6
Sun et al. (2016)	Pro.	*ADHD-sympt., IQ, language skills*	Parental quest.	105 (10)	10.6 (2.0)	Sibling	105 (44)	10.9 (1.7)	USA	From a clinical practice	8
Tore et al. (2018)	Pro.	*ADHD-sympt.*^d^, *Att. prob.*	Parental quest.	MZ: 177 (43) DZ: 303 (50)	MZ: 3.1 (–) DZ: 2.9 (–)	Twin	MZ: 177 (43) DZ: 303 (50)	MZ: 3.1 (–) DZ: 2.9 (–)	UK	Online volunteering	6
Zachrisson, Dearing, Lekhal, and Toppelberg (2013)	Pop. based, pro.	*ADHD-sympt.*	Parental quest.	–^o^	1.5 and 3y	Sibling	–	1.5 and 3y	Norway	Pop. based survey^i^	5
Zheng et al. (2018)	Retro.	*ADHD-sympt.*^d^	Parental quest.	895 (50)	13.3; 8–18	Sibling	872 (48)	–	USA	From clinical practices	6

CASE CONTROL STUDIES		Study population of twins/siblings				Control population of twins/siblings					
Author (year)	Study design	Outcome/-s	Data source	*N* (% ♀)	Age *M* (SD); range	Comparator	*N* (% ♀)	Age *M* (SD); range	Country	Recruitment	
Altink et al. (2008)	Retro., G×E	ADHD, *ADHD-sympt.*	Clinical.	539 (20)	–; 5–17	Sibling	407 (60)	–; 5–17	8 countries^p^	From clinical practices	5
Bilenberg, Hougaard, Norgaard-Pedersen, Nordenbæk, and Olsen (2011)	Pop. based, pro.	*ADHD-sympt.*	Parental quest.	MZ: 27 (30) DZ: 139 (37)	MZ: 9.9 (3.2) DZ: 8.7 (3.1)	Twin	MZ: 27 (30) DZ: 139 (37)	MZ: 9.9 (3.2) DZ: 8.7 (3.1)	Denmark	Database linkage and mailing of questionnaires	7
Chatterji et al. (2014)	Pop. based, pro.	ADHD, *ADHD-sympt.*, ID	Parental quest.	732 (–)		Sibling	732 (–)		USA	Database linking and interview	5
D'Onofrio et al. (2007)	Pop. based, retro., quasi-exp.	*ADHD-sympt.*	Parental quest.	–^q^		Sibling	–		USA	Pop. based survey^r^	5
D'Onofrio et al. (2008)	Pop. based, retro.	*ADHD-sympt.*	Parental quest.	–^s^		Sibling	–		USA	Pop. based survey^r^	5
Ellingson, Goodnight, Van Hulle, Waldman, and D'Onofrio (2014)	Pop. based, retro.	*ADHD-sympt.*	Parental quest.	–^t^		Sibling	–		USA	Pop. based survey^r^	5
Mascheretti et al. (2017)	Retro., G×E	*ADHD-sympt.*	Parental quest.	–^u^		Sibling	–		Italy	From a clinical practice	4
McCusker, Armstrong, Mullen, Doherty, and Casey (2013)	Pro.	*ADHD-sympt.*	Parental quest.	31 (29)	7.7 (0.56)	Sibling	18 (50)	9.6 (1.8)	UK	From a clinical practice	6
Ramanathan, Balasubramanian, and Faraone (2017)	Pop. based, retro.	*ADHD-sympt.*^d^	Parental quest.	2,069 (50)	9.13 (2.99)	Sibling	2,069 (50)	9.13 (2.99)	USA	Pop. based survey^r^	7

Dimensional outcomes in *italic*. “–” and “ “ = not reported.

Abbreviations: *N* = number of subjects, *M* = mean, *SD* = standard, deviation, pop. based = population based, pro. = pro. exposure data, retro. = retrospective exposure data, quasi-exp. = quasi-experimental, G×E = Gene×Environment interaction, ASD = autism spectrum disorder, ADHD = attention-deficit/hyperactivity disorder, ID = intellectual disability, CD = communication disorders, DCD = developmental coordination disorder, ADHD-sympt. = ADHD-symptoms, Hyper. = hyperactivity traits, Int. = Intellectual, Lang. = language, IQ = Intelligent quotient, Att. prob. = Attention problems, MZ = monozygotic, DZ = dizygotic, UZ = unknown zygosity, Clinical. = Clinical assessment, Quest. = questionnaire.

a From the Twins Early Development Study (TEDS).

b Same-sex siblings.

c From the Norwegian Mother and Child Birth Cohort Study between 1999 and 2008, covering approximately 40% of all pregnancies in Norway.

d Externalizing behavior as proxy for ADHD-symptoms.

e All sibling pairs were same-sex.

f A total of 13,191 siblings.

g A total of 34,283 siblings (48% female).

h A total of 17,830 siblings in analyses.

i From the Norwegian Mother and Child Birth Cohort Study between 1999 and 2009, covering 41% of all pregnancies in Norway.

j Same-sex DZ twin pairs.

k 633 siblings in analysis.

l From the Missouri Mothers and Their Children study (MO-MATCH), birthyears 1998–2005.

m The Twins Early Development Study (TEDS), with twins born in England and Wales between 1994 and 1996.

n Parents of all Swedish 9- and 12-year-old twins born between 1992 and 2000 were interviewed. A total of 21,775 twins.

o A total of 17,910 siblings in analysis.

p Belgium, Germany, Ireland, Spain, Switzerland, the Netherlands, the United Kingdom and Israel.

q A total of 3,447 siblings from 1,258 mothers in analyses.

r From the National Longitudinal Survey of Youth 1979 and their children.

s A total of 1,752 siblings from 704 mothers in analyses.

t A total of 1,684 siblings in analyses.

u From a research project on developmental dyslexia with 238 probands and 230 siblings.

#### Prenatal exposure

The studies included 19 prenatal exposures, of which 10 were investigated in more than one study (Tables 6 and 7). Predominantly positive associations were observed for fetal growth/birth weight. For a categorical outcome, two large population-based sibling studies (Class et al., 2014; Pettersson et al., 2019) and a small twin study (*N* = 38) (Lehn et al., 2007) showed associations including a reported HR of 2.44 (95% CI, 1.99–2.97), an *OR* of 2.36 (95% CI, 2.27–2.43) and *t* (18) = −1.99, *p* (one-tailed) = .031, while a study with higher risk of bias reported no association (*N* = 1,464) (Chatterji et al., 2014). The same pattern was seen for a dimensional outcome, with two large population-based sibling studies (Jackson & Beaver, 2015; Lim et al., 2018) and two twin studies (*N* = 8,594) (Groen-Blokhuis et al., 2011; Hultman et al., 2007; Pettersson et al., 2015; Tore et al., 2018) reporting associations, while studies with higher risk of bias reported no associations or mixed results (*N* = 2,581) (Asbury et al., 2006; Mascheretti et al., 2017). Mixed results were seen for smoking (*k* = 11 studies) and alcohol use (*k* = 3) during pregnancy, parental age (*k* = 6) and maternal depression (*k* = 2). Smoking was frequently studied, and, interestingly, somewhat different patterns emerged depending of types of outcome. Predominantly, no statistically significant within-pair associations were seen regarding a diagnosis of ADHD, with three large population-based sibling cohort studies (Obel et al., 2011, 2016; Skoglund et al., 2014) and one sibling case control study (*N* = 476) (Oerlemans et al., 2016) showing no association, and with one sibling case control study of higher risk of bias reporting an association (*N* = 906) (Altink et al., 2008). For dimensional measures of ADHD-symptomatology the results were less clear, with one large population-based sibling cohort study (Gustavson et al., 2017) and two sibling case control studies (D'Onofrio et al., 2008; Ellingson et al., 2014), showing no association, with two studies of higher risk of bias reporting an association (Altink et al., 2008; Mascheretti et al., 2017) and with two of a lower risk of bias reporting mixed results (Knopik et al., 2016; Marceau et al., 2017). A population-based cohort study on siblings found an association between alcohol use during pregnancy and a dimensional but not categorical ADHD outcome (Eilertsen et al., 2017), while one population-based case control study (D'Onofrio et al., 2007) and one cohort study (Ichikawa et al., 2018), both on siblings, found no association using dimensional measures. For parental age, one sibling case control study found no association of parental age with a categorical outcome of ADHD (*N* = 476) (Oerlemans et al., 2016), one large population-based sibling cohort study found teenage birth to be protective, HR 0.81 (95%CI, 0.71–0.94), one did not, HR 1.28 (95%CI, 0.94–1.73) (Chang et al., 2014; Hvolgaard Mikkelsen, Olsen, Bech, & Obel, 2017), and one sibling case control study with higher risk of bias found an association with a small effect size between advanced maternal age and ADHD (*N* = 108) (Mimouni-Bloch et al., 2013). Furthermore, one population-based sibling cohort study found a strong association between advanced paternal age and ADHD diagnosis, HR 13.13 (95%CI, 6.85–25.16) (D'Onofrio et al., 2014b). Two sibling cohort studies with moderate risk of bias, including age of participants five years or younger, showed conflicting results regarding associations between maternal depression and dimensional outcomes of ADHD (Gjerde et al., 2017; Nulman et al., 2015). No statistically significant within-pair associations were reported for antidepressive medication during pregnancy (*k* = 3 categorical and 2 dimensional studies), maternal infection (*k* = 2 categorical and 1 dimensional study), stress or adverse family life events during pregnancy (*k* = 2 categorical and 1 dimensional studies), maternal weight (*k* = 2 categorical studies), and birth order (*k* = 2 categorical studies). All these studies reported small effect sizes, except for one small sibling case control study on stress during pregnancy reporting a medium effect size, with wide confidence interval, on ADHD diagnosis (Grizenko et al., 2012). An additional 10 environmental factors were investigated in single studies. These studies suggested associations of head circumference at birth and orofacial clefts with ADHD diagnosis, and paracetamol exposure and possibly history of miscarriage with ADHD-symptoms.

**Table 6.** Environmental factors—categorical attention-deficit/hyperactivity disorder (ADHD)-diagnosis

Environmental factor		Author (year)	N	HR (95% CI)	*OR* (95% CI)	Other	NOS
**Prenatal**							
Maternal medication	Antidepressant	Sujan et al. (2017)	10,975	0.99 (0.79 to 1.25)^a^			7
		Laugesen et al. (2013)	348	0.7 (0.4 to 1.4)			7
		Man et al. (2017)	–^b^	0.54 (0.17 to 1.74)			7
	Glucocorticoids	Laugesen et al. (2017)	–^c^	1.03 (0.87 to 1.20)			7
Smoking during pregnancy		Altink et al. (2008)	539			***χ*^2^ = 6.91, *p* = .009**^d^	5
		Obel et al. (2011)	–^e^	1.20 (0.97 to 1.49)			7
		Obel et al. (2016)	–^f^	1.07 (0.94 to 1.22)			7
	1–9 cig/day	Skoglund et al. (2014)	317,836	0.88 (0.73 to 1.06)			7
	≤10 cig/day	Skoglund et al. (2014)	317,836	0.84 (0.65 to 1.06)			7
		Oerlemans et al. (2016)	301		1.18	*p* > 0.05	7
Alcohol use during pregnancy		Eilertsen et al. (2017)	–^g^	0.97 (0.93 to 1.01)			6
Parental age	Advanced, both parents	Oerlemans et al. (2016)	301		1.20	*p* > 0.05	7
	Paternal age >45	D'Onofrio et al. (2014b)	–^h^	**13.13 (6.85 to 25.16)**			8
	Advanced maternal age	Mimouni-Bloch et al. (2013)	56		**1.10 (1.02 to 1.20)**		3
	Age <20 y	Chang et al. (2014)	–^i^	**0.81 (0.71 to 0.94)**			8
		Hvolgaard Mikkelsen, Olsen, Bech, and Obel (2017)	6,436	1.28 (0.94 to 1.73)			8
Birth order	First born	Oerlemans et al. (2016)	301		1.16 (0.99 to 1.35)		7
		Pearsall-Jones et al. (2008)	16			*χ*^2^ = ns	4
Interpregnancy interval	0–5 months	Class et al. (2018)	346,739	0.83 (0.64 to 1.07)			8
	6–11 months	Class et al. (2018)	346,739	0.92 (0.76 to 1.11)			8
	12–23 months	Class et al. (2018)	346,739	0.91 (0.76 to 1.07)			8
Prenatal testosterone levels	Higher 2D:4D ratio	Myers et al. (2018)	64			*r* = − .003 (− .017 to .012)	8
Fetal growth	Birth weight	Class et al. (2014)	–^j^	**2.44 (1.99 to 2.97)**			8
		Pettersson et al. (2019)	546,894		**2.36 (2.27 to 2.43)**		8
		Lehn et al. (2007)	19			***t* (18) = −1.99, p, one-tailed = 0.031**	7
		Chatterji et al. (2014)	732			*F* = −0.12, *p* > .1	5
Head circumference at birth		Aagaard et al. (2018)	–^k^	**0.91 (0.87 to 0.95)**			8
Malformations	Orofacial clefts	Tillman et al. (2018)	6,844	**2.19 (1.89 to 2.62)**^l^			8
Maternal weight	Prepregnancy overweight	Chen et al. (2014)	–^m^	0.98 (0.83 to 1.16)^n^			8
		Musser et al. (2017)	–^o^		0.92 (0.80 to 1.05)^l^		8
Maternal medical conditions	Maternal disease	Oerlemans et al. (2016)	301		1.07	*p* > 0.05	7
	Maternal infections^p^	Ginsberg et al. (2019)	8,557	1.03 (0.76 to 1.41)			8
		Oerlemans et al. (2016)	301		2.63	*p* > 0.05	7
Stress during pregnancy		Grizenko et al. (2012)	71		6.29 (1.45 to 27.26)		7
		Oerlemans et al. (2016)	301		1.55	*p* > 0.05	7
**Perinatal and neonatal**							
Gestational age	Preterm birth	D'Onofrio et al. (2013a)	**–**^q^	**2.3 (2.0 to 2.8)**			8
Mode of delivery	Non-VD	Pearsall-Jones et al. (2008)	16			*χ*^2^: ns	4
	Assisted VD	Curran et al. (2016)	6,976	1.04 (0.94 to 1.15)			8
	Elective CS	Curran et al. (2016)	6,976	1.05 (0.93 to 1.18)			8
		Axelsson et al. (2019)	–^r^	1.03 (0.91 to 1.16)			7
	Emergency CS	Curran et al. (2016)	6,976	**1.13 (1.01 to 1.26)**			8
		Axelsson et al. (2019)	–^r^	1.09 (0.97 to 1.24)			7
Labor induction		Wiggs et al. (2017)	64,762	0.99 (0.91–1.07)			
Obstetric complication		Oerlemans et al. (2016)	301		2.13 (1.05 to 4.33)		7
Neonatal oxygen perfusion		Pearsall-Jones et al. (2008)	16			*p* = .81	4
Pre-, peri-, and neonatal complications		Ben Amor et al. (2005)	50			***F*(4,196) = 3.67, *p* < .006**	5
**Infancy and childhood**							
Lack of breastfeeding at 3 months		Mimouni-Bloch et al. (2013)	56		**3.08 (1.46 to 6.50)**		3
Antibiotics during first 2 years	Penicillin	Axelsson et al. (2019)	–^r^	0.98 (0.90 to 1.07)			7
	Broader spectrum	Axelsson et al. (2019)	–^r^	0.99 (0.92 to 1.06)			7
Low family income	Quartile 1	Larsson et al. (2014)	–^s^	**1.37 (1.07 to 1.75)**			8
	Quartile 2	Larsson et al. (2014)	–^s^	**1.37 (1.12 to 1.68)**			8
	Quartile 3	Larsson et al. (2014)	–^s^	**1.23 (1.04 to 1.45)**			8
Parental divorce		Mimouni-Bloch et al. (2013)	56		**3.78 (1.0 to 15.20)**		3

Significant associations in **bold**.

Abbreviations: *N* = number of exposed twins/sibling or number of twin/sibling cases, depending on cohort or case/control study, VD = vaginal delivery, CS = cesarean section, ns = nonsignificant at *p* = .05 level.

a HR = 0.94 (0.73–1.22) for SSRI.

b All births in Hong Kong public hospitals between 2001 and 2009, with 53,616 siblings in analysis.

c All birth in Denmark between 1996 and 2009, 44,660 siblings were discordant for exposure, with 2,246 children contributing informative to the estimates.

d This association remained significant after stratifying by 7-repeat allele carriership.

e All births in Finland between 1987 and 2001.

f All births in Denmark between 1991 and 2006, in total 684,042 siblings in analysis.

g From the Norwegian Mother and Child Birth Cohort Study between 1999 and 2008 with 34,283 siblings from 94,907 mothers, covering 41% of all pregnancies in Norway.

h All births between 1973 and 2001, including offspring from 1,408,669 distinct fathers and 1,404,484 distinct mothers.

i All births in Sweden between 1988 and 2003, in total 988,625 (48.7% ♀) in analyses.

j All births between in Sweden 1973 to 2008.

k All births in Demark between 1997 and 2013, in total differently exposed 12,467 siblings in analysis.

l Transformed by us from logistic regression betas.

m All births in Sweden between 1992 and 2000, in total 272,790 siblings with 91.0% families contributing with two siblings.

n Obesity HR: 1.15 (0.85 to 1.56).

o From a large regional health care system in the upper Midwest of the United States of America, in total 1,958 siblings in analysis.

p Maternal infection requiring hospitalization during pregnancy.

q All births between 1973 to 2008, in total 2,665,666 siblings in analyses.

r All births in Denmark between 1997 and 2010, in total 117,529 exposed to cesarean delivery and 483,546 exposed to antibiotic treatment, with 6,821 informative families in analyses.

s All births in Sweden between 1992 and 2000, with 430,344 siblings within 202,408 families.

**Table 7.** Environmental factors—dimensional attention-deficit/hyperactivity disorder (ADHD) traits or symptoms.

Environmental factor		Author (year)	*N*	Signal of association	Comment	NOS
**Prenatal**						
Maternal medication	Antidepressant	Brandlistuen et al. (2015)	112	No	Effect of prenatal exposure to antidepressants was specific to anxiety, and not associated with emotional reactivity, somatic complaints, sleep problems, *attention problems* or aggression	6
		Nulman et al. (2015)	45	No	Exposed and unexposed siblings did not differ on Child Behavior Checklist internalizing, e*xternalizing,* and total scores	5
	Paracetamol	Brandlistuen et al. (2013)	1,561	Yes	Discordant siblings with exposure for more than 28 days had more *externalizing behavior* (β = 0.28, 95% CI 0.15–0.42), and higher *activity levels* (β = 0.24, 95% CI 0.11– 0.38)	6
	Ibuprofen	Brandlistuen et al. (2013)	1,561	No		6
	Benzodiazepine	Brandlistuen et al. (2017)	–^a^	No	No effects were observed for *externalizing behavior* problems in the sibling-matched models for short-term and long-term exposure, at child age 1.5 or 3 years	5
Smoking during pregnancy		Altink et al. (2008)	539	Yes	Exposed children had higher scores on all scales, but only with significant teacher-rated *total*, *hyperactive* and *inattentive* scales	5
		Mascheretti et al. (2017)	–^b^	Yes	Smoking during pregnancy was significantly correlated with *hyperactivity/ impulsivity*	4
		Knopik et al. (2016)	173	Possibly	Mainly no associations after control for familial confounding, except for the Conner's parent report *on hyperactive/impulsive* and, to a lesser extent, *total ADHD symptoms*	6
		Marceau et al. (2017)	173	Possibly	There were within-family effects of smoking during pregnancy on one instrument measuring *hyperactivity/impulsivity* and *total ADHD behaviors*, but not the other instrument or on *inattentiveness*. Used the same cohort as Knopik et al., 2016	6
		D'Onofrio et al. (2008)	–^c^	Possibly/No	Maternal smoking during pregnancy was associated with *ADHD-symptoms*, but the sibling comparison suggested a small association that was not statistically significant	5
		Ellingson et al. (2014)	–^d^	No	When accounted for familial confounding the association between smoking during pregnancy and *ADHD symptoms* attenuated	5
		Gustavson et al. (2017)	530	No	Sibling control analyses showed no association between maternal smoking in pregnancy and child *ADHD symptoms* among siblings discordant for maternal smoking	5
Alcohol use during pregnancy		Eilertsen et al. (2017)	–^e^	Yes	Sibling comparison analysis attenuated the estimated full sample association, but it remained greater than zero (β = 0.017, 95% CI 0.005–0.030)	6
		D'Onofrio et al. (2007)	–^f^	No	Alcohol use during pregnancy was associated to a*ttention/impulsivity problems*, but more exposed siblings did not have more problems than there less exposed siblings	5
		Ichikawa et al. (2018)	550	No	Regression analysis revealed no difference *in attention problems* between differently exposed siblings (β = −0.58, 95% CI −2.78–1.63)	5
Parental age		Mascheretti et al. (2017)	–^b^	No		4
Fetal growth	Birth weight, BW	Pettersson et al. (2015)	–^g^	Yes	Within MZ pairs, birth weight remained significantly related to total *ADHD symptoms* (β = −0.74, 95% CI −0.97 to −0.51), *inattention symptoms* (β = −0.50, 95% CI −0.66 to −0.34), and *hyperactivity-impulsivity symptoms* (β = −0.24, 95% CI −0.35 to −0.12).	9
		Hultman et al. (2007)	972	Yes	The lighter twin in BW-discordant pairs had on average 13% higher *ADHD symptom* score at age 8 to 9 years (*p* = .006) and 12% higher *ADHD score* at age 13 to 14 years (*p* = .018) compared with the heavier twin	8
		Jackson and Beaver (2015)	–^h^	Yes	Low birth weight siblings are at significantly greater risk of exhibiting *ADHD symptoms* during childhood relative to their normal birth weight siblings	7
		Lim et al. (2018)	10,197	Yes	Birth weight significantly predicted *ADHD symptoms* from early childhood to late adolescence across assessment waves and raters	7
		Groen-Blokhuis et al. (2011)	2,845	Yes	In BW discordant MZ, DZ, and unrelated (UR) pairs, the child with the lower BW scored higher on *hyperactivity* and *attention problems* than the child with the higher BW and within-pair differences were similar for MZ, DZ, and UR pairs	6
		Tore et al. (2018)	480	Yes	In MZ twins, statistically and clinically significant associations were found between intrapair birth weight difference and difference in *externalizing behavior* and *attention problems*	6
		Asbury et al. (2006)	2,581	Possibly	Significant correlation between birthweight and teacher rated *hyperactivity* at age 7 in MZ discordant twins	7
		Chatterji et al. (2014)	732	Possibly	Having slower fetal growth rate than sibling was associated with *hyperactivity* score among boys, but not among girls or in the total sample	5
		Mascheretti et al. (2017)	–^b^	No	On both *hyperactivity* and *inattentiveness*	4
	Birth weight×dopamine genes (G×E)	Jackson and Beaver (2015)	–^h^	Possibly	Greater genetic risk on three dopaminergic genes (DAT1, DRD2, and DRD4) relative to a sibling appears to exacerbate the link between sibling differences in birth weight and sibling differences in *ADHD symptomatology*	7
Miscarriages in history		Mascheretti et al. (2017)	–^b^	Possibly	Significantly associated with *inattentiveness* but not *hyperactivity/impulsivity*	4
Maternal infections	Transplacental-acquired antibodies	Bilenberg et al. (2011)	166	No	Pneumococcus Polysaccharide 14 (PnPs14) was present in the *ADHD high scoring* twin more often than in the lower scoring twin (*P* = 0.04). All 23 other antibodies were un-significant	7
Parental depression		Nulman et al. (2015)	45	Yes	Severity of maternal depression was a significant predictor of Child Behavior Checklist *externalizing* (β = 0.457, *p* = .003,), and total scores (β = 0.494, *p* = .001)	5
		Gjerde et al. (2017)	–^i^	No	After sibling comparison, only concurrent maternal depression 1.5 to 5 years postpartum was significantly associated with *externalizing problems*	5
Adverse family life events during pregnancy		Rosenqvist et al. (2018)	3,270	No	Comparing exposure-discordant siblings resulted in attenuated estimates that were no longer statistically significant	5
**Perinatal and neonatal**						
Gestational age		Ask et al. (2018)	11,081	Yes	In sibling comparisons, children born early preterm had a mean score that was 0.24 SD (95% CI 0.14–0.34) higher on *ADHD symptom* tests, 0.33 SD (95% CI 0.24–0.42) higher on *inattention* tests, and 0.23 SD (95% CI 0.14– 0.32) higher on *hyperactivity/impulsivity* tests	7
Congenital conditions	Heart surgery	McCusker et al. (2013)	31	Yes	Problems with *attention* were found in comparison with siblings. Teacher reports were consistent with parents, although problems were of a lower magnitude	6
	Hypothyroidism	Oerbeck et al. (2005)	49	Yes	The exposed group attained significantly lower scores than sibling controls on *attention*, but not on *externalizing behaviors* nor *executive functioning*	7
	Adrenal hyperplasia	Kung et al. (2018)	81	No	No significant differences were found on *hyperactivity/inattention* between differently exposed siblings	7
	Neuroblastoma	Zheng et al. (2018)	895	Yes	Compared to siblings, neuroblastoma survivors had a higher prevalence of *attention deficits* (21% vs. 13%, *p* < .001)	6
	Exposed to elevated levels of phenylalanine	Antshel and Waisbren (2003)	46	Yes	Dose dependent exposure association with higher levels of phenylalanine being more detrimental on executive functions	8
**Infancy and childhood**						
Breastfeeding		Mascheretti et al. (2017)	–^b^	No	On both *hyperactivity* and *inattentiveness*	4
Meningitis		Berg et al. (2002)	304	Yes	The post-meningitic children had significantly more symptoms of *inattention*, *hyperactivity* and *impulsiveness* than their siblings	6
		Bergman et al. (1987)	31	No	All paired, multivariate, and repeated measures analysis of covariance with repeated covariate differences are nonsignificant	7
General anesthesia		Sun et al. (2016)	105	No	No statistically significant differences in mean scores were found between sibling pairs in *attention*, *executive function* and *behavior*	8
Surgery	d-Transposition of the great arteries	Ellerbeck et al. (1998)	35	No	No difference between siblings regarding *externalizing behaviors*	7
	Congenital heart disease surgery <6m	Schultz et al. (2017)	14	No	No differences between sibling pairs on parent reported *inattentiveness* or *hyperactivity/impulsiveness*	6
Parental depression		Gjerde et al. (2017)	–^i^	Possibly	After sibling comparison, only concurrent maternal depression 1.5 to 5 years postpartum was significantly associated with *externalizing problems*, but not 6 months postpartum	5
Parenting	Harsh parental discipline	Asbury et al. (2003)	2,353	Yes	Significant correlation between harsh parental discipline parent rated *hyperactivity* at age 4	7
		Asbury et al. (2006)	2,581	No	No significant correlation between harsh parental discipline at age 4 and teacher rated *hyperactivity* at age 7 in discordant MZ twins	7
	Negative parental feelings	Asbury et al. (2003)	2,353	Yes	Significant correlation between negative parental feelings parent rated *hyperactivity* at age 4	7
		Asbury et al. (2006)	2,581	No	No significant correlation between negative parental feelings at age 4 and teacher rated *hyperactivity* at age 7 in discordant MZ twins	7
	Instructive parent-child communication	Asbury et al. (2006)	2,581	Yes	Significant correlation between instructive parent–child communication at age 4 and teacher rated *hyperactivity* at age 7 in MZ discordant twins	7
	Informal parent-child communication	Asbury et al. (2006)	2,581	No	No significant correlation between Informal parent-child communication at age 4 and teacher rated *hyperactivity* at age 7 in MZ discordant twins	7
Time in childcare		Zachrisson et al. (2013)	–^j^	No	No relation between hours of homogenously high-quality child care and *externalizing behavior* problems was evident in sibling analyses	5
Parental education		Mascheretti et al. (2017)	–^b^	No	On both *hyperactivity* and *inattentiveness*	4
Transient income decline		Ramanathan et al. (2017)	2,069	Yes	Exposed children had significantly more *externalizing behavioral* problems than the non-exposed matched siblings	7

Abbreviations: *N* = number of exposed twins/sibling or number of twin/sibling cases, depending on cohort or case/control study, *SD* = standard deviation, 95%CI = 95% confidence interval, MZ = monozygotic.

a A total of 13,191 siblings.

b From a research project on developmental dyslexia with 238 probands and 230 siblings.

c A total of 1,752 siblings from 704 mothers in analyses.

d A total of 1,684 siblings in analyses.

e A total of 34,283 siblings (48% female).

f A total of 3,447 siblings from 1,258 mothers in analyses.

g Parents of all Swedish 9- and 12-year-old twins born between 1992 and 2000 were interviewed. A total of 21,775 twins.

h A total of 633 siblings in analysis.

i A total of 17,830 siblings in analyses.

j A total of 17,910 siblings in analysis.

#### Perinatal and neonatal exposure

Out of 11 perinatal and neonatal exposures, the only factors included in more than one study were mode of delivery and gestational age (Tables 6 and 7). Regarding gestational age, two large sibling cohort studies found associations; one with a categorical, HR 2.3 (95% CI, 2.0–2.8), and one with a dimensional outcome (Ask et al., 2018; D'Onofrio et al., 2013a). For mode of delivery all studies used a categorical outcome of ADHD. The results were mixed depending on the specific mode. One large population-based sibling cohort study showed an association, HR 1.13 (95% CI, 1.01–1.26), between emergency cesarean section, but not for elective cesarean or assisted vaginal delivery (Curran et al., 2016), while another large population-based sibling cohort study found no statistically significant within-pair association with either form of cesarean section (Axelsson et al., 2019). A small twin study with higher risk of bias also found no association (*N* = 32) (Pearsall-Jones et al., 2008). One single sibling study found associations of ADHD diagnosis with a composite score of pre-, peri-, and neonatal complications (Ben Amor et al., 2005). Regarding dimensional outcomes, three single studies found an association of attention problems with heart surgery, hypothyroidism, and neuroblastoma, respectively, and one study found an association between higher levels of phenylalanine exposure and executive functions.

#### Infancy and childhood exposure

Twelve different exposures in infancy and early childhood were investigated (Tables 6 and 7). Breastfeeding (*k* = 2 studies), low income or transient income decline (*k* = 2), meningitis (*k* = 2), and parenting (*k* = 2, based on the same cohort) were examined in more than one study. Positive associations were found for low income or transient income decline with one large cohort study regarding ADHD diagnosis, HR 1.37 (95% CI, 1.07–1.75) (Larsson et al., 2014), and one large cohort study regarding dimensionally assessed externalizing behaviors (Ramanathan et al., 2017). Regarding breastfeeding, one sibling case control study with high risk of bias reported an association between lack of breastfeeding at 3 months and a categorical outcome of ADHD (*N* = 108) (Mimouni-Bloch et al., 2013), while another sibling case control study showed no association to ADHD-symptoms (Mascheretti et al., 2017). Mixed results were found for meningitis and parenting, with all studies using dimensional outcomes. Single studies reported associations of ADHD diagnosis with parental divorce and maternal depression.

### Intellectual disability

#### Study characteristics

A total of 26 studies (21 cohort studies and five case control studies) on ID or a dimensional measure of IQ were identified (see Tables 8 and 9 for full list of references). A categorical definition was used in six studies (Chatterji et al., 2014; Heuvelman et al., 2018; Monset-Couchard et al., 2004; Steingass et al., 2013; Sussmann et al., 2009; Tillman et al., 2018), while 19 studies included analyses based on IQ scores, and one study used both (Petik et al., 2012). The studies were published between 1965 and 2019, and were predominantly from North America (*k* = 16). A twin design was used in eight of the studies. The number of cases in the case control studies ranged from 49 to 3,296. In the cohort studies, the number of analyzed siblings or twins ranged from 24 to 20,471, with a median of 73. The data were prospectively collected in all of the studies. Age at assessment were reported in all but six of the studies. When reported, the sex distribution was less skewed than in the ASD and ADHD studies. The NOS scores ranged from 5 to 9, with the most common reason for downgrading being representativeness of the exposed cohort. See Tables 8 and 9.

**Table 8.** Study characteristics—categorical (diagnosis) intellectual disability, communication disorders, developmental coordination disorder and TIC disorder

COHORT STUDIES		Exposed population of twins/siblings				Unexposed population of twins/siblings					
Author (year)	Study design	Outcome/-s	Data source	*N* (% ♀)	Age *M* (SD); range	Comparator	*N* (% ♀)	Age *M* (SD); range	Country	Recruitment	NOS-score
Brander et al. (2017)	Pop. based, pro.	Tic disorders	Registry data	–^a^		Siblings	–		Sweden	Database linking	7
Monset-Couchard, de Bethmann, and Relier (2004)	Pop. based, pro.	ID, *motor skills*	Clinical testing	36 (–)	–;3.25–17	Twin	36 (–)	–;3.25–17	France	All cases from intensive care unit	9
Petik, Czeizel, Banhidy, and Czeizel (2012)	Pro.	ID, *IQ*	Clinical testing	27 (–)		Sibling	46 (–)		Hungary	All cases in the Budapest area	7
Tillman et al. (2018)	Pop. based, pro.	ASD, ADHD, ID, CD	Registry data	6,884 (42)		Sibling	9,391 (48)		Sweden	Database linking^b^	8

CASE CONTROL STUDIES			Study population of twins/siblings			Control population of twins/siblings					
Author (year)	Study design	Outcome/-s	Data source	*N* (% ♀)	Age *M* (SD); range	Comparator	*N* (% ♀)	Age *M* (SD); range	Country	Recruitment	NOS-score
Chatterji et al. (2014)	Pop. based, pro.	ADHD, *ADHD-sympt*., ID	Parent report and quest.	732 (–)		Sibling	732 (–)		USA	Database linking and interview	5
Heuvelman et al. (2018)	Pop. based, pro., nested	ID	Registry data	3,296 (–)		Sibling	4,738 (–)		Sweden	Database linking^c^	7
Hyde, Aaronson, Randolph, Rickler, and Weinberger (1992)	Retro.	Tourette's syndrome	Clinical assessment	16 (75)	12.8 (1.4); 8–26	MZ-twins	16 (75)	12.8 (1.4); 8–26	USA	National news letters	7
Pearsall-Jones et al. (2008)	Retro.	ADHD, DCD	Parental quest.	16 (25)	13 (–)	MZ-twin	16 (25)	13 (–)	Australia	Voluntary twin register	4
Steingass, Taylor, Wilson-Costello, Minich, and Hack (2013)	Pro.	ID	Clinical testing	88 (53)	29.1 (6.0)	Twin	88 (53)	29.1 (6.0)	USA	From neonatal clinic	8
Sussmann, McIntosh, Lawrie, and Johnstone (2009)	Pro.	ID	Clinical testing	49 (49)	–; 12–23	Sibling	21 (52)	–; 12–23	UK	Through the Scottish public educational system	6

Dimensional outcomes in *italic*. “–” and “ ”  = not reported.

Abbreviations: *N* = number of subjects, *M* = mean, *SD* = standard, deviation, pop. based = population based, pro. = pro. exposure data, retro. = retrospective exposure data, quasi-exp. = quasi-experimental, G×E = Gene×Environment interaction, ASD = autism spectrum disorder, ADHD =  attention-deficit/hyperactivity disorder, ID = intellectual disability, CD = communication disorders, DCD = developmental coordination disorder, ADHD-sympt. = ADHD-symptoms, MZ = monozygotic, DZ = dizygotic, Clinical. = Clinical assessment, Quest. = questionnaire.

a All births in Sweden between 1973 and 2003, including 947,942 families with at least two differently exposed children, and 3,563 families including siblings discordant for tic disorders.

b All births in Sweden between 1973 and 2012.

c The Stockholm Youth Cohort of all individuals under 18 years of age who lived in Stockholm County for at least 1 year between 2001 and 2011.

**Table 9.** Study characteristics—*Dimensional (traits/symptoms)* intellectual disability, communication disorders, developmental coordination disorder and TIC disorder

COHORT STUDIES		Exposed population of twins/siblings				Unexposed population of twins/siblings					
Author (year)	Study design	Outcome/-s	Data source	*N* (% ♀)	Age *M* (SD); range	Comparator	*N* (% ♀)	Age *M* (SD); range	Country	Recruitment	NOS-score
Antshel and Waisbren (2003)	Pro.	*ADHD-traits, IQ*	Clinical testing	46 (56)	9.83 (–)	Sibling	18 (47)	10.67 (–)	USA	From a clinical practice	8
Beardslee, Wolff, Hurwitz, Parikh, and Shwachman (1982)	Pro.	*IQ*	Clinical testing	31 (–)	–; 5–22	Sibling	24 (–)	–; 5–22	USA	From a clinical practice	7
Bellido-González, Defior-Citoler, and Díaz-López (2007)	Pro.	*IQ*	Clinical testing	22 (55)	1,2,4y	Twin	22 (55)	1,2,4	Spain	Not defined	7
Bergman et al. (1987)	Pro.	*Int., motor, lang. skills, ADHD-sympt.*	Clinical assessment	31 (35)	8.25; 2.75–17.25	Sibling	31 (52)	10.25; –	USA	From medical records	7
Brandlistuen et al. (2013)	Pop. based, pro.	*ADHD-sympt., motor skills*	Parental quest.	1,561 (–)	3 (–)	Sibling	2,029 (–)	3 (–)	Norway	Pop. based survey^a^	6
Ellerbeck et al. (1998)	Pop. based, pro.	*IQ, Ext. beh.*	Clinical testing	35 (–)	–	Sibling	35 (–)	10.2; 7–13.3	USA	Pop. based for all requiring surgery	8
Gilman, Gardener, and Buka (2008)	Pro.	*IQ*	Clinical testing	2,064 (–)^b^	4 and 7y	Sibling	2,763 (–)	4 and 7y	USA	From the Collaborative Perinatal Project	7
Jannoun (1983)	Pro.	*IQ*	Clinical testing	122 (53)	10.8; 4.8–17.4	Sibling	67 (–)	10.1; 5.6–17	UK	All survivors from seven centers	8
Kilbride, Thorstad, and Daily (2004)	Pop. based, pro.	*IQ, lang., motor skills*	Clinical testing	25 (68)	3 and 5y	Sibling	25 (60)	3 and 5y	USA	All low birth weight infants from university hospital	8
Klein, Forbes, and Nader (1975)	Pro.	*IQ*	Clinical testing	50 (12)	9.2; 5–14	Sibling	44 (–)	10.1; 5–15	USA	All patients from three clinics	6
Lloyd-Still, Hurwitz, Wolff, and Shwachman (1974)	Pro.	*IQ, motor skills*	Clinical testing	41 (32)		Sibling	41 (56)		USA	From a clinical practice	7
Monset-Couchard et al. (2004)	Pop. based, pro.	ID, *motor skills*	Clinical assessment	36 (–)	–;3.25–17	Twin	36 (–)	–;3.25–17	France	All cases from intensive care unit	8
Nulman et al. (2015)	Pro.	*ADHD-traits, IQ*	Clinical testing	45 (51)	3.6 (0.84)	Sibling	45 (38)	5.7 (0.85)	Canada	From pregnancy counseling database	6
Oerbeck, Sundet, Kase, and Heyerdahl (2003)	Pop. based, pro.	*ADHD-sympt., IQ, lang. motor skills*	Clinical testing	49 (59)	20.2 (0.9); 18.3–21.7	Sibling	41 (39)	21.4 (4.0); 12.3–30.0)	Norway	From national screening program	8
Petik et al. (2012)	Pro.	ID, *IQ*	Clinical testing	27 (–)		Sibling	46 (–)		Hungary	All cases in the Budapest area	7
Raz et al. (1998)	Pro.	*IQ, motor skills*	Clinical testing	25 (48)	6.14 (1.47); 4–9	Twin	25 (48)	6.14 (1.47); 4–9	USA	From neonatal clinic	8
Raz, Shah, and Sander (1996)	Pro.	*Motor skills*	Clinical assessment	28 (50)	1.3 (0.76); 0.2–2.6	Twin	28 (50)	1.3 (0.76); 0.2–2.6	USA	From neonatal clinic	8
Rovet (1986)	Pro.	*IQ, lang. and motor skills*	Clinical testing	101 (71)	–; 1–9	Sibling	101 (–)	–	Canada	All identified cases	5
Schultz et al. (2017)	Pro.	*Int., motor and lang. skills, ADHD-sympt.*	Clinical assessment	14 (29)	4.8; 4.7–4.9	Twin	14 (–)	4.8; 4.7–4.9	USA	From a clinical practice	6
Schultz et al. (2005)	Pro.	*IQ*	Clinical testing	11 (27)	1 (–)	Twin	13 (–)	1 (–)	USA	From a clinical practice	8
Stokholm et al. (2018)	Pop. based, pro.	*IQ*	Clinical testing	20,471 (–)	18.8 (–)	Sibling	88,977 (–)	18.8 (–)	Denmark	Database linking^c^	8
Sun et al. (2016)	Pro.	*ADHD-sympt., IQ, lang. and motor skills*	Clinical testing	105 (10)	10.6 (2.0)	Sibling	105 (44)	10.9 (1.7)	USA	From a clinical practice	8
Ylitalo, Kero, and Erkkola (1988)	Pro.	*Motor skills*	Clinical assessment	MZ: 12(–) DZ: 10 (60)	9.4; 3–14	Twin	MZ: 12 (–) DZ: 10 (50)	9.4; 3–14	Finland	All twin births in Finland were contacted	9

CASE CONTROL STUDIES		Study population of twins/siblings				Control population of twins/siblings					
Author (year)	Study design	Outcome /-s	Data source	*N* (% ♀)	Age *M* (SD); range	Comparator	*N* (% ♀)	Age *M* (SD); range	Country	Recruitment	
Bishop (1997)	Pro.	*CD-sympt.*	Clinical assessment	19 (–)		MZ-twin	19 (–)		UK	Through advertisement	7
Churchill (1965)	Pro.	*IQ*	Clinical testing	50 (–)^d^	–; 5–15	Twin	50 (–)	–; 5–15	USA	From referral to public school psychologic clinic	8
Hyde et al. (1992)	Retro.	Tourette's syndrome	Clinical assessment	16 (75)	12.8 (1.4); 8–26	MZ-twins	16 (75)	12.8 (1.4); 8–26	USA	National news letters	7

Dimensional outcomes in *italic*. “–” and “ ”  = not reported.

Abbreviations: *N* = number of subjects, *M* = mean, *SD* = standard, deviation, pop. based = population based, pro. = pro. exposure data, retro. = retrospective exposure data, quasi-exp. = quasi-experimental, G×E = Gene×Environment interaction, ASD = autism spectrum disorder, ADHD =  attention-deficit/hyperactivity disorder, ID = intellectual disability, CD = communication disorders, DCD = developmental coordination disorder, IQ = Intelligent quotient, ADHD-sympt. = ADHD symptoms, Int. = Intellectual, Lang. = language, MZ = monozygotic, DZ = dizygotic, Quest. = questionnaire.

a From the Norwegian Mother and Child Birth Cohort Study between 1999 and 2008, covering approximately 40% of all pregnancies in Norway.

b There were 49.4% females in total sibling sample.

c From all draft board examinations in Denmark between 1995 and 2015, 3.4% female in total sample.

d There were 22 clinically identical sets of twins.

#### Prenatal exposure

Seven prenatal exposures were identified (Tables 10 and 11). Fetal growth was investigated in six studies, with consistent results that differed between studies of ID and studies of IQ. Two twin studies of high quality (*N* = 248) and a case control study (*N* = 1,464) with higher risk of bias showed no statistical within-pair association with a diagnosis of ID (Chatterji et al., 2014; Monset-Couchard et al., 2004; Steingass et al., 2013). For IQ, on the other hand, two twin studies (*N* = 144) and one sibling cohort study (*N* = 50) found associations (Bellido-González et al., 2007; Churchill, 1965; Kilbride et al., 2004). Suicide attempts with Tardyl during pregnancy was associated with ID and IQ in one sibling study, one large population-based sibling case control study suggested an increased risk for ID linked to nonoptimal gestational duration, and one large population-based sibling cohort study reported increased risk for ID linked to orofacial clefts.

**Table 10.** Environmental factors—categorical (diagnosis) for: intellectual disability, communication disorders, developmental coordination disorder and TIC disorder

Environmental factor		Author (year)	*N*	HR (95%CI)	*OR* (95% CI)	Other	NOS
INTELLECTUAL DISABILITY							
***Prenatal***							
Suicide attempt with Tardyl during pregnancy		Petik et al. (2012)	27			***χ*^2^(1df) = 79.3, *p* < .0001**	7
Fetal growth rate		Monset-Couchard et al. (2004)	36			*χ*^2^: *p* = .90	9
		Steingass et al. (2013)	88		6.00 (0.96 to 37.53)		8
		Chatterji et al. (2014)	732			β = −0.19, *p* < .05)	5
Gestational age^a^	21–31 weeks	Heuvelman et al. (2018)	3,296		**7.84 (4.55 to 13.50)**		7
	32–36 weeks	Heuvelman et al. (2018)	3,296		**1.79 (1.42 to 2.24)**		7
	42 weeks	Heuvelman et al. (2018)	3,296		1.21 (0.99 to 1.48)		7
	43–45 weeks	Heuvelman et al. (2018)	3,296		**2.07 (1.28 to 3.36)**		7
Malformations	Orofacial clefts	Tillman et al. (2018)	6,884	**2.73 (2.15 to 3.46)**			8
***Perinatal and neonatal***							
Breastfed on discharge		Sussmann et al. (2009)	49		**0.17 (0.05 to 0.66)**		6
Being second born		Steingass et al. (2013)	88		2.25 (0.45 to 11.15)		8
Abnormal ultrasound		Steingass et al. (2013)	88		2.85 (0.82 to 9.89)		8
Sepsis/NEC		Steingass et al. (2013)	88		1.64 (0.57 to 4.77)		8
COMMUNICATION DISORDER							
***Prenatal***							
Malformations	Orofacial clefts	Tillman et al. (2018)	6,884		**3.61 (2.57 to 5.07)**		8
DEVELOPMENTAL COORDINATION DISORDER							
Being first born		Pearsall-Jones et al. (2008)	16			ns	4
Mode of delivery		Pearsall-Jones et al. (2008)	16			ns	4
Neonatal oxygen perfusion		Pearsall-Jones et al. (2008)	16			*p* = .08	4
TIC DISORDER							
***Prenatal***							
Smoking during pregnancy	1–9 cigarettes	Brander et al. (2017)	–^b^	**0.72 (0.54 to 0.96)**			7
	⩾ 10 cigarettes	Brander et al. (2017)	–^b^	0.79 (0.55 to 1.15)			7
***Perinatal and neonatal***							
Birth weight		Brander et al. (2017)	–^b^	**1.46 (1.06 to 2.01)**			7
		Hyde et al. (1992)	16			**Not reported**^c^	7
	⩽2500 g	Brander et al. (2017)	–^b^	1.20 (0.8 to 1.80)			7
Preterm birth (<37 weeks)		Brander et al. (2017)	–^b^	1.20 (0.93 to 1.56)			7
Cesarean section		Brander et al. (2017)	–^b^	1.15 (0.91 to 1.46)			7
Low Apgar		Brander et al. (2017)	–^b^	ns			7
Labor presentation		Brander et al. (2017)	–^b^	1.45 (0.96 to 2.20)			7
Head circumference		Brander et al. (2017)	–^b^	1.03 (0.77 to 1.36)			7

Significant associations in **bold**.

Abbreviations: *N* = number of exposed twins/sibling or number of twin/sibling cases, depending on cohort or case/control study, NEC = necrotizing enterocolitis, ns = nonsignificant at *p* = .05 level.

a Gestational age 37–41 weeks as reference.

b All births in Sweden between 1973 and 2003, including 947,942 families with at least two differently exposed children, and 3,563 families including siblings discordant for tic disorders.

c No statistics reported, though Tourette's syndrome occurred in the lighter twin in all of the seven discordant twin pairs, with nine concordant pairs.

**Table 11.** Environmental factors—dimensional (symptoms and traits) for: intellectual disability, communication disorders, developmental coordination disorder and TIC disorder

Environmental factor		Author (year)	*N*	Signal of association	Comment	NOS
INTELLECTUAL DISABILITY, *IQ*						
**Prenatal**						
SSRI use during pregnancy		Nulman et al. (2015)	45	No	IQ (mean ± SD): Exposed sibling (103 ± 13) and unexposed (106 ± 12), *p* = .3	6
Suicide attempt with Tardyl		Petik et al. (2012)	27	Yes	IQ (mean ± SD): Exposed sibling (82.2 ± 20) and unexposed (100 ± 9.7), *p* = .004.	7
Smoking during pregnancy		Gilman et al. (2008)	2,064	No	Effects on outcome is either not present or not distinguishable from familial factors associated with maternal smoking	6
Fetal growth	Birth weight	Bellido-González et al. (2007)	22	Yes	The heavier twin generally had higher scores on IQ related measures	7
		Churchill (1965)	50	Yes	IQ (mean): Lighter twin (85.2) and heavier twin (80.9), *p* < .005	8
	*> 801-gram Preterm*	Kilbride et al. (2004)	25	Yes	IQ (mean ± SD): Extremely low birth weight sibling (85 ± 12) and heavier sibling (95 ± 11)	8
Maternal depression		Nulman et al. (2015)	45	No	β = 0.69; *p* = .14	6
**Perinatal and neonatal**						
Labor augmentation		Stokholm et al. (2018)	20,471	No	Clinically irrelevant yet significant difference only when considering parity	8
Exposed to elevated levels of phenylalanine		Antshel and Waisbren (2003)	46	No	IQ (mean ± SD): Exposed sibling (104.2 ± 10.7) and unexposed (102.1 ± 13.4)	8
Perinatal hypoxic risk		Raz et al. (1998)	25	No	*F* (l, 24) = 0.30, *p* = ns. Based upon moderate intrapair discrepancy of exposure.	8
**Infancy and childhood**						
Surgery	d-Transposition of the great arteries	Ellerbeck et al. (1998)	35	No	IQ-difference not significant between siblings	8
	Congenital heart disease surgery <6m	Schultz et al. (2017)	14	Yes	IQ (mean; range): Exposed twin (99; 49–130) and unexposed (109; 92–127), *p* = .02	6
Medical conditions	Congenital heart disease	Schultz et al. (2005)	11	Yes	IQ (mean ± SD): Exposed twin (85 ± 19.3) and unexposed (93.9 ± 16.0), *p* = .037	8
	Congenital hypothyroidism	Oerbeck et al. (2003)	49	Yes	Estimated group IQ difference: 7.96 (95% CI 3.1–12.8)	8
		Rovet (1986)	101	Yes	IQ (mean ± SD): Exposed sibling (109.7 ± 11.7) and unexposed (113.7 ± 15.6)	5
	Meningitis^a^	Bergman et al. (1987)	31	No	There were no significant differences.	7
Malnourishment		Lloyd-Still et al. (1974)	41	Yes	Significant difference among siblings aged 1.5 to 6 years but not among children aged 5 to 15 years.	7
		Klein et al. (1975)	50	Possibly	Mean group IQ difference; severely malnourished: 1.35, *p* = ns; moderately malnourished: 3.49, *p* = .01; lightly malnourished: 0.69, *p* = ns	6
		Beardslee et al. (1982)	31	No	IQ (mean ± SD): younger group exposed sibling (106.5 ± 12.96) and unexposed (110.4 ± 9.57); older group exposed sibling (101.9 ± 12.16) and unexposed (98.6 ± 6.64)	7
Age at prophylactic therapy of leukemia (ALL)		Jannoun (1983)	122	Yes	There were significant differences in IQ scores, more so for children receiving treatment at a younger age	8
Exposure to general anesthesia		Sun et al. (2016)	105	No	Mean IQ scores between exposed siblings (scores: full scale = 111; performance = 108; verbal = 111) and unexposed siblings (scores: full scale = 111; performance = 107; verbal = 111) were not statistically significantly different.	8
COMMUNICATION DISORDER, *language skills*						
**Perinatal and neonatal**						
Fetal growth	> 801-gram, preterm	Kilbride et al. (2004)	25	Possibly	Pure language measures were not significantly different, but two language-related tests were	8
Perinatal hazards		Bishop (1997)	19	No	MZ pairs with substantial differences in neonatal status did not differ in language outcome	7
**Infancy and childhood**						
Congenital heart disease surgery <6m		Schultz et al. (2017)	14	No	Preschool Language Scale-IV (mean; range): Exposed twin (107; 50–120) and unexposed (104; 82–131), *p* = .15	6
Medical conditions	Congenital hypothyroidism	Rovet (1986)	101	Yes	Exposed sibling scored lower on language tests	5
		Oerbeck et al. (2003)	49	Possibly	Significant sibling difference on one of three measures, but all show small absolute differences	8
	Meningitis	Bergman et al. (1987)	31	No	No significant differences	7
Exposure to general anesthesia		Sun et al. (2016)	105	No	No significant differences	8
DEVELOPMENTAL COORDINATION DISORDER, *motor skills*						
**Prenatal**						
Fetal growth	Birth weight	Ylitalo et al. (1988)	22	Possibly	Significant twin differences for fine motor skills and visuo-motor perception but not for gross motor skills	9
	> 801-gram Preterm	Kilbride et al. (2004)	25	Yes	Peabody Developmental Motor Scales mean sibling difference: 12.6 (95%Cl 4.3–20.9), *p* = .004	8
	SGA	Monset-Couchard et al. (2004)	36	Possibly	Former SGA twins tended to have motor deficiencies, *p* = .10	8
Maternal medication	Paracetamol	Brandlistuen et al. (2013)	1,561	Yes	Siblings exposed more than 28 days: β = 0.24, (95% CI 0.12–0.51); and less than 28 days: β = 0.10, (95% CI 0.02–0.19)	6
	Ibuprofen	Brandlistuen et al. (2013)	1,561	No	Ibuprofen exposure was not associated with neurodevelopmental outcomes	6
**Perinatal and neonatal**						
Perinatal hypoxic risk		Raz et al. (1998)	25	No	No significant difference between twins	8
		Raz et al. (1996)	28	No	No significant difference between twins	8
**Infancy and childhood**						
Congenital heart disease surgery <6 m		Schultz et al. (2017)	14	Yes	Significant twin differences for fine motor skills, but not visual motor integration (*p* = .06)	6
Medical conditions	Congenital hypothyroidism	Rovet (1986)	101	Yes	Significant findings on several measures	5
		Oerbeck et al. (2003)	49	Yes	Mean sibling differences of motor coordination, dominant hand: 11.06 (95%CI 14.6–8.5), *p*=<.001; and global motor proficiency: 12.10 (95% CI 9.3–15.0), *p*=<.001	8
	Meningitis	Bergman et al. (1987)	31	No	No significant sibling differences	7
Exposure to general anesthesia		Sun et al. (2016)	105	No	No significant sibling differences	8
Severely malnourished		Lloyd-Still et al. (1974)	41	No	Lincoln–Oseretsky (mean ± SD): Exposed sibling (16.5 ± 18.7) and unexposed (18.6 ± 15.6), *p* = ns	7
TIC DISORDER, *tic symptom severity*						
**Perinatal and neonatal**						
Fetal growth	Birth weight	Hyde et al. (1992)	16	Yes	Significant tic score difference between lighter and heavier twin	7

Abbreviations: *N* = number of exposed twins/sibling or number of twin/sibling cases, depending on cohort or case/control study, SSRI = selective serotonin uptake inhibitor, ALL = acute lymphatic leukemia, SGA = small for gestational age, *SD* = standard deviation, 95%CI = 95% confidence interval, MZ = monozygotic.

a Coxsackievirus, echovirus, or poliovirus.

#### Perinatal, neonatal, infancy, and childhood

Seven perinatal and neonatal exposures were investigated in single studies, of which not being breastfed on discharge from a special care unit was associated with ID (Tables 10 and 11).

Seven different exposures in infancy and childhood were investigated, of which three were investigated in more than one study, all with dimensional outcome of IQ. Two small sibling cohort studies (*N* = 292) found an association between congenital hypothyroidism and IQ (Oerbeck et al., 2003; Rovet, 1986), while three cohort studies showed mixed results regarding malnourishment (Beardslee et al., 1982; Klein et al., 1975; Lloyd-Still et al., 1974), and two cohort studies found mixed results for congenital heart disease surgery (Ellerbeck et al., 1998; Schultz et al., 2017).

### Developmental coordination disorder

#### Study characteristics

A total of 13 relevant studies (12 cohort studies and one case control study) were found for DCD (see Tables 8 and 9 for full list of references). A categorical definition was used in the case control study (Pearsall-Jones et al., 2008), while the cohort studies used dimensional outcomes of motor skills. The studies were published between 1974 and 2017, and were predominantly from North America (*k* = 8). A twin design was used in six of the studies. The case control study included 16 cases. In the cohort studies, the number of analyzed siblings or twins ranged from 28 to 3,590, with a median of 67. The data were prospectively collected in all the studies but one (Pearsall-Jones et al., 2008). Age at assessment was reported in all but two of the studies. When reported, no pattern of skewness in the sex distribution could be seen. The NOS scores ranged from 4 to 9, with the most common reason for downgrading being representativeness of the exposed cohort and adequacy of follow up of cohorts. See Tables 8 and 9.

#### Exposures

Twelve different exposures were identified for DCD or motor skills (see Tables 10 and 11 for full list of references). They were too few in order to be sorted according to chronology. Fetal growth was found associated with motor skills in two twin cohort studies (*N* = 116) and one sibling cohort study (*N* = 50) (Kilbride et al., 2004; Monset-Couchard et al., 2004; Ylitalo et al., 1988). Two small sibling cohort studies (*N* = 292) found an association between congenital hypothyroidism and motor skills (Oerbeck et al., 2003; Rovet, 1986). Perinatal hypoxic risk was not significantly associated to motor skills in two studies from the same twin cohort (*N* = 56) (Raz et al., 1996, 1998). One single population-based cohort showed an association with maternal paracetamol use during pregnancy (Brandlistuen et al., 2013), while one twin cohort study found an association with congenital heart disease surgery (Schultz et al., 2017), both using dimensional outcomes for motor skills. No other statistically significant within-pair associations were found.

### Communication disorder

#### Study characteristics

Eight eligible studies (seven cohort studies and one case control study) were identified for CD (see Tables 8 and 9 for full list of references). A categorical definition of communication disorder was used in one study (Tillman et al., 2018), while the remaining studies used dimensional outcome of language development. The studies were published between 1986 and 2018, and were predominantly from North America (*k* = 5). A twin design was used in two of the studies (Bishop, 1997; Schultz et al., 2017). There were 19 cases in the case control study (Bishop, 1997). In the cohort studies, the number of analyzed siblings or twins ranged from 28 to 16,275, with a median of 90. The data were prospectively collected in all the studies. Age at assessment was reported in all but two of the studies. When reported, no pattern of skewness in the sex distribution could be seen. The NOS scores ranged from five to eight, with the most common reason for downgrading being representativeness of the exposed cohort. See Tables 8 and 9.

#### Exposures

Seven different exposures were identified for CD or language skills (Tables 10 and 11). They were too few in order to be sorted according to chronology. Congenital hypothyroidism was linked to lower language skills in one sibling cohort study (*N* = 202) (Rovet, 1986), while another sibling cohort study showed an inconsistent and weak association (*N* = 90) (Oerbeck et al., 2003). One single large population-based cohort study suggested that orofacial clefts were associated with a categorical outcome of CD (Tillman et al., 2018). A possible association was also observed for fetal growth in preterm infants (Kilbride et al., 2004). No other statistically significant within-pair associations were found.

### Tic disorder

#### Study characteristics

Two studies were identified for TD (Tables 8 and 9). One of these was a large-scale Swedish cohort study based on registry data on siblings from the general population (Brander et al., 2017). The other was a retrospective case control study from the USA based on 16 MZ twins pairs (Hyde et al., 1992). Both studies had a NOS quality score of 7.

#### Exposures

Seven different exposures were identified for TD (Tables 10 and 11). They were too few in order to be sorted according to chronology. Birth weight, the only exposure studied in both studies, was associated with both a diagnosis of tic disorder, HR 1.46 (95% CI, 1.06–2.01), and with symptom severity. No other statistically significant within-pair associations were found.

### Specific learning disorder

No relevant studies were identified.

## Discussion

Twin and sibling studies can help disentangle genetic and environmental contributions to the pathways underlying NDDs. In the current systematic review, we found evidence, beyond familial confounding, that advanced paternal age, low birth weight, birth defects, and perinatal hypoxia and respiratory stress are consistently associated with a diagnosis of ASD. We also found evidence that low birth weight, gestational age and low family income or transient income decline during childhood are associated with ADHD, both categorically and dimensionally. There was some evidence for congenital hypothyroidism being associated with lower IQ, low motor skills, and possibly low language skills, but our confidence in these results is limited due to a higher risk of bias. While some studies suggested low birth weight to be associated with TD, tic symptom severity and lower IQ, there was no association with a diagnosis of ID.

Furthermore, we found *no evidence* that maternal uterine bleeding, maternal infection during pregnancy, season of birth, preeclampsia, prenatal testosterone level, urinary tract infection during pregnancy, gestational diabetes, prepregnancy body mass index, elective and emergency cesarean section, general anesthesia during labor, breech presentation, gestation longer than 42 weeks, difficult labor, umbilical cord around neck, resuscitation, and early exposure to antibiotics in childhood is associated with a diagnosis of ASD when familial confounding is taken into account; *no evidence* that antidepressive medication, maternal infection, and stress or adverse family life events during pregnancy are is associated with ADHD defined both categorically and dimensionally, or that maternal weight, smoking during pregnancy, and birth order are associated with a diagnosis of ADHD; and *no evidence* that perinatal hypoxic risk is associated with low motor skills, when controlled for familial confounding. It is important to keep in mind that, in general, absence of evidence is not the same thing as evidence that no association exists. This is especially true when the empirical evidence is scarce, due to too few studies and/or small sample sizes. Regarding the associations of maternal uterine bleeding, preeclampsia, gestational diabetes, pre-pregnancy body mass index, and elective and emergency cesarean section, with ASD, our results point in the direction of evidence of no association beyond familial confounding. The same is the case for the associations of antidepressive medication, maternal infection, maternal weight, and maternal smoking during pregnancy, with a diagnosis of ADHD. For the rest, the conclusion to be drawn is that no clear statement can be made.

The most extensively studied factors with conflicting findings are the associations between ASD and antidepressive medication during pregnancy, advanced maternal age, preterm birth, labor induction, and neonatal jaundice; and the associations between ADHD, both categorically and dimensionally, and alcohol use during pregnancy, and parental age. We found categorically cross-disorder associations of low birth weight (ASD, ADHD, and TD) and cross-dimensional associations for congenital hypothyroidism (lower IQ, low motor skills, and possibly low language skills).

With familial confounding being controlled for, the findings of the current review may point to several possible mechanisms underlying the associations between NDDs and environmental factors. For ASD, it has been shown that the father's age at conception correlates to the number of de novo mutations in their children (Kong et al., 2012). De novo mutations are in turn, among others, linked to ASD, thereby suggesting a possible genetic pathway (Neale et al., 2012; O'Roak et al., 2012; Sanders et al., 2012). For ADHD, a pathway has been hypothesized to explain the association between low family income or family income decline during early childhood and ADHD in offspring. These include evidence of a strong association between low SES and the prefrontal working memory system (Hackman, Farah, & Meaney, 2010), in turn described as a neuropsychological ADHD endophenotype (Castellanos & Tannock, 2002). As for the pathways underlying the association of restricted fetal growth with ASD, ADHD, and TD our cross-disorder finding is in line with a body of evidence linking fetal growth to these and several other psychiatric disorders. It has even been modelled that a general factor of psychopathology is linked to restricted fetal growth (Pettersson et al., 2019). Furthermore, birth weight differences have previously been linked to altered brain development (Walhovd et al., 2012), although with unknown mediating mechanisms. As for the link between smoking during pregnancy and ASD, Hultman, Sparén, and Cnattingius (2002) reported an *OR* of 1.4 (95% CI 1.1–1.8), but as shown in the most recent study by Kalkbrenner et al. (2020) this link is better explained by familial confounding, with the exposure of maternal smoking being associated with numerous social and social-class related factors and the possibility of genes affecting both exposure and outcome. This leads to the conclusion that factors considered to be environmental might actually not be strictly environmental. Therefore, it is a problem with referring to them as being ‘nongenetic’. This has been pointed out before (Plomin, DeFries, Knopik, & Neiderhiser, 2016), and we suggest for future studies to more comprehensively consider the genetic basis of ‘environmental’ factors in order to help us understand the etiology of NDDs.

As noted above, some apparent discrepancies were observed when comparing categorical or dimensional outcomes. First, contrary to the associations with ASD, ADHD, and TD, fetal growth did not show an association with ID diagnosis, but to the level of IQ. This points to the possibility of a different mechanism for a clinical diagnosis of ID compared to IQ level in the rest of the distribution. This is in line with the findings of Reichenberg et al. (2016), which suggested that the profound ID is a distinct entity from milder ID, with different genetic and environmental influences to milder ID. Second, regarding ADHD it is interesting to note that for smoking during pregnancy, despite no evidence of it being associated to an ADHD diagnosis aside from familial confounding, three of the four studies with a positive association looking at dimensional outcomes noted a link to hyperactivity/impulsivity, but not to inattentiveness (Table 7). This suggests that these traits might have different underlying mechanisms. Although these dimensions are differentially implicated in neuropsychological impairment (Willcutt et al., 2012), the underlying mechanisms are still unclear.

The latter shows that using dimensional outcomes compared to categorical ones differentiates different symptom dimensions within the same condition. It has previously been shown that social and nonsocial traits in ASD are genetically dissociable (Happé & Ronald, 2008), and that hyperactivity/impulsiveness and inattentiveness in ADHD have distinguishable underlying pathways (Castellanos, Sonuga-Barke, Milham, & Tannock, 2006; Kuntsi et al., 2014; Luo, Weibman, Halperin, & Li, 2019; Sonuga-Barke, 2005). This review cannot answer whether this holds true also for different environmental factors and ASD, since first, only two of the included studies on ASD used a dimensional measure, and second, those two only used a combined measure of total ASD severity, not separated on social and nonsocial traits. So, it remains unclear if social and nonsocial traits in ASD are environmentally dissociable. Although the value of a dimensional approach in NDD research is now undisputed, it is also important to keep in mind that dimensional data do not necessarily have clinical relevance, and there might be a qualitative shift in mechanisms along the symptomatic continuum.

Strikingly, while there is a wealth of studies on exposures in ASD and ADHD, and to some extent low IQ/ID, there is little research on other NDDs, including few to no studies on CD, except for specific learning disorders, despite these being common in the general population (Aschner & Costa, 2015; Bishop, 2010). This systematic review also points to the lack of geographic dispersion with most twin and sibling studies being conducted in North America and Scandinavia, highly developed areas of the world both with regards of environmental regulations and health care. It may, for example, not be possible to generalize our findings on obstetrical complications not being associated with ASD, to areas of the world with less developed obstetrical and neonatal care. Additional factors, not yet identified, could potentially be of relevance for NDDs in other parts of the world. The limited geographical spread points to the existence of a global research bias and divide for NDDs. According to Zhang et al. (2017), only 1.13% of the research productivity worldwide in the field of psychiatry originates from low and lower-middle income countries.

In this review of genetically informed studies, we found evidence, albeit with modest effect sizes, for several environmental factors potentially on the casual pathways for different NDDs, particularly ASD and ADHD. Other previously discussed factors were questioned, such as season of birth and a series of obstetrical- and pregnancy-related factors. Interestingly, a recent meta-analysis on birth by cesarean delivery by Zhang et al. (2019), came to a different result with an odds ratio [*OR*] of 1.33 (95% CI, 1.25–1.41) for ASD from 27 studies, and an *OR* of 1.17 (95% CI, 1.07–1.26) for ADHD from 13 studies. But, as the authors points out, the pattern of attenuation when performing sibling analyses suggested that the observed associations were likely due to familial confounding. Furthermore, our review found no evidence for antidepressive medication, maternal infection, and stress or adverse family life events during pregnancy being associated with ADHD, beyond familial confounding.

This systematic review integrates a number of methodological strengths. First, the most prominent strength is its size with 140 included articles. Second, it is the first systematic review in this growing field of research trying to rule out familial confounding in the search for causal environmental factors for NDDs. Third, the broad approach on NDD, rather than a single diagnosis only, of this review allowed to follow threads otherwise hard to follow regarding diagnostic specificity of particular findings. Fourth, we have included studies of both dimensional and categorical outcomes, addressing the possibility of different pathways for symptom/traits and diagnosis. Fifth, the diversity of the exposures covered reaching from pregnancy to early childhood, has allowed us to relate our findings to the timing of the exposure.

A potential limitation of the present review is the inclusion of early studies on environmental factors dating back decades. With recent study designs and statistical methods, potential environmental factors for ASD such as rubella infection during pregnancy and labor induction have been found to be confounded by familial factors, compared to results from earlier studies with higher risk of bias (Tables 2 and 3). This shows that with incautiously applied family designs we risk deeming risk factors as being free from familial confounding, when in fact, the full information that twins and siblings provide is not utilized to fully account for the familial confounding. This points to the need to utilize state-of-the-art methods for twin and family data. Therefore, it is time to reevaluate potential environmental factors from the past decades with a contemporary statistical approach. Another potential weakness of this review is that there are other ways to control for familial confounding than twin and sibling studies. Particularly, multi-generational population-based cohorts, not only including siblings, but also half-siblings and cousins, sometimes in a quasi-experimental design. Other ways to deal with familial confounding are, as previously discussed, based upon adoptions or in vitro fertilization (IVF) designs (D'Onofrio, 2014a). As explained by Harold et al. (2013), compared to family studies, these designs carry the advantage that further examination of associations between patterns of family interaction and child development is possible, as they also allow control for passive gene-environment interaction. As Loehlin (2016) highlights, the strength of adoption studies to estimate the effects of the prenatal and the postnatal environment, makes them well suited to investigate how familial confounding differentially applies to prenatal versus postnatal environmental risks. Furthermore, there is little control of comorbidity in the included studies. This limitation could not be addressed by this review, owing to a lack of reporting comorbidity in the primary studies examined. Future studies should be careful and comprehensive in mapping somatic and psychiatric comorbidity, which are frequent in NDD (Pan, Tammimies, & Bölte, 2019; Plana-Ripoll et al., 2019) and may have a significant impact on developmental mechanisms. Another potential limitation is discrepancy in age of diagnosis. Regarding ASD, most of the included cohort studies lacked specific information regarding the sibling subsamples. Despite this, the overall assessment of the included studies’ methodologies gives little room for a misclassification bias being present. Regarding studies on ADHD, it is important to bear in mind that some of the results rely on dimensional outcomes at a young age thereby introducing a risk of misclassification bias. Finally, while the results indicate that some previously suspected environmental factors are due to familial confounding, we once again caution against general conclusions that absence of evidence of an association equals evidence of absence.

### Conclusions and future directions

NDDs are common conditions, and although NDDs are highly heritable, environmental factors do contribute to their causal pathways and associated impairment. Studies on suspected environmental factors often suffer from the bias of familial confounding where exposures are in themselves heritable, with the risk of incorrectly connecting them to NDDs, possibly leading to waste of public resources, unnecessary worry, misleading advice, and eroded public trust.

The conclusions from this comprehensive systematic review of twin and sibling studies are as follows. First, we found evidence, beyond familial confounding, that:

- advanced paternal age, low birth weight, birth defects, and perinatal hypoxia and respiratory stress are consistently associated with a diagnosis of ASD, and;
- low birth weight, gestational age, and low family income or transient income decline during childhood are associated with ADHD, both categorically and dimensionally.

Second, our result points in the direction of evidence of *no* association beyond familial confounding regarding the associations of:

- maternal uterine bleeding, preeclampsia, gestational diabetes, pre-pregnancy body mass index, and elective and emergency cesarean section, with ASD, and;
- antidepressive medication, maternal infection, maternal weight and maternal smoking during pregnancy, with a diagnosis of ADHD.

Third, we found a substantial body of studies with conflicting findings regarding the associations of:

- antidepressive medication during pregnancy, advanced maternal age, preterm birth, labor induction, and neonatal jaundice with ASD, and;
- alcohol use during pregnancy, and parental age with ADHD, both categorically and dimensionally.

Fourth, there is a lack of geographic dispersion, with most twin and sibling studies being conducted in North America and Scandinavia. Additional factors, not yet identified, could potentially be of relevance for NDDs in other parts of the world. Finally, and perhaps most importantly, too few reliable conclusions can be drawn for conditions other than ASD and ADHD. This is unfortunate, given the considerable frequency of other NDDs, and points to a critical need of more genetically informed studies of good quality in the quest of the environmental causes of NDDs.

## Supplementary material

For supplementary material accompanying this paper visit https://doi.org/10.1017/S0954579420000620. click here to view supplementary material
