# Supplementary material for: Early environmental risk factors for neurodevelopmental disorders – a systematic review of twin and sibling studies
Source: Dev Psychopathol. 2021 Oct;33(4):1448–95. doi: 10.1017/S0954579420000620 (PMC8564717; doi:10.1017/S0954579420000620)
Supplement: Supplementary file 1 [file S0954579420000620sup.zip › S0954579420000620sup003.docx]

Documentation of search strategies

University Library search consultation group

Date: September-October 2017

Topic/research question: A Systematic Review of Environmental Factors for Neurodevelopmental Disorders in Twin and Family Studies

Name of researcher(s): Torkel Carlsson, Felix Molander, Mark Taylor, Ulf Jonsson & Sven Bölte, KIND

Librarian (s): Klas Moberg & Carl Gornitzki

Databases:

1. Medline, Epub Ahead of Print, In-Process & Other Non-Indexed Citations, Ovid MEDLINE(R) Daily and Ovid MEDLINE(R) (Ovid)
2. Psycinfo (Ovid)
3. Embase (Embase.com)
4. Web of Science Core Collection
5. Cochrane Library (Wiley)

Total number of hits:

- Before deduplication: 11,472
- After deduplication: 6,543

1. Medline

| Interface: Ovid  Date of Search: 5 October 2017  Number of hits: 2,680  Comment: In Ovid, two or more words are automatically searched as phrases; i.e. no quotation marks are needed | Field labels   - exp/ = exploded MeSH term - / = non exploded MeSH term - .ti,ab,kf. = title, abstract and author keywords - adjx = adjacent within x words, regardless of order - * = truncation of word for alternate endings |
| --- | --- |
| 1. exp Neurodevelopmental disorders/  2. Problem Behavior/  3. ((attention deficit or behavio?r* or communication or conduct or fluency or intellectual* or hyperkinetic or hyperactiv* or learning or motor skill* or neurodevelopmental or neuropsychiatric* or neuro-psychiatric* or pervasive developmental or reading or speech or sound or tic) adj3 (deficit* or disabilit* or disorder* or disease* or dysfunction* or impairment* or syndrome*)).ti,ab,kf.  4. (acalculi* or adhd or asperger* or asd or autis* or childhood schizophrenia or developmental disabilit* or behavio?r* development* or dyscalculi* or dyslexi* or dyslectic or mental* retard* or mutism or pdd or tourette*).ti,ab,kf.  5. or/1-4    6. Twin Study.pt.  7. exp Twins/  8. Diseases in Twins/  9. Siblings/  10. (twin* or sibling* or sibpair* or sister* or brother* or family stud*).ti,ab,kf.  11. or/6-10    12. 5 and 11  13. Environmental Exposure/  14. Environmental Pollution/  15. Environment/  16. Social Environment/  17. Gene-Environment Interaction/  18. Inhalation Exposure/  19. exp Radiation Exposure/  20. exp Toxic Actions/  21. exp Water Pollution/  22. exp Air Pollution/  23. Maternal Exposure/  24. Paternal Exposure/  25. exp Prenatal Injuries/  26. exp Pregnancy Complications/  27. Birth Weight/  28. exp Infant, Low Birth Weight/  29. exp Infant, Premature/  30. exp Fetal Diseases/  31. exp Infant, Newborn, Diseases/  32. exp Infant Nutrition Disorders/  33. exp Gastrointestinal Diseases/  34. Gastrointestinal Microbiome/  35. Antibodies, Antinuclear/  36. exp "Tobacco Use"/  37. exp Tobacco Products/  38. exp Alcohol-Related Disorders/  39. exp Alcohol Drinking/  40. Ethanol/  41. exp Antidepressive Agents/  42. exp Testosterone/  43. exp Anesthesia/  44. exp Anesthetics/  45. Valproic Acid/  46. exp Pesticides/  47. Risk Factors/  48. (adverse effects or poisoning or toxicity).fs.  49. (etiolo* or aetiolo*).ti,ab,kf.  50. exposure*.ti,ab,kf.  51. ((pregnan* or labor or labour or delivery or obstetric* or perinatal or prenatal or postnatal or neonatal or epigenetic or maternal or paternal) adj3 (complication* or factor* or risk* or advers*)).ti,ab,kf.  52. (environment* adj3 (complication* or factor* or risk* or cause* or influence* or contribution* or mechanism* or effect* or mediat* or interaction* or correlation*)).ti,ab,kf.  53. (Gene x Environment or GenexEnvironment or Gene-environment).ti,ab,kf.  54. anti-SSA*.ti,ab,kf.  55. pesticide*.ti,ab,kf.  56. antidepress*.ti,ab,kf.  57. ((pregnan* or perinatal or prenatal) adj3 alcohol*).ti,ab,kf.  58. (gestational diabetes or maternal obesity or maternal overweight).ti,ab,kf.  59. risk factor*.ti,ab,kf.  60. (smoking or nicotine or tobacco).ti,ab,kf.  61. (birth weight or birthweight or fetal growth or foetal growth or preterm birth* or breech presentation* or cesarean section* or caesarean section* or birth injur*).ti,ab,kf.  62. testosterone*.ti,ab,kf.  63. (anesthesia* or anesthetic*).ti,ab,kf.  64. (valproic acid or valproate).ti,ab,kf.  65. congenital hyperinsulinism.ti,ab,kf.  66. (malnourish* or malnutrition*).ti,ab,kf.  67. ((gastrointestinal or gut) adj3 (disease* or disorder* or dysfunction* or problem*)).ti,ab,kf.  68. ((fecal or faecal or gut) adj3 (microbiota or microflora)).ti,ab,kf.  69. or/13-68    70. 12 and 69    71. (animals not humans).sh.    72. 70 not 71  73. remove duplicates from 72    74. exp *Neurodevelopmental disorders/et  75. exp *Neurodevelopmental disorders/ci  76. exp *Neurodevelopmental disorders/mi  77. exp *Neurodevelopmental disorders/vi  78. exp *Neurodevelopmental disorders/ps  79. or/74-78  80. 79 and 11  81. 80 not 71  82. remove duplicates from 81    83. 73 or 82  84. limit 83 to (case reports or comment or editorial or letter or news)    85. 83 not 84 | |

2. PsycInfo (Ovid)

| Interface: Ovid  Date of Search: 5 October 2017  Number of hits: 1,899 | Field labels:   - exp/ = exploded heading - / = non-exploded heading - ti,ab,id = title, abstract and author keywords - adjx = adjacent within x words, regardless of order - * = truncation of word for alternate endings |
| --- | --- |
| 1. exp Neurodevelopmental Disorders/  2. exp Attention Deficit Disorder/  3. Conduct Disorder/  4. Behavior Disorders/  5. exp Behavior Problems/  6. exp Autism Spectrum Disorders/  7. exp Learning Disorders/  8. Acalculia/  9. Communication Disorders/  10. exp Intellectual Development Disorder/  11. exp Developmental Disabilities/  12. Tics/  13. exp Tourette Syndrome/  14. exp Mutism/  15. Childhood Schizophrenia/  16. ((attention deficit or behavio?r* or communication or conduct or fluency or intellectual* or hyperkinetic or hyperactiv* or learning or motor skill* or neurodevelopmental or neuropsychiatric* or neuro-psychiatric* or pervasive developmental or reading or speech or sound or tic) adj3 (deficit* or disabilit* or disorder* or disease* or dysfunction* or impairment* or syndrome*)).ti,ab,id.  17. (acalculi* or adhd or asperger* or asd or autis* or childhood schizophrenia or developmental disabilit* or behavio?r* development* or dyscalculi* or dyslexi* or dyslectic or mental* retard* or mutism or pdd or tourette*).ti,ab,id.  18. or/1-17    19. limit 18 to 2200 twin study  20. exp siblings/  21. (twin* or sibling* or sibpair* or sister* or brother* or family stud*).ti,ab,id.  22. or/19-21    23. 18 and 22  24. environmental effects/  25. pollution/  26. environment/  27. social environments/  28. exp chemical exposure/  29. radiation/  30. exp toxicity/  31. exp Hazardous Materials/  32. carcinogens/  33. exposure/  34. prenatal exposure/  35. exp obstetrical complications/  36. birth injuries/  37. birth weight/  38. premature birth/  39. exp drug induced congenital disorders/  40. exp gastrointestinal disorders/  41. exp tobacco smoking/  42. smokeless tobacco/  43. exp alcohol drinking patterns/  44. exp alcohols/  45. exp antidepressant drugs/  46. testosterone/  47. exp anesthetic drugs/  48. valproic acid/  49. risk factors/  50. "side effects (drug)"/  51. toxic disorders/  52. (etiolo* or aetiolo*).ti,ab,id.  53. exposure*.ti,ab,id.  54. ((pregnan* or labor or labour or delivery or obstetric* or perinatal or prenatal or postnatal or neonatal or epigenetic or maternal or paternal) adj3 (complication* or factor* or risk* or advers*)).ti,ab,id.  55. (environment* adj3 (complication* or factor* or risk* or cause* or influence* or contribution* or mechanism* or effect* or mediat* or interaction* or correlation*)).ti,ab,id.  56. (Gene x Environment or GenexEnvironment or Gene-environment).ti,ab,id.  57. anti-SSA*.ti,ab,id.  58. pesticide*.ti,ab,id.  59. antidepress*.ti,ab,id.  60. ((pregnan* or perinatal or prenatal) adj3 alcohol*).ti,ab,id.  61. (gestational diabetes or maternal obesity or maternal overweight).ti,ab,id.  62. risk factor*.ti,ab,id.  63. (smoking or nicotine or tobacco).ti,ab,id.  64. (birth weight or birthweight or fetal growth or foetal growth or preterm birth* or breech presentation* or cesarean section* or caesarean section* or birth injur*).ti,ab,id.  65. testosterone*.ti,ab,id.  66. (anesthesia* or anesthetic*).ti,ab,id.  67. (valproic acid or valproate).ti,ab,id.  68. congenital hyperinsulinism.ti,ab,id.  69. (malnourish* or malnutrition*).ti,ab,id.  70. ((gastrointestinal or gut) adj3 (disease* or disorder* or dysfunction* or problem*)).ti,ab,id.  71. ((fecal or faecal or gut) adj3 (microbiota or microflora)).ti,ab,id.  72. or/24-71  73. 23 and 72  74. limit 73 to ("comment/reply" or editorial or letter)    75. 73 not 74 | |

3. Embase

| Interface: embase.com  Date of Search: 5 October 2017  Number of hits: 4,225  Comment: Emtree is the controlled vocabulary in Embase | Field labels   - /exp = exploded Emtree term - /de = non exploded Emtree term - ti,ab,kw = title, abstract and author keywords - NEAR/x = adjacent within x words, regardless of order - * = truncation of word for alternate endings |
| --- | --- |
| \| 1 \| 'autism'/exp \| \| --- \| --- \| \| 2 \| 'behavior disorder'/de \| \| 3 \| 'attention deficit disorder'/de \| \| 4 \| 'disruptive behavior'/exp \| \| 5 \| 'conduct disorder'/de \| \| 6 \| 'communication disorder'/de \| \| 7 \| 'fluency disorder'/de \| \| 8 \| 'speech sound disorder'/de \| \| 9 \| 'developmental disorder'/exp \| \| 10 \| 'intellectual impairment'/de \| \| 11 \| 'learning disorder'/exp \| \| 12 \| 'dyslexia'/de \| \| 13 \| 'psychomotor disorder'/de \| \| 14 \| 'mutism'/exp \| \| 15 \| 'motor dysfunction'/de \| \| 16 \| 'tic'/exp \| \| 117 \| (('attention deficit' OR behavio* OR communication OR conduct OR fluency OR intellectual* OR hyperkinetic OR hyperactiv* OR learning OR 'motor skill*' OR neurodevelopmental OR neuropsychiatric* OR 'neuro-psychiatric*' OR 'pervasive developmental' OR reading OR speech OR sound OR tic) NEAR/3 (deficit* OR disabilit* OR disorder* OR disease* OR dysfunction* OR impairment* OR syndrome*)):ab,ti,kw \| \| 118 \| acalculi*:ab,ti,kw OR adhd:ab,ti,kw OR asperger*:ab,ti,kw OR asd:ab,ti,kw OR autis*:ab,ti,kw OR 'childhood schizophrenia':ab,ti,kw OR 'developmental disabilit*':ab,ti,kw OR 'behavio* development*':ab,ti,kw OR dyscalculi*:ab,ti,kw OR dyslexi*:ab,ti,kw OR dyslectic:ab,ti,kw OR 'mental* retard*':ab,ti,kw OR mutism:ab,ti,kw OR pdd:ab,ti,kw OR tourette*:ab,ti,kw \| \| 119 \| #1 OR #2 OR #3 OR #4 OR #5 OR #6 OR #7 OR #8 OR #9 OR #10 OR #11 OR #12 OR #13 OR #14 OR #15 OR #16 OR #17 OR #18 \| \|  \|  \| \| 20 \| 'twin study'/exp \| \| 21 \| 'twins'/exp \| \| 22 \| 'sibling'/exp \| \| 23 \| twin*:ab,ti,kw OR sibling*:ab,ti,kw OR sibpair*:ab,ti,kw OR sister*:ab,ti,kw OR brother*:ab,ti,kw OR 'family stud*':ab,ti,kw \| \| 24 \| #20 OR #21 OR #22 OR #23 \| \|  \|  \| \| 25 \| #19 AND #24 \| \|  \|  \| \| 26 \| 'environmental exposure'/de \| \| 27 \| 'pollution'/de \| \| 28 \| 'environment'/de \| \| 29 \| 'social environment'/de \| \| 30 \| 'genotype environment interaction'/de \| \| 31 \| 'exposure'/de \| \| 32 \| 'radiation exposure'/exp \| \| 33 \| 'radiation injury'/exp \| \| 34 \| 'toxicity'/exp \| \| 35 \| 'endocrine disruptor'/de \| \| 36 \| 'pollutant'/exp \| \| 37 \| 'dangerous goods'/de \| \| 38 \| 'hazardous waste'/exp \| \| 39 \| 'toxic substance'/exp \| \| 40 \| 'environmental chemical'/exp \| \| 41 \| 'water pollution'/exp \| \| 42 \| 'air pollution'/exp \| \| 43 \| 'radioactive pollution'/de \| \| 44 \| 'soil pollution'/exp \| \| 45 \| 'maternal exposure'/de \| \| 46 \| 'paternal exposure'/de \| \| 47 \| 'perinatal drug exposure'/de \| \| 48 \| 'prenatal drug exposure'/de \| \| 49 \| 'prenatal exposure'/de \| \| 50 \| 'prenatal disorder'/exp \| \| 51 \| 'pregnancy disorder'/exp \| \| 52 \| 'birth weight'/exp \| \| 53 \| 'prematurity'/de \| \| 54 \| 'newborn disease'/exp \| \| 55 \| 'gastrointestinal disease'/de \| \| 56 \| 'esophagus disease'/exp \| \| 57 \| 'gastritis'/exp \| \| 58 \| 'gastrointestinal hemorrhage'/exp \| \| 59 \| 'enteropathy'/exp \| \| 60 \| 'stomach disease'/exp \| \| 61 \| 'intestine flora'/exp \| \| 62 \| 'la antibody'/de \| \| 63 \| 'tobacco use'/exp \| \| 64 \| 'tobacco'/de \| \| 65 \| 'alcoholism'/de \| \| 66 \| 'alcohol abuse'/exp \| \| 67 \| 'alcohol'/de \| \| 68 \| 'antidepressant agent'/exp \| \| 69 \| 'testosterone'/de \| \| 70 \| 'anesthesia'/exp \| \| 71 \| 'anesthetic agent'/exp \| \| 72 \| 'valproic acid'/de \| \| 73 \| 'risk factor'/de \| \| 74 \| 'adverse drug reaction':lnk \| \| 75 \| 'drug toxicity':lnk \| \| 76 \| etiolo*:ab,ti,kw OR aetiolo*:ab,ti,kw \| \| 77 \| exposure*:ab,ti,kw \| \| 778 \| ((pregnan* OR labor OR labour OR delivery OR obstetric* OR perinatal OR prenatal OR postnatal OR neonatal OR epigenetic OR maternal OR paternal) NEAR/3 (complication* OR factor* OR risk* OR advers*)):ab,ti,kw \| \| 779 \| (environment* NEAR/3 (complication* OR factor* OR risk* OR cause* OR influence* OR contribution* OR mechanism* OR effect* OR mediat* OR interaction* OR correlation*)):ab,ti,kw \| \| 80 \| 'gene x environment':ab,ti,kw OR genexenvironment:ab,ti,kw OR 'gene-environment':ab,ti,kw \| \| 81 \| 'anti-ssa*':ab,ti,kw \| \| 82 \| pesticide*:ab,ti,kw \| \| 83 \| antidepress*:ab,ti,kw \| \| 84 \| ((pregnan* OR perinatal OR prenatal) NEAR/3 alcohol*):ab,ti,kw \| \| 85 \| 'gestational diabetes':ab,ti,kw OR 'maternal obesity':ab,ti,kw OR 'maternal overweight':ab,ti,kw \| \| 86 \| 'risk factor*':ab,ti,kw \| \| 87 \| smoking:ab,ti,kw OR nicotine:ab,ti,kw OR tobacco:ab,ti,kw \| \| 888 \| 'birth weight':ab,ti,kw OR birthweight:ab,ti,kw OR 'fetal growth':ab,ti,kw OR 'foetal growth':ab,ti,kw OR 'preterm birth*':ab,ti,kw OR 'breech presentation*':ab,ti,kw OR 'cesarean section*':ab,ti,kw OR 'caesarean section*':ab,ti,kw OR 'birth injur*':ab,ti,kw \| \| 89 \| testosterone*:ab,ti,kw \| \| 90 \| anesthesia*:ab,ti,kw OR anesthetic*:ab,ti,kw \| \| 91 \| 'valproic acid':ab,ti,kw OR valproate:ab,ti,kw \| \| 92 \| 'congenital hyperinsulinism':ab,ti,kw \| \| 93 \| malnourish*:ab,ti,kw OR malnutrition*:ab,ti,kw \| \| 94 \| ((gastrointestinal OR gut) NEAR/3 (disease* OR disorder* OR dysfunction* OR problem*)):ab,ti,kw \| \| 95 \| ((fecal OR faecal OR gut) NEAR/3 (microbiota OR microflora)):ab,ti,kw \| \| 996 \| #26 OR #27 OR #28 OR #29 OR #30 OR #31 OR #32 OR #33 OR #34 OR #35 OR #36 OR #37 OR #38 OR #39 OR #40 OR #41 OR #42 OR #43 OR #44 OR #45 OR #46 OR #47 OR #48 OR #49 OR #50 OR #51 OR #52 OR #53 OR #54 OR #55 OR #56 OR #57 OR #58 OR #59 OR #60 OR #61 OR #62 OR #63 OR #64 OR #65 OR #66 OR #67 OR #68 OR #69 OR #70 OR #71 OR #72 OR #73 OR #74 OR #75 OR #76 OR #77 OR #78 OR #79 OR #80 OR #81 OR #82 OR #83 OR #84 OR #85 OR #86 OR #87 OR #88 OR #89 OR #90 OR #91 OR #92 OR #93 OR #94 OR #95 \| \|  \|  \| \| 97 \| #25 AND #96 \| \|  \|  \| \| 98 \| [animals]/lim NOT [humans]/lim \| \|  \|  \| \| 99 \| #97 NOT #98 \| \|  \|  \| \| 100 \| 'autism'/exp/dm_et \| \| 101 \| 'behavior disorder'/dm_et \| \| 102 \| 'attention deficit disorder'/dm_et \| \| 103 \| 'disruptive behavior'/exp/dm_et \| \| 104 \| 'conduct disorder'/dm_et \| \| 105 \| 'communication disorder'/dm_et \| \| 106 \| 'fluency disorder'/dm_et \| \| 107 \| 'speech sound disorder'/dm_et \| \| 108 \| 'developmental disorder'/exp/dm_et \| \| 109 \| 'intellectual impairment'/dm_et \| \| 110 \| 'learning disorder'/exp/dm_et \| \| 111 \| 'dyslexia'/dm_et \| \| 112 \| 'psychomotor disorder'/dm_et \| \| 113 \| 'mutism'/exp/dm_et \| \| 114 \| 'motor dysfunction'/dm_et \| \| 115 \| 'tic'/exp/dm_et \| \| 1116 \| #100 OR #101 OR #102 OR #103 OR #104 OR #105 OR #106 OR #107 OR #108 OR #109 OR #110 OR #111 OR #112 OR #113 OR #114 OR #115 \| \|  \|  \| \| 117 \| #24 AND #116 \| \|  \|  \| \| 118 \| #117 NOT #98 \| \|  \|  \| \| 119 \| #99 OR #118 \| \|  \|  \| \| 1120 \| 'case report'/de NOT ('clinical study'/de OR 'case control study'/exp OR 'case study'/de OR 'clinical article'/de OR 'clinical trial'/exp OR 'community trial'/de OR 'family study'/de OR 'intervention study'/de OR 'longitudinal study'/exp OR 'major clinical study'/de OR 'open study'/de OR 'postmarketing surveillance'/exp OR 'prospective study'/de OR 'retrospective study'/de OR 'comparative study'/exp OR 'controlled study'/exp OR 'experimental study'/exp OR 'observational study'/de OR 'pilot study'/de OR 'quasi experimental study'/de OR 'twin study'/de) \| \| 121 \| 'conference abstract'/it OR 'editorial'/it OR 'letter'/it \| \| 122 \| #120 OR #121 \| \|  \|  \| \| 123 \| #119 NOT #122 \| | |

4. Web of Science Core Collection

| Interface: Clarivate Analytics  Date of Search: 5 October 2017  Number of hits: 2,642 | Field labels:   - TS = Topic = title, abstract & keyword - NEAR/x = adjacent within x words - * = truncation of word for alternate endings |
| --- | --- |
| **TOPIC:** (("attention deficit" or "behavio$r*" or "communication" or "conduct" or "fluency" or "intellectual*" or "hyperkinetic" or "hyperactiv*" or "learning" or "motor skill*" or "neurodevelopmental" or "neuropsychiatric*" or "neuro-psychiatric*" or "pervasive developmental" or "reading" or "speech" or "sound" or "tic") NEAR/3 ("deficit*" or "disabilit*" or "disorder*" or "disease*" or "dysfunction*" or "impairment*" or "syndrome*")) **OR** **TOPIC:** ("acalculi*" or "adhd" or "asperger*" or "asd" or "autis*" or "childhood schizophrenia" or "developmental disabilit*" or "behavio$r* development*" or "dyscalculi*" or "dyslexi*" or "dyslectic" or "mental* retard*" or "mutism" or "pdd" or "tourette*")  **AND**  **TOPIC:** ("twin*" or "sibling*" or "sibpair*" or "sister*" or "brother*" or "family stud*")  **AND**  **TOPIC:** ("etiolo*" or "aetiolo*" or "exposure*" or "Gene x Environment" or "GenexEnvironment" or "Gene-environment" or "anti-SSA*" or "pesticide*" or "antidepress*" or "gestational diabetes" or "maternal obesity" or "maternal overweight" or "risk factor*" or "smoking" or "nicotine" or "tobacco" or "birth weight" or "birthweight" or "fetal growth" or "foetal growth" or "preterm birth*" or "breech presentation*" or "cesarean section*" or "caesarean section*" or "birth injur*" or "testosterone*" or "anesthesia*" or "anesthetic*" or "valproic acid" or "valproate" or "congenital hyperinsulinism" or "malnourish*" or "malnutrition*") **OR** **TOPIC:** (("pregnan*" or "labor" or "labour" or "delivery" or "obstetric*" or "perinatal" or "prenatal" or "postnatal" or "neonatal" or "epigenetic" or "maternal" or "paternal") NEAR/3 ("complication*" or "factor*" or "risk*" or "advers*")) **OR** **TOPIC:** ("environment*" NEAR/3 ("complication*" or "factor*" or "risk*" or "cause*" or "influence*" or "contribution*" or "mechanism*" or "effect*" or "mediat*" or "interaction*" or "correlation*")) **OR** **TOPIC:** (("pregnan*" or "perinatal" or "prenatal") NEAR/3 "alcohol*") **OR** **TOPIC:** (("gastrointestinal" or "gut") NEAR/3 ("disease*" or "disorder*" or "dysfunction*" or "problem*")) **OR** **TOPIC:** (("fecal" or "faecal" or "gut") NEAR/3 ("microbiota" or "microflora"))  Refined by: [excluding] DOCUMENT TYPES: ( EDITORIAL MATERIAL OR LETTER OR MEETING ABSTRACT OR NEWS ITEM ) | |

5. Cochrane Library

| Interface: Wiley  Date of Search: 5 October 2017  Number of hits: 26 | Field labels   - ab,ti = title & abstract - near/x = adjacent within x words, regardless of order - * = truncation of word for alternate endings |
| --- | --- |
| #1 ("attention deficit" or behavio* or communication or conduct or fluency or intellectual* or hyperkinetic or hyperactiv* or learning or "motor skill*" or neurodevelopmental or neuropsychiatric* or "neuro-psychiatric*" or "pervasive developmental" or reading or speech or sound or tic) near/3 (deficit* or disabilit* or disorder* or disease* or dysfunction* or impairment* or syndrome*):ab,ti  #2 (acalculi* or adhd or asperger* or asd or autis* or "childhood schizophrenia" or "developmental disabilit*" or "behavio* development*" or dyscalculi* or dyslexi* or dyslectic or "mental* retard*" or mutism or pdd or tourette*):ab,ti  #3 #1 or #2  #4 (twin* or sibling* or sibpair* or sister* or brother* or "family stud*"):ab,ti  #5 (etiolo* or aetiolo* or exposure* or "Gene x Environment" or GenexEnvironment or "Gene-environment" or "anti-SSA*" or pesticide* or antidepress* or "gestational diabetes" or "maternal obesity" or "maternal overweight" or "risk factor*" or smoking or nicotine or tobacco or "birth weight" or birthweight or "fetal growth" or "foetal growth" or "preterm birth*" or "breech presentation*" or "cesarean section*" or "caesarean section*" or "birth injur*" or testosterone* or anesthesia* or anesthetic* or "valproic acid" or valproate or "congenital hyperinsulinism" or malnourish* or malnutrition*):ab,ti  #6 ((pregnan* or labor or labour or delivery or obstetric* or perinatal or prenatal or postnatal or neonatal or epigenetic or maternal or paternal) near/3 (complication* or factor* or risk* or advers*)):ab,ti  #7 (environment* near/3 (complication* or factor* or risk* or cause* or influence* or contribution* or mechanism* or effect* or mediat* or interaction* or correlation*)):ab,ti  #8 ((pregnan* or perinatal or prenatal) near/3 alcohol*):ab,ti  #9 ((gastrointestinal or gut) near/3 (disease* or disorder* or dysfunction* or problem*)):ab,ti  #10 ((fecal or faecal or gut) near/3 (microbiota or microflora)):ab,ti  #11 #5 or #6 or #7 or #8 or #9 or #10  #12 #3 and #4 and #11 | |
