# Supplementary material for: Early environmental risk factors for neurodevelopmental disorders – a systematic review of twin and sibling studies
Source: Dev Psychopathol. 2021 Oct;33(4):1448–95. doi: 10.1017/S0954579420000620 (PMC8564717; doi:10.1017/S0954579420000620)
Supplement: Supplementary file 1 [file S0954579420000620sup.zip › S0954579420000620sup002.docx]

**Appendix 2. List of excluded articles with reason**

| Author | Year | Reason for exclusion | Comment |
| --- | --- | --- | --- |
| Alemany | 2013 | Inadequate outcome | Too broad. Only Strength and Difficulties Questionaire total-score, not subscale for hyperactivity |
| Antoniou | 2014 | Inadequate study design |  |
| Antshel | 2003 | Duplication |  |
| Axelsson | 2019 | Duplication |  |
| Babinska | 2017 | Inadequate age of exposure |  |
| Bailet | 2000 | Inadequate age of exposure |  |
| Barnes | 2013 | Inadequate outcome |  |
| Bartels | 2009 | Inadequate outcome |  |
| Biederman | 2009 | Inadequate study design | Comparisons between families only |
| Boisvert | 2008 | Inadequate age of exposure |  |
| Bornovalova | 2014 | Inadequate age of exposure |  |
| Brimacombe | 2007 | Inadequate study design and control group |  |
| Butwicka | 2015 | Inadequate study design | No intra-sibling comparison |
| Butwicka | 2017 | Inadequate age of exposure |  |
| Carballo | 2013 | Inadequate study design | No sibling comparison |
| Carlton-Conway | 2005 | Inadequate age of exposure |  |
| Caspi | 2000 | Inadequate outcome | No standardized outcome measure |
| Castellheim | 2018 | Inadequate age of exposure |  |
| Cecil | 2012 | Inadequate outcome |  |
| Cecil | 2012 | Duplication |  |
| Claassen | 2008 | Inadequate study design | Case study |
| Cordova-Palomera | 2015 | Inadequate outcome |  |
| Cordova-Palomera | 2014 | Inadequate outcome |  |
| Crawford | 1995 | Inadequate age of exposure |  |
| Croen | 2019 | Inadequate study design |  |
| Curtin | 2018 | Inadequate study design |  |
| D'Onofrio | 2009 | Inadequate outcome |  |
| Danese | 2017 | Inadequate age of exposure |  |
| Davis | 1987 | Inadequate outcome |  |
| De Santis | 2017 | Inadequate age of exposure |  |
| De Santis | 2017 | Inadequate age of exposure |  |
| DiMaggio | 2011 | Inadequate outcome |  |
| Dinkler | 2017 | Inadequate age of exposure |  |
| Dostanic | 2018 | Inadequate study design |  |
| Ee | 2014 | Inadequate age of exposure |  |
| Ekblad | 2017 | Inadequate outcome |  |
| Fletcher | 2011 | Inadequate study design | No intra-sibling comparison |
| Foley | 2004 | Inadequate age of exposure |  |
| Forrest | 1968 | Only protocol |  |
| Gardner | 1998 | Inadequate age of exposure |  |
| Geier | 2007 | Inadequate age of exposure |  |
| Gittelman | 1983 | Inadequate age of exposure |  |
| Gondalia | 2012 | Inadequate age of exposure |  |
| Gong | 2014 | Inadequate study design | No intra-twin comparison. |
| Grjibovski | 2005 | Inadequate outcome |  |
| Groen-Blokhuis | 2011 | No exposure |  |
| Groen-Blokhuis | 2011 | Duplication |  |
| Grossi | 2016 | Pilot study |  |
| Hendrix | 2011 | No peer review |  |
| Honma | 1999 | Inadequate study design | No intra-twin comparison |
| Huijbregts | 2007 | Inadequate study design |  |
| Hviid | 2019 | Inadequate study design |  |
| Jackson | 2013 | Inadequate age of exposure |  |
| Jain | 2015 | Inadequate study design |  |
| Khaled | 2016 | Inadequate age of exposure | In sibling control group |
| King | 2000 | Inadequate age of exposure |  |
| Knopik | 2005 | Inadequate study design |  |
| Knopik | 2009 | Inadequate study design | No intra-pair comparison |
| Kollins | 2009 | Inadequate age of exposure |  |
| Levy-Zaks | 2014 | Inadequate study design |  |
| Lie | 2017 | Inadequate outcome | Inadequate outcome for the sibling comparison |
| Lundstrom | 2010 | Inadequate study design | No intra-pair comparison |
| Lussu | 2017 | Inadequate age of exposure |  |
| Mankuta | 2010 | Inadequate outcome |  |
| Marin | 2014 | Inadequate study design | No intra-sibling comparison |
| Mascheretti | 2013 | Inadequate study design |  |
| Massey | 1997 | Only abstract |  |
| McGrath | 2007 | Inadequate study design |  |
| Melnyk | 2012 | Inadequate age of exposure |  |
| Micalizzi | 2018 | Inadequate outcome |  |
| Milberger | 1996 | Inadequate study design |  |
| Mulligan | 2013 | Inadequate age of exposure |  |
| Mura | 1974 | Inadequate outcome |  |
| Napolioni | 2013 | Inadequate age of exposure |  |
| Neufeld | 2008 | Inadequate study design |  |
| Neuman | 2007 | Inadequate study design | No intra-pair comparison |
| O'Leary | 2019 | Inadequate outcome | Categorical measure not equivalent with a DSM 5 diagnosis |
| Ortibus | 2009 | Inadequate study design | No twin comparison |
| Palmer | 2016 | Inadequate study design | No intra-twin comparison |
| Park | 2001 | Inadequate study design |  |
| Parracho | 2005 | Inadequate age of exposure |  |
| Pearsall-Jones | 2009 | Inadequate outcome | No odds ratio between trait and exposure |
| Petik | 2008 | Inadequate outcome |  |
| Pike | 2006 | Inadequate study design |  |
| Piras | 2014 | Inadequate study design |  |
| Plamondon | 2018 | Inadequate study design |  |
| Quadros | 2018 | Inadequate age of exposure | Unclear age of exposure <5 years |
| Reddick | 2014 | Inadequate age of exposure |  |
| Regehr | 1988 | No exposure |  |
| Rooney | 2003 | Inadequate study design |  |
| Rotem | 2018 | Inadequate study design |  |
| Rydhstrom | 1990 | Inadequate study design |  |
| Safer | 1973 | Inadequate outcome | No measure of association |
| Schou | 1976 | Inadequate outcome |  |
| Sharman | 2009 | Inadequate age of exposure |  |
| Shen | 2011 | Inadequate age of exposure |  |
| Singh | 2003 | Inadequate age of exposure |  |
| Smith | 2014 | Inadequate study design |  |
| Spiker | 2001 | Inadequate study design |  |
| Stern | 2018 | Inadequate age of exposure |  |
| Sujan | 2017 | Duplication |  |
| Taylor | 1993 | Inadequate outcome |  |
| Taylor | 2018 | Inadequate outcome |  |
| Titman | 2008 | Inadequate age of exposure |  |
| Tollanes | 2016 | No exposure |  |
| Tomova | 2015 | Inadequate age of exposure |  |
| Tully | 2004 | Inadequate study design and control group |  |
| Turan Akyol | 2015 | Inadequate age of exposure |  |
| van Os | 2001 | Inadequate outcome | Too broad. Child Behavioral Checklist total-score only |
| Wagner | 2009 | Inadequate study design |  |
| Whitmore | 1993 | Inadequate age of exposure |  |
| Wichers | 2002 | Inadequate outcome | Too broad. Child Behavioral Checklist total-score only |
| Wright | 2012 | Inadequate age of exposure |  |
| Yenkoyan | 2018 | Inadequate age of exposure |  |

| **Summary** | **Number** |
| --- | --- |
| Age of exposure | 39 |
| Study design | 36 |
| Outcome | 25 |
| Duplication | 5 |
| Exposure | 3 |
| Study design and control group | 2 |
| No peer review | 1 |
| Only protocol | 1 |
| Only abstract | 1 |
| Pilot study | 1 |
| **In total** | **114** |

**References**

Alemany, S., Rijsdijk, F. V., Haworth, C. M., Fananas, L., & Plomin, R. (2013). Genetic origin of the relationship between parental negativity and behavior problems from early childhood to adolescence: a longitudinal genetically sensitive study. *Development & Psychopathology, 25*(2), 487-500. doi:https://dx.

Antoniou, E. E., Fowler, T., Reed, K., Southwood, T. R., McCleery, J. P., & Zeegers, M. P. (2014). Maternal pre-pregnancy weight and externalising behaviour problems in preschool children: a UK-based twin study. *BMJ Open, 4*(10), e005974.

Antshel, K. M., & Waisbren, S. E. (2003). Developmental timing of exposure to elevated levels of phenylalanine is associated with ADHD symptom expression. *Journal of Abnormal Child Psychology, 31*(6), 565-574.

Axelsson, P. B., Clausen, T. D., Petersen, A. H., Hageman, I., Pinborg, A., Kessing, L. V., . . . Lokkegaard, E. C. L. (2019). Relation Between Infant Microbiota and Autism? Results from a National Cohort Sibling Design Study. *Epidemiology (Cambridge, Mass.), 30*(1), 52-60.

Babinska, K., Tomova, A., Celusakova, H., Babkova, J., Repiska, G., Kubranska, A., . . . Ostatnikova, D. (2017). Fecal calprotectin levels correlate with main domains of the autism diagnostic interview-revised (ADI-R) in a sample of individuals with autism spectrum disorders from Slovakia. *Physiological research, 66*(Supplementum 4), S517-S522.

Bailet, L. L., & Turk, W. R. (2000). The impact of childhood epilepsy on neurocognitive and behavioral performance: a prospective longitudinal study. *Epilepsia, 41*(4), 426-431.

Barnes, J. C., Boutwell, B. B., Beaver, K. M., & Gibson, C. L. (2013). Analyzing the origins of childhood externalizing behavioral problems. *Developmental Psychology, 49*(12), 2272-2284.

Bartels, M., Althoff, R. R., & Boomsma, D. I. (2009). Anesthesia and cognitive performance in children: no evidence for a causal relationship. *Twin Research & Human Genetics: the Official Journal of the International Society for Twin Studies, 12*(3), 246-253.

Biederman, J., Monuteaux, M. C., Faraone, S. V., & Mick, E. (2009). Parsing the associations between prenatal exposure to nicotine and offspring psychopathology in a nonreferred sample. *Journal of Adolescent Health, 45*(2), 142-148.

Boisvert, D., & Wright, J. P. (2008). Nonshared environmental influences on sibling differences in externalizing problem behavior. *Criminal Justice and Behavior, 35*(7), 863-878.

Bornovalova, M. A., Cummings, J. R., Hunt, E., Blazei, R., Malone, S., & Iacono, W. G. (2014). Understanding the relative contributions of direct environmental effects and passive genotype-environment correlations in the association between familial risk factors and child disruptive behavior disorders. *Psychological Medicine, 44*(4), 831-844. doi:10.1017/s0033291713001086

Brimacombe, M., Ming, X., & Lamendola, M. (2007). Prenatal and birth complications in autism. *Maternal and Child Health Journal, 11*(1), 73-79.

Butwicka, A., Lichtenstein, P., Frisen, L., Almqvist, C., Larsson, H., & Ludvigsson, J. F. (2017). Celiac Disease Is Associated with Childhood Psychiatric Disorders: A Population-Based Study. *Journal of Pediatrics, 184*, 87-93.e81.

Butwicka, A., Lichtenstein, P., Landen, M., Nordenvall, A. S., Nordenstrom, A., Nordenskjold, A., & Frisen, L. (2015). Hypospadias and increased risk for neurodevelopmental disorders. *Journal of Child Psychology and Psychiatry, 56*(2), 155-161.

Byrne, J. M., Camfield, P. R., Clark-Touesnard, M., & Hondas, B. J. (1987). Effects of phenobarbital on early intellectual and behavioral development: a concordant twin case study. *Journal of Clinical & Experimental Neuropsychology: Official Journal of the International Neuropsychological Society, 9*(4), 393-398.

Carballo, J. J., Garcia-Nieto, R., Alvarez-Garcia, R., Caro-Canizares, I., Lopez-Castroman, J., Munoz-Lorenzo, L., . . . Baca-Garcia, E. (2013). Sibship size, birth order, family structure and childhood mental disorders. *Social Psychiatry & Psychiatric Epidemiology, 48*(8), 1327-1333.

Carlton-Conway, D., Ahluwalia, R., Henry, L., Michie, C., Wood, L., & Tulloh, R. (2005). Behaviour sequelae following acute Kawasaki disease. *BMC Pediatrics, 5*(1), 14.

Caspi, A., Taylor, A., Moffitt, T. E., & Plomin, R. (2000). Neighborhood deprivation affects children's mental health: environmental risks identified in a genetic design. *Psychological Science, 11*(4), 338-342.

Castellheim, A., Lundström, S., Molin, M., Kuja-Halkola, R., Gillberg, C., & Gillberg, C. (2018). The role of general anesthesia on traits of neurodevelopmental disorders in a Swedish cohort of twins. *Journal of Child Psychology and Psychiatry, and Allied Disciplines, 59*(9), 966-972.

Cecil, C. A., Barker, E. D., Jaffee, S. R., & Viding, E. (2012). Association between maladaptive parenting and child self-control over time: cross-lagged study using a monozygotic twin difference design. Erratum appears in Br J Psychiatry. 2012 Nov;201(5):412. *British Journal of Psychiatry, 201*(4), 291-297.

Claassen, M., Naude, H., Pretorius, E., & Bosman, M. (2008). The contribution of prenatal stress to the pathogenesis of autism as a neurobiological developmental disorder: A dizygotic twin study. *Early Child Development and Care, 178*(5), 487-511.

Cordova-Palomera, A., Alemany, S., Fatjo-Vilas, M., Goldberg, X., Leza, J. C., Gonzalez-Pinto, A., . . . Fananas, L. (2014). Birth weight, working memory and epigenetic signatures in IGF2 and related genes: a MZ twin study. *PLoS ONE [Electronic Resource], 9*(8), e103639.

Cordova-Palomera, A., Fatjo-Vilas, M., Falcon, C., Bargallo, N., Alemany, S., Crespo-Facorro, B., . . . Fananas, L. (2015). Birth Weight and Adult IQ, but Not Anxious-Depressive Psychopathology, Are Associated with Cortical Surface Area: A Study in Twins. *PLoS ONE [Electronic Resource], 10*(6), e0129616.

Crawford, S. G., Kaplan, B. J., & Field, L. L. (1995). Absence of an association between insulin-dependent diabetes mellitus and developmental learning difficulties. *Hereditas, 122*(1), 73-78.

Croen, L. A., Qian, Y., Ashwood, P., Daniels, J. L., Fallin, D., Schendel, D., . . . Zerbo, O. (2019). Family history of immune conditions and autism spectrum and developmental disorders: Findings from the study to explore early development. *Autism Research: Official Journal of the International Society for Autism Research, 12*(1), 123-135.

Curtin, P., Austin, C., Curtin, A., Gennings, C., Arora, M., Tammimies, K., . . . Reichenberg, A. (2018). Dynamical features in fetal and postnatal zinc-copper metabolic cycles predict the emergence of autism spectrum disorder. *Science Advances, 4*(5). doi:10.1126/sciadv.aat1293

D'Onofrio, B. M., Goodnight, J. A., van Hulle, C. A., Rodgers, J. L., Rathouz, P. J., Waldman, I. D., & Lahey, B. B. (2009). Maternal age at childbirth and offspring disruptive behaviors: testing the causal hypothesis. *Journal of Child Psychology and Psychiatry, and Allied Disciplines, 50*(8), 1018-1028.

Danese, A., Moffitt, T. E., Arseneault, L., Bleiberg, B. A., Dinardo, P. B., Gandelman, S. B., . . . Caspi, A. (2017). The origins of cognitive deficits in victimized children: Implications for neuroscientists and clinicians. *American Journal of Psychiatry, 174*(4), 349-361.

Davis, S. L., Tooley, W. H., & Hunt, J. V. (1987). Developmental outcome following posthemorrhagic hydrocephalus in preterm infants. Comparison of twins discordant for hydrocephalus. *American Journal of Diseases of Children, 141*(11), 1170-1174.

De Santis, B., Brera, C., Mezzelani, A., Soricelli, S., Ciceri, F., Moretti, G., . . . Raggi, M. E. (2017). Role of mycotoxins in the pathobiology of autism: A first evidence. *Nutritional Neuroscience*, 1-13.

De Santis, B., Raggi, M. E., Moretti, G., Facchiano, F., Mezzelani, A., Villa, L., . . . Brera, C. (2017). Study on the Association among Mycotoxins and other Variables in Children with Autism. *Toxins, 9*(7), 29.

DiMaggio, C., Sun, L. S., & Li, G. (2011). Early childhood exposure to anesthesia and risk of developmental and behavioral disorders in a sibling birth cohort. *Anesthesia & Analgesia, 113*(5), 1143-1151.

Dinkler, L., Lundstrom, S., Gajwani, R., Lichtenstein, P., Gillberg, C., & Minnis, H. (2017). Maltreatment-associated neurodevelopmental disorders: a co-twin control analysis. *Journal of Child Psychology and Psychiatry, and Allied Disciplines, 58*(6), 691-701.

Dostanic, T., Sustersic, B., & Paro-Panjan, D. (2018). Developmental outcome in a group of twins: Relation to perinatal factors and general movements. *European Journal of Paediatric Neurology, 22*(4), 682-689.

Ee, L. C., Lloyd, O., Beale, K., Fawcett, J., & Cleghorn, G. J. (2014). Academic potential and cognitive functioning of long-term survivors after childhood liver transplantation. *Pediatric Transplantation, 18*(3), 272-279.

Ekblad, M., Lehtonen, L., Korkeila, J., & Gissler, M. (2017). Maternal Smoking During Pregnancy and the Risk of Psychiatric Morbidity in Singleton Sibling Pairs. *Nicotine & Tobacco Research, 19*(5), 597-604.

Fletcher, J. M. (2011). The medium term schooling and health effects of low birth weight: Evidence from siblings. *Economics of Education Review, 30*(3), 517-527.

Foley, D. L., Eaves, L. J., Wormley, B., Silberg, J. L., Maes, H. H., Kuhn, J., & Riley, B. (2004). Childhood adversity, monoamine oxidase a genotype, and risk for conduct disorder. *Archives of General Psychiatry, 61*(7), 738-744.

Forrest, T., Owen, F., & Adams, P. (1968). Learning disabilities in children: sibling studies. *Developmental Medicine & Child Neurology, 10*(2), 252-253.

Gardner, N. (1998). Emotional and behavioural difficulties in children with diabetes: a controlled comparison with siblings and peers. *Child: Care, Health & Development, 24*(2), 115-128.

Geier, D. A., & Geier, M. R. (2007). A prospective study of mercury toxicity biomarkers in autistic spectrum disorders. *Journal of Toxicology & Environmental Health Part A, 70*(20), 1723-1730.

Gittelman, R., & Eskenazi, B. (1983). Lead and hyperactivity revisited. An investigation of nondisadvantaged children. *Archives of General Psychiatry, 40*(8), 827-833.

Gondalia, S. V., Palombo, E. A., Knowles, S. R., Cox, S. B., Meyer, D., & Austin, D. W. (2012). Molecular characterisation of gastrointestinal microbiota of children with autism (with and without gastrointestinal dysfunction) and their neurotypical siblings. *Autism research : Official Journal of the International Society for Autism Research, 5*(6), 419-427.

Gong, T., Almqvist, C., Bölte, S., Lichtenstein, P., Anckarsäter, H., Lind, T., . . . Pershagen, G. (2014). Exposure to air pollution from traffic and neurodevelopmental disorders in Swedish twins. *Twin Research & Human Genetics: the Official Journal of the International Society for Twin Studies, 17*(6), 553-562.

Grjibovski, A. M., Harris, J. R., & Magnus, P. (2005). Birthweight and adult health in a population-based sample of Norwegian twins. *Twin Research & Human Genetics: the Official Journal of the International Society for Twin Studies, 8*(2), 148-155.

Groen-Blokhuis, M. M., Middeldorp, C. M., Ce, M. v. B., & Boomsma, D. I. (2011). Crying without a cause and being easily upset in two-year-olds: heritability and predictive power of behavioral problems. Erratum appears in Twin Res Hum Genet. 2013 Apr;16(2):650. *Twin Research & Human Genetics: the Official Journal of the International Society for Twin Studies, 14*(5), 393-400.

Groen-Blokhuis, M. M., Middeldorp, C. M., van Beijsterveldt, C. E. M., & Boomsma, D. I. (2011). Evidence for a causal association of low birth weight and attention problems. *Journal of the American Academy of Child & Adolescent Psychiatry, 50*(12), 1247-1254.e1242.

Grossi, E., Veggo, F., Narzisi, A., Compare, A., & Muratori, F. (2016). Pregnancy risk factors in autism: a pilot study with artificial neural networks. *Pediatric Research, 79*(2), 339-347.

Hendrix, R. A. (2011). A sibling case-control study of maternal prenatal body mass index as a risk factor for autism spectrum disorder. *Dissertation Abstracts International: Section B: The Sciences and Engineering, 72*(6-B), 3380.

Honma, Y., Minakami, H., Eguchi, Y., Uchida, A., Izumi, A., & Sato, I. (1999). Relation between hemoglobin discordance and adverse outcome in monochorionic twins. *Acta Obstetricia et Gynecologica Scandinavica, 78*(3), 207-211.

Huijbregts, S. C. J., Seguin, J. R., Zoccolillo, M., Boivin, M., & Tremblay, R. E. (2007). Associations of maternal prenatal smoking with early childhood physical aggression, hyperactivity-impulsivity, and their co-occurrence. *Journal of Abnormal Child Psychology, 35*(2), 203-215.

Hviid, A., Hansen, J. V., Frisch, M., & Melbye, M. (2019). Measles, Mumps, Rubella Vaccination and Autism: A Nationwide Cohort Study. *Annals of Internal Medicine*.

Jackson, J., Titman, P., Butler, S., Bond, K., Rao, A., Veys, P., . . . Rao, K. (2013). Cognitive and psychosocial function post hematopoietic stem cell transplantation in children with hemophagocytic lymphohistiocytosis. *Journal of Allergy & Clinical Immunology, 132*(4), 889-895.e881-883.

Jain, A., Marshall, J., Buikema, A., Bancroft, T., Kelly, J. P., & Newschaffer, C. J. (2015). Autism occurrence by MMR vaccine status among US children with older siblings with and without autism. Erratum appears in JAMA. 2016 Jan 12;315(2):204; PMID: 26757477. *JAMA, 313*(15), 1534-1540.

Khaled, E. M., Meguid, N. A., Björklund, G., Gouda, A., Bahary, M. H., Hashish, A., . . . El-Bana, M. A. (2016). Altered urinary porphyrins and mercury exposure as biomarkers for autism severity in Egyptian children with autism spectrum disorder. *Metabolic Brain Disease, 31*(6), 1419-1426.

King, W. J., Schlieper, A., Birdi, N., Cappelli, M., Korneluk, Y., & Rowe, P. C. (2000). The effect of Kawasaki disease on cognition and behavior. *Archives of Pediatrics & Adolescent Medicine, 154*(5), 463-468.

Knopik, V. S., Heath, A. C., Bucholz, K. K., Madden, P. A. F., & Waldron, M. (2009). Genetic and environmental influences on externalizing behavior and alcohol problems in adolescence: a female twin study. *Pharmacology, Biochemistry, and Behavior, 93*(3), 313-321.

Knopik, V. S., Sparrow, E. P., Madden, P. A., Bucholz, K. K., Hudziak, J. J., Reich, W., . . . Heath, A. C. (2005). Contributions of parental alcoholism, prenatal substance exposure, and genetic transmission to child ADHD risk: a female twin study. *Psychological Medicine, 35*(5), 625-635.

Kollins, S. H., Garrett, M. E., McClernon, F. J., Lachiewicz, A. M., Morrissey-Kane, E., FitzGerald, D., . . . Ashley-Koch, A. E. (2009). Effects of postnatal parental smoking on parent and teacher ratings of ADHD and oppositional symptoms. *Journal of Nervous & Mental Disease, 197*(6), 442-449.

Levy-Zaks, A., Pollak, Y., & Ben-Pazi, H. (2014). Cerebral palsy risk factors and their impact on psychopathology. *Neurological Research, 36*(1), 92-94.

Lie, R. T., Moster, D., Strand, P., & Wilcox, A. J. (2017). Prenatal exposure to Chernobyl fallout in Norway: neurological and developmental outcomes in a 25-year follow-up. *European Journal of Epidemiology, 32*(12), 1065-1073.

Lundström, S., Haworth, C. M., Carlström, E., Gillberg, C., Mill, J., Rastam, M., . . . Reichenberg, A. (2010). Trajectories leading to autism spectrum disorders are affected by paternal age: findings from two nationally representative twin studies. *Journal of Child Psychology and Psychiatry, and Allied Disciplines, 51*(7), 850-856.

Lussu, M., Noto, A., Masili, A., Rinaldi, A. C., Dessi, A., De Angelis, M., . . . Francavilla, R. (2017). The urinary <sup>1</sup> H-NMR metabolomics profile of an italian autistic children population and their unaffected siblings. *Autism Research : Official Journal of the International Society for Autism Research, 10*(6), 1058-1066.

Mankuta, D., Goldner, I., & Knafo, A. (2010). Intertwin birth weight differences and conduct problems in early childhood. *Archives of Pediatrics & Adolescent Medicine, 164*(5), 457-461

Marin, A. M., Seco, F. L., Serrano, S. M., Garcia, S. A., Gaviria Gomez, A. M., & Ney, I. (2014). Do firstborn children have an increased risk of ADHD? *Journal of Attention Disorders, 18*(7), 594-597.

Mascheretti, S., Bureau, A., Battaglia, M., Simone, D., Quadrelli, E., Croteau, J., . . . Marino, C. (2013). An assessment of gene-by-environment interactions in developmental dyslexia-related phenotypes. *Genes, Brain and Behavior, 12*(1), 47-55.

Massey, D. S. (1997). Premature birth and learning disabilities: A neuropsychological investigation. *Dissertation Abstracts International: Section B: The Sciences and Engineering, 57*(8-B), 5364.

McGrath, L. M., Pennington, B. F., Willcutt, E. G., Boada, R., Shriberg, L. D., & Smith, S. D. (2007). Gene x Environment interactions in speech sound disorder predict language and preliteracy outcomes. *Development & Psychopathology, 19*(4), 1047-1072.

Melnyk, S., Fuchs, G. J., Schulz, E., Lopez, M., Kahler, S. G., Fussell, J. J., . . . Jill James, S. (2012). Metabolic imbalance associated with methylation dysregulation and oxidative damage in children with autism. *Journal of Autism and Developmental Disorders, 42*(3), 367-377.

Micalizzi, L., Marceau, K., Brick, L. A., Palmer, R. H., Todorov, A. A., Heath, A. C., . . . Knopik, V. S. (2018). Inhibitory Control in Siblings Discordant for Exposure to Maternal Smoking During Pregnancy. *Developmental Psychology, 54*(2), 199-208.

Milberger, S., Biederman, J., Faraone, S. V., Chen, L., & Jones, J. (1996). Is maternal smoking during pregnancy a risk factor for attention deficit hyperactivity disorder in children? *The American Journal of Psychiatry, 153*(9), 1138-1142.

Mulligan, A., Anney, R., Butler, L., O'Regan, M., Richardson, T., Tulewicz, E. M., . . . Gill, M. (2013). Home environment: association with hyperactivity/impulsivity in children with ADHD and their non-ADHD siblings. *Child: Care, Health & Development, 39*(2), 202-212.

Mura, E. L. (1974). Perinatal differences: a comparison of child psychiatric patients and their siblings. *Psychiatric Quarterly, 48*(2), 239-255.

Napolioni, V., Ober-Reynolds, B., Szelinger, S., Corneveaux, J. J., Pawlowski, T., Ober-Reynolds, S., . . . Huentelman, M. J. (2013). Plasma cytokine profiling in sibling pairs discordant for autism spectrum disorder. *Journal of Neuroinflammation, 10*.

Neufeld, R. E., Clark, B. G., Robertson, C. M. T., Moddemann, D. M., Dinu, I. A., Joffe, A. R., . . . Western Canadian Complex Pediatric Therapies Follow-up, G. (2008). Five-year neurocognitive and health outcomes after the neonatal arterial switch operation. *The Journal of Thoracic and Cardiovascular Surgery, 136*(6), 1413-1421.e1412.

Neuman, R. J., Lobos, E., Reich, W., Henderson, C. A., Sun, L. W., & Todd, R. D. (2007). Prenatal smoking exposure and dopaminergic genotypes interact to cause a severe ADHD subtype. *Biological Psychiatry, 61*(12), 1320-1328.

O'Leary, J. D., Janus, M., Duku, E., Wijeysundera, D. N., To, T., Li, P., . . . Crawford, M. W. (2019). Influence of Surgical Procedures and General Anesthesia on Child Development Before Primary School Entry Among Matched Sibling Pairs. *JAMA pediatrics, 173*(1), 29-36.

Ortibus, E., Lopriore, E., Deprest, J., Vandenbussche, F. P., Walther, F. J., Diemert, A., . . . Lewi, L. (2009). The pregnancy and long-term neurodevelopmental outcome of monochorionic diamniotic twin gestations: a multicenter prospective cohort study from the first trimester onward. *American Journal of Obstetrics & Gynecology, 200*(5), 494.e491-498.

Palmer, R. H., Bidwell, L. C., Heath, A. C., Brick, L. A., Madden, P. A., & Knopik, V. S. (2016). Effects of Maternal Smoking during Pregnancy on Offspring Externalizing Problems: Contextual Effects in a Sample of Female Twins. *Behavior Genetics, 46*(3), 403-415.

Park, R. J., & Bolton, P. F. (2001). Pervasive developmental disorder and obstetric complications in children and adolescents with tuberous sclerosis. *Autism, 5*(3), 237-248.

Parracho, H. M., Bingham, M. O., Gibson, G. R., & McCartney, A. L. (2005). Differences between the gut microflora of children with autistic spectrum disorders and that of healthy children. *Journal of Medical Microbiology, 54*(Pt 10), 987-991.

Pearsall-Jones, J. G., Piek, J. P., Rigoli, D., Martin, N. C., & Levy, F. (2009). An investigation into etiological pathways of DCD and ADHD using a monozygotic twin design. *Twin Research & Human Genetics: the Official Journal of the International Society for Twin Studies, 12*(4), 381-391.

Petik, D., Ács, N., Bánhidy, F., & Czeizel, A. E. (2008). A study of the potential teratogenic effect of large doses of promethazine used for a suicide attempt by 32 pregnant women. *Toxicology and Industrial Health, 24*(1-2), 87-96.

Pike, A., Iervolino, A. C., Eley, T. C., Price, T. S., & Plomin, R. (2006). Environmental risk and young children's cognitive and behavioral development. *International Journal of Behavioral Development, 30*(1), 55-66.

Piras, I. S., Haapanen, L., Napolioni, V., Sacco, R., Van de Water, J., & Persico, A. M. (2014). Anti-brain antibodies are associated with more severe cognitive and behavioral profiles in Italian children with Autism Spectrum Disorder. *Brain, Behavior, and Immunity, 38*, 91-99.

Plamondon, A., Browne, D. T., Madigan, S., & Jenkins, J. M. (2018). Disentangling Child-Specific and Family-Wide Processes Underlying Negative Mother-Child Transactions. *Journal of Abnormal Child Psychology, 46*(3), 437-447.

Quadros, E. V., Sequeira, J. M., Brown, W. T., Mevs, C., Marchi, E., Flory, M., . . . Cohen, I. L. (2018). Folate receptor autoantibodies are prevalent in children diagnosed with autism spectrum disorder, their normal siblings and parents. *Autism Research, 11*(5), 707-712.

Reddick, W. E., Taghipour, D. J., Glass, J. O., Ashford, J., Xiong, X., Wu, S., . . . Conklin, H. M. (2014). Prognostic factors that increase the risk for reduced white matter volumes and deficits in attention and learning for survivors of childhood cancers. *Pediatric Blood & Cancer, 61*(6), 1074-1079.

Regehr, S. M., & Kaplan, B. J. (1988). Reading disability with motor problems may be an inherited subtype. *Pediatrics, 82*(2), 204-210.

Rooney, R., Hay, D., & Levy, F. (2003). Small for gestational age as a predictor of behavioral and learning problems in twins. *Twin Research, 6*(1), 46-54.

Rotem, R. S., Chodick, G., Davidovitch, M., Hauser, R., Coull, B. A., & Weisskopf, M. G. (2018). Congenital Abnormalities of the Male Reproductive System and Risk of Autism Spectrum Disorders. *American Journal of Epidemiology, 187*(4), 656-663.

Rydhstrom, H. (1990). Prognosis for twins discordant in birth weight of 1.0 kg or more: the impact of cesarean section. *Journal of Perinatal Medicine, 18*(1), 31-37.

Safer, D. J. (1973). A familial factor in minimal brain dysfunction. *Behavior Genetics, 3*(2), 175-186.

Schou, M. (1976). What happened later to the lithium babies? A follow-up study of children born without malformations. *Acta Psychiatrica Scandinavica, 54*(3), 193-197.

Sharman, R., Sullivan, K., Young, R., & McGill, J. (2009). Biochemical markers associated with executive function in adolescents with early and continuously treated phenylketonuria. *Clinical Genetics, 75*(2), 169-174.

Shen, C., Zhao, X. L., Ju, W., Zou, X. B., Huo, L. R., Yan, W., . . . Zhong, N. (2011). A proteomic investigation of B lymphocytes in an autistic family: a pilot study of exposure to natural rubber latex (NRL) may lead to autism. *Journal of Molecular Neuroscience, 43*(3), 443-452.

Singh, V. K., & Jensen, R. L. (2003). Elevated levels of measles antibodies in children with autism. *Pediatric Neurology, 28*(4), 292-294.

Smith, T. F., Anastopoulos, A. D., Garrett, M. E., Arias-Vasquez, A., Franke, B., Oades, R. D., . . . Consortium, I. (2014). Angiogenic, neurotrophic, and inflammatory system SNPs moderate the association between birth weight and ADHD symptom severity. *American Journal of Medical Genetics. Part B, Neuropsychiatric Genetics: the Official Publication of the International Society of Psychiatric Genetics, 165B*(8), 691-704.

Spiker, D., Lotspeich, L. J., Dimiceli, S., Szatmari, P., Myers, R. M., & Risch, N. (2001). Birth Order Effects on Nonverbal IQ Scores in Autism Multiplex Families. *Journal of Autism and Developmental Disorders, 31*(5), 449-460. doi:10.1023/A:1012217807469

Stern, A., Agnew-Blais, J., Danese, A., Fisher, H. L., Jaffee, S. R., Matthews, T., . . . Arseneault, L. (2018). Associations between abuse/neglect and ADHD from childhood to young adulthood: A prospective nationally-representative twin study. *Child Abuse & Neglect, 81*, 274-285.

Sujan, A. C., Rickert, M. E., Oberg, A. S., Quinn, P. D., Hernandez-Diaz, S., Almqvist, C., . . . D'Onofrio, B. M. (2017). Associations of Maternal Antidepressant Use During the First Trimester of Pregnancy With Preterm Birth, Small for Gestational Age, Autism Spectrum Disorder, and Attention-Deficit/Hyperactivity Disorder in Offspring. *Obstetrical & Gynecological Survey, 72*(9), 523-524.

Taylor, C. L., Rice, M. L., Christensen, D., Blair, E., & Zubrick, S. R. (2018). Prenatal and perinatal risks for late language emergence in a population-level sample of twins at age 2. *BMC pediatrics, 18*(1).

Taylor, D. J., Nelson, J., & Howie, P. W. (1993). Neurodevelopmental disability--a sibling-control study. *Developmental Medicine & Child Neurology, 35*(11), 957-964.

Titman, P., Pink, E., Skucek, E., O'Hanlon, K., Cole, T. J., Gaspar, J., . . . Gaspar, H. B. (2008). Cognitive and behavioral abnormalities in children after hematopoietic stem cell transplantation for severe congenital immunodeficiencies. *Blood, 112*(9), 3907-3913.

Tollanes, M. C., Wilcox, A. J., Stoltenberg, C., Lie, R. T., & Moster, D. (2016). Neurodevelopmental Disorders or Early Death in Siblings of Children With Cerebral Palsy. *Pediatrics, 138*(2).

Tomova, A., Husarova, V., Lakatosova, S., Bakos, J., Vlkova, B., Babinska, K., & Ostatnikova, D. (2015). Gastrointestinal microbiota in children with autism in Slovakia. *Physiology & Behavior, 138*, 179-187.

Tully, L. A., Arseneault, L., Caspi, A., Moffitt, T. E., & Morgan, J. (2004). Does maternal warmth moderate the effects of birth weight on twins' attention-deficit/hyperactivity disorder (ADHD) symptoms and low IQ? *Journal of Consulting & Clinical Psychology, 72*(2), 218-226.

Turan Akyol, Ş., Kiliç, M., Güner Ş, N., Karakurt, M. N., Akbaş, S., Öztürk, F., & Sancak, R. (2015). Association between allergic diseases and attention deficit hyperactivity disorder in childhood. *Asim, Allerji, Immunoloji, 13*(2), 65-70.

van Os, J., Wichers, M., Danckaerts, M., Van Gestel, S., Derom, C., & Vlietinck, R. (2001). A prospective twin study of birth weight discordance and child problem behavior. *Biological Psychiatry, 50*(8), 593-599.

Wagner, A. I., Schmidt, N. L., Lemery-Chalfant, K., Leavitt, L. A., & Goldsmith, H. H. (2009). The limited effects of obstetrical and neonatal complications on conduct and attention-deficit hyperactivity disorder symptoms in middle childhood. *Journal of Developmental & Behavioral Pediatrics, 30*(3), 217-225.

Whitmore, E. A., Kramer, J. R., & Knutson, J. F. (1993). The association between punitive childhood experiences and hyperactivity. *Child Abuse & Neglect, 17*(3), 357-366.

Wichers, M. C., Purcell, S., Danckaerts, M., Derom, C., Derom, R., Vlietinck, R., & Van Os, J. (2002). Prenatal life and post-natal psychopathology: evidence for negative gene-birth weight interaction. *Psychological Medicine, 32*(7), 1165-1174.

Wright, B., Pearce, H., Allgar, V., Miles, J., Whitton, C., Leon, I., . . . Alderson-Day, B. (2012). A comparison of urinary mercury between children with autism spectrum disorders and control children. *PLoS ONE [Electronic Resource], 7*(2), e29547.

Yenkoyan, K., Harutyunyan, H., & Harutyunyan, A. (2018). A certain role of SOD/CAT imbalance in pathogenesis of autism spectrum disorders. *Free Radical Biology and Medicine, 123*, 85-95.
